# Long non-coding RNA containing ultraconserved genomic region 8 promotes bladder cancer tumorigenesis

**DOI:** 10.18632/oncotarget.7833

**Published:** 2016-03-01

**Authors:** Michele Olivieri, Matteo Ferro, Sara Terreri, Montano Durso, Alessandra Romanelli, Concetta Avitabile, Ottavio De Cobelli, Anna Messere, Dario Bruzzese, Ivan Vannini, Luciana Marinelli, Ettore Novellino, Wei Zhang, Mariarosaria Incoronato, Gennaro Ilardi, Stefania Staibano, Laura Marra, Renato Franco, Sisto Perdonà, Daniela Terracciano, Bogdan Czerniak, Giovanna L. Liguori, Vincenza Colonna, Muller Fabbri, Ferdinando Febbraio, George A. Calin, Amelia Cimmino

**Affiliations:** ^1^ Institute of Genetics and Biophysics “A. Buzzati Traverso”, National Research Council (CNR), Naples, Italy; ^2^ Division of Urology, European Institute of Oncology, Milan, Italy; ^3^ Bio-Ker S.r.l. MultiMedica S.p.A. Naples, Italy; ^4^ Department of Pharmacy, University of Naples “Federico II”, Naples, Italy; ^5^ Department of Environmental, Biological and Pharmaceutical Sciences and Technologies, Second University of Naples, Naples, Italy; ^6^ Department of Public Health, University of Naples “Federico II”, Naples, Italy; ^7^ Istituto Scientifico Romagnolo per lo Studio e la Cura dei Tumori (IRST) S.r.l. IRCCS, Gene Therapy Unit, Meldola, Italy; ^8^ Department of Pathology, The University of Texas MD Anderson Cancer Center, Houston, TX, USA; ^9^ Fondazione IRCCS SDN, Naples, Italy; ^10^ Department of Advanced Biomedical Sciences, University of Naples “Federico II”, Naples, Italy; ^11^ Division of Urology, IRCS National Tumor Institute, Naples, Italy; ^12^ Department of Physical and Mental Health and Preventive Medicine, Section of Pathology, Second University of Naples, Naples, Italy; ^13^ Department of Translational Medical Sciences, University of Naples “Federico II”, Naples, Italy; ^14^ Department of Pediatrics and Molecular Microbiology & Immunology, Norris Comprehensive Cancer Center, Keck School of Medicine, University of Southern California, Children's Center for Cancer and Blood Diseases and The Saban Research Institute, Children's Hospital Los Angeles, Los Angeles, CA, USA; ^15^ Institute of Protein Biochemistry, National Research Council (CNR), Naples, Italy; ^16^ Department of Experimental Therapeutics and The Center for RNA Interference and Non-Coding RNA, The University of Texas MD Anderson Cancer Center, Houston, TX, USA

**Keywords:** microRNA, T-UCR, bladder cancer, MMP9, CASZ1

## Abstract

Ultraconserved regions (UCRs) have been shown to originate non-coding RNA transcripts (T-UCRs) that have different expression profiles and play functional roles in the pathophysiology of multiple cancers. The relevance of these functions to the pathogenesis of bladder cancer (BlCa) is speculative. To elucidate this relevance, we first used genome-wide profiling to evaluate the expression of T-UCRs in BlCa tissues. Analysis of two datasets comprising normal bladder tissues and BlCa specimens with a custom T-UCR microarray identified ultraconserved RNA (uc.) 8+ as the most upregulated T-UCR in BlCa tissues, although its expression was lower than in pericancerous bladder tissues. These results were confirmed on BlCa tissues by real-time PCR and by *in situ* hybridization. Although uc.8+ is located within intron 1 of *CASZ1*, a zinc-finger transcription factor, the transcribed non-coding RNA encoding uc.8+ is expressed independently of *CASZ1*. *In vitro* experiments evaluating the effects of uc.8+ silencing, showed significantly decreased capacities for cancer cell invasion, migration, and proliferation. From this, we proposed and validated a model of interaction in which uc.8+ shuttles from the nucleus to the cytoplasm of BlCa cells, interacts with microRNA (miR)-596, and cooperates in the promotion and development of BlCa. Using computational analysis, we investigated the miR-binding domain accessibility, as determined by base-pairing interactions within the uc.8+ predicted secondary structure, RNA binding affinity, and RNA species abundance in bladder tissues and showed that uc.8+ is a natural decoy for miR-596. Thus uc.8+ upregulation results in increased expression of *MMP9*, increasing the invasive potential of BlCa cells. These interactions between evolutionarily conserved regions of DNA suggest that natural selection has preserved this potentially regulatory layer that uses RNA to modulate miR levels, opening up the possibility for development of useful markers for early diagnosis and prognosis as well as for development of new RNA-based cancer therapies.

## INTRODUCTION

Ultraconserved regions (UCRs) are sequences of the human genome located within intragenic and intergenic regions that are absolutely conserved (100% identity with no insertions or deletions) among orthologous regions of the human, rat, and mouse genomes [1]. Transcribed ultraconserved regions (T-UCRs) constitute a novel category of long non-coding RNAs. T-UCRs are often contained in RNA sequences that extend beyond the conserved regions described by Bejerano *et al* [1], and their functional role in the biology of cancer and development remains to be determined. Following our initial report that profiled T-UCRs for B-cell chronic lymphocytic leukemia [2], other groups profiled T-UCRs and suggested that these long non-coding RNAs could contribute to the development of pediatric tumors, neuroblastoma, and prostate cancer [3]. Researchers have described a role for ultraconserved RNAs (uc).73+A and uc.338+ as oncogenes in colorectal cancer samples [4], whereas other groups identified uc.388+ as an oncogene in hepatocellular carcinoma tissues [5]. Recently, researchers found uc.283+ to be highly specific for pluripotent stem cells and highly expressed in cases of glioma, one of the most untreatable cancers [6].

While microRNAs (miR) and other types of non-coding RNAs, such as metastasis-associated lung adenocarcinoma transcript 1 (*MALAT1*), imprinted maternally expressed transcript (H19), and long intergenic noncoding RNA Up-regulated in bladder cancer 1 (linc-UBC1), greatly contribute to the biological function of bladder cancer (BlCa) and are being increasingly explored to improve the clinical care of patients, the biological importance of these UCRs in the pathogenesis of BlCa has not yet been investigated, and their function is currently unknown. These elements are more highly conserved than protein coding regions, suggesting that these segments have important, if not vital, biological functions.

We present here a comprehensive genomic examination of the transcriptional status of UCRs in a large panel of human BlCa samples. T-UCRs exert their biological function by sequence complementarity to other RNAs species. We proposed and validated a model in which uc.8+ acts as an efficient decoy for miR-596 and plays an important regulatory role in BlCa tumorigenesis.

## RESULTS

### Identification of differentially expressed T-UCRs in BlCa and normal bladder tissues

To identify the differential expression of T-UCRs in BlCa and normal bladder epithelium (NBE) tissues, we evaluated expression data on 962 sense and antisense transcripts of 481 known UCRs using a custom microarray previously used to examine ultraconserved genome expression profiles in patients with leukemia, colon cancer, or hepatocellular cancer [2, 5, 7].

**Figure 1:**
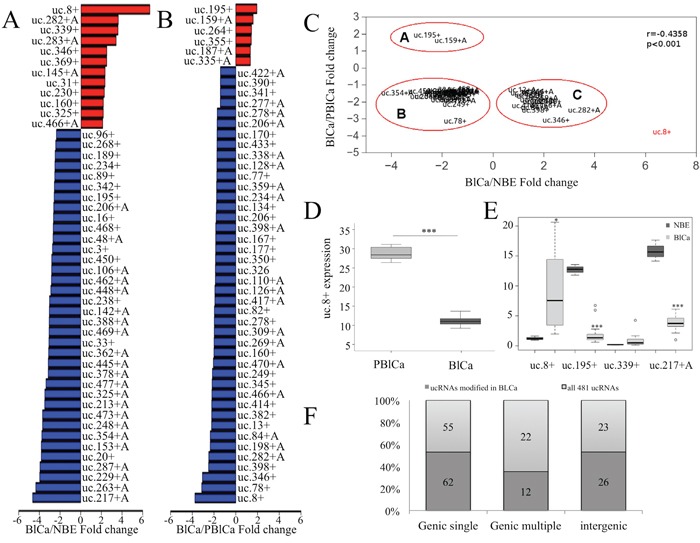
Transcribed ultraconserved region (T-UCR) expression in human bladder cancer (BlCa) tissues **A.** Bar plot of the expression of a subset of the investigated T-UCRs (48 of 293) with expression increases greater than 2 fold and expression decreases lower than −2.3 fold in BlCa and normal bladder epithelium (NBE) tissues. **B.** Bar plot of the expression of a subset of the investigated T-UCRs (48 of 141) with expression increases greater than 1 fold and expression decreases lower than −1.66 fold in BlCa and pericancerous BlCa (PBlCa) tissues.**C.** Comparison of the fold change in expression of 50 T-UCRs for which two different controls (NBE and PBlCa tissues) were used. The outlying ultraconserved RNA (uc).8+ is shown in red. **D.** RNA was extracted from 18 BlCa and adjacent PBlCa tissues. Evaluation of uc.8+ expression was assessed by quantitative real-time polymerase chain reaction (qRT-PCR). The expression of uc.8+ is higher in PBlCa than in BlCa tissues. ***P<0.001. **E.** Box plot of the fold change in uc.8+, uc.195+, uc.339+, and uc.217+A expression in BlCa and NBE samples according to qRT-PCR analysis of at least three biological repeats (subset of 22 BlCa patients and 10 NBE; Table 1, dataset 4). The bold lines inside the boxes in panels D and E represent the medians. The boxes represent the first (Q1) and the third (Q3) quartiles, and the two whiskers represent the minimum and the maximum values, except for outliers. Circles represent outliers, i.e., values lower than Q1-1.5 (Q3-Q1) or higher than Q3+1.5 (Q3-Q1). P values were obtained using the Mann-Whitney U test. ***P<0.001. **F.** T-UCR classification with respect to the transcripts as single, multiple, or intergenic is depicted for all T-UCRs and for the group of T-UCRs that are deregulated in BlCa tissues. Selective enrichment of a specific group of T-UCRs was not observed in BlCa tissues. Source data for this figure are available online. Abbreviations: ucRNA, ultraconserved RNA; T-UCR, transcribed ultraconserved region; qRT-PCR, quantitative real-time polymerase chain reaction.

We first compared the ultraconserved genome profiles for 24 BlCa patient samples (1_BlCa) and 17 NBE samples (1_NBE; clinical characteristics in Table 1, dataset 1). Analysis of microarrays for two-class unpaired data comparison [8] identified 293 T-UCRs (∼60% of all T-UCRs analyzed) that were differentially expressed at a statistically significant level (P<0.05, q<0.025) in 1_BlCa and 1_NBE (Supplementary Table S1, top-ranked T-UCRs). Of these, the expression of 75 of them increased by 1.1 to 6.7 fold in the BlCa samples; whereas the expression of 218 of them decreased by 0.9 to 0.2 fold in BlCa tissues. Compared with the NBE samples, uc.8+ expression increased the most (6.6 fold; P = 0.001), and uc.217+A expression decreased the most (5.0 fold) in BlCa samples (Figure 1A).

**Table 1:** Clinical characteristics of patients with bladder cancer (BlCa)*

Features	Dataset 1		Dataset 2		Dataset 3		Dataset 4		Dataset 5
	1_BlCa (N=24)	1_NBE (N=17)	2_BlCa (N=3)	2_PBlCa (N=3)	3_BlCa (N=18)	3_PBlCa (N=18)	1_BlCa (N=40)	1_NBE (N=16)	1_BlCa (N=18)
**Age**(mean±SD)	64.5±13.8	61.9±7.1	60.6±8.0	60.6±8.0	63.0±15.7	63.0±15.7	68.9±10.3	65.6±3.4	72.1±12.4
**Sex (male/female)**	19/5	17/0	3/0	3/0	16/2	16/2	27/13	16/0	12/6
**Clinical stage**									
**Ta**–**T0**	12	/	/	/	5	/	9	/	3
**T1**	3	/	1	/	4	/	10	/	3
**T2**	3	/	1	/	4	/	7	/	3
**T3–T4**	6	/	1	/	5	/	14	/	9
**Pathological grade**									
**G1**	4	/	/	/	6	/	12	/	6
**G2**	8	/	2	/	4	/	8	/	6
**G3**	12	/	1	/	8	/	20	/	6

* The median follow-up duration for these patients was 27.82 months.

All patients were classified according to the 1997 UICC TNM classification for the stage and OMS 2004 for the grade.

Abbreviations: SD, standard deviation; NBE, normal bladder epithelium; PBlCa, pericancerous BlCa; ISH, *in situ* hybridization.

Because researchers previously showed that histological samples of apparently NBE obtained from BlCa patients exhibited genetic alterations [9], we compared the ultraconserved genome profiles of BlCa samples collected from three patients and matched pericancerous BlCa (PBlCa) tissues (urothelium surrounding the tumors) obtained from the same patients (clinical characteristics are shown in Table 1, dataset 2). We identified 141 T-UCRs that were differentially expressed (Supplementary Table S2). Compared with the PBlCa samples, in BlCa samples, the expression of six of these T-UCRs increased by 1.3 to 1.9 fold, whereas the expression of 135 decreased by 0.8 to 0.2 fold. uc.195+ was the most upregulated and uc.8+ was the most downregulated in BlCa compared with PBlCa (Figure 1B). We merged the data of differentially expressed T-UCRs obtained from these two comparisons (1_BlCa/1_NBE and 2_BlCa/2_PBlCa) and identified 50 T-UCRs for which the change in expression was concordant (Figure 1C).

For these T-UCRs, we correlated the magnitude of the fold change in the two comparisons. We observed good overall correspondence in the fold increase of T-UCR expression (r=−0.4358, P<0.001). Few outliers drove this trend, with the most extreme being uc.8+ (6.6 fold increase when comparing BlCa with NBE versus 0.3 fold decrease when comparing BlCa with PBlCa).

To understand the regulation of T-UCRs during BlCa progression, we compared T-UCR expression in BlCa, PBlCa, and NBE tissue samples using data from the microarray. We found that some T-UCRs were differently expressed in these three tissue types (Supplementary Figure S1A and S1B). Particularly, expression of uc.8+, uc.78+, uc.249+, uc.282+, and uc.339+ was markedly higher in PBlCa than in NBE but was markedly lower in BlCa than in PBlCa tissues (Supplementary Figure S1A). Nevertheless, the expression of these T-UCRs in PBlCa tissues seemed to be higher than in NBE, and this difference was statistically significant only for uc.8+ and uc.339+ (P<0.001). Nevertheless, a large population may achieve statistical significance for the difference in expression of the other deregulated T-UCRs shown in Supplementary Figure S1A and S1B.

uc.8+ was the most upregulated ultraconserved element in PBlCa tissue samples (Figure 1D) when compared with corresponding BlCa tissue samples (3_BlCa and 3_PBlCa) obtained from 18 patients (clinical characteristics are shown in Table 1, dataset 3). These findings suggest that BlCa and PBlCa tissues have different UCR transcription patterns.

### Validation of differentially expressed T-UCRs

To validate the results obtained by microarray analysis, we assayed the expression of four ultraconserved RNAs (uc.8+, uc.195+, uc.339+, and uc.217+A), by quantitative real-time polymerase chain reaction (qRT-PCR) in a subset of 22 patients and 10 normal controls randomly selected from dataset 4 (clinical characteristics are shown in Table 1). The results showed an increase of 6.5 fold for uc.8+, an increase of 2.45 fold for uc.339+, a decrease of 0.105 fold for uc.195+, and a decrease of 0.25 fold for uc.217+A in BlCa tissues compared with NBE (Figure 1E). These results are consistent with the trend shown by microarray analysis results and indicate the primary role that uc.8+ may have in BlCa onset and progression.

### Genomic features of the investigated T-UCRs

We investigated the genomic properties of the 293 T-UCRs identified by the microarray analysis and determined their transcript localization. Since a gene may have multiple slightly different transcripts, the same T-UCR can have multiple localizations, i.e., the T-UCR might be exonic in one transcript and intronic in another. We denoted this possibility as multiple T-UCRs mapping to different regions in different transcripts to distinguish it from cases in which the T-UCR maps to a single transcript or to the same position in all transcripts (Supplementary Figure S2A and S2B and Supplementary Table S3). Of the 293 T-UCRs considered in this study, we found that most were single (62%), some were intergenic (26%), and a few had multiple transcripts (12%). uc.285+ is an example of T-UCR that maps to multiple transcripts of CCAR1 gene (Figure S2B). None of these categories was selectively enriched in BlCa tissues, and the proportion was similar to those of all T-UCRs (Figure 1F).

### Increased uc.8+ expression in BlCa tissues

**Figure 2:**
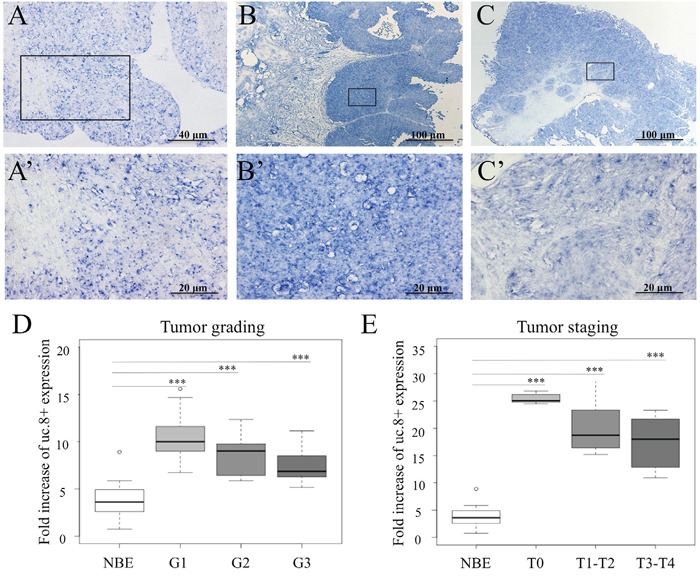
Ultraconserved RNA (uc). 8+ expression in human bladder cancer (BlCa) tissues Representative images show the expression of uc.8+ by *in situ* hybridization in **A.** normal bladder epithelium (NBE), **B.** low-grade BlCa tissues, and **C.** high-grade BlCa tissues. **A'**, **B'**, and **C'** represent enlargement of specific areas. **D.** uc.8+ expression evaluated by qRT-PCR was stratified according to grade of BlCa (n=40; Table 1, dataset 4). **E.** uc.8+ expression evaluated by qRT-PCR in BlCa tissues (n=40) was stratified according to stage. The bold lines inside the boxes in panels D and E represent the medians. The boxes represent the first (Q1) and the third (Q3) quartiles, and the two whiskers represent the minimum and the maximum values, except for outliers. Circles represent outliers, i.e., values lower than Q1-1.5 (Q3-Q1) or higher than Q3+1.5 (Q3-Q1). P values were obtained using the Mann-Whitney U test. ***P<0.001.

To gain insight into the *in vivo* role of uc.8+, we studied its expression profile in BlCa tissues by *in situ* hybridization experiments (Figure 2A, 2B, and 2C). We used a digoxigenin-labeled RNA antisense uc.8+ probe and analyzed 18 BlCa samples (Table 1, dataset 5). We detected uc.8+ expression in most of the samples (15/18): in seven out of nine high-grade tumors and in eight out of nine low-grade tumors. Positive spots were observed mainly in the nucleus in both adjacent normal tissues and high-grade BlCa, whereas in low-grade BlCa spot signals were mainly delocalized in the cytoplasm (Figure 2A', 2B', and 2C'). Importantly, the shuttling of uc.8+ from the nucleus to the cytoplasm could suggest the interaction of uc.8+ with other cytoplasmic molecules.

Next, we looked at uc.8+ expression in 40 BlCa patients (Table 1, dataset 4). We confirmed that the expression of uc.8+ is upregulated in BlCa, but uc.8+ expression tended to be inversely related to BlCa grade (Figure 2D), suggesting an association between loss of tumor differentiation and low uc.8+ expression. We found the same results when we considered uc.8+ expression and BlCa stage (Figure 2E), suggesting an early alteration of uc.8+ expression in BlCa development.

### Genomic features and transcriptional regulation of uc.8+

**Figure 3:**
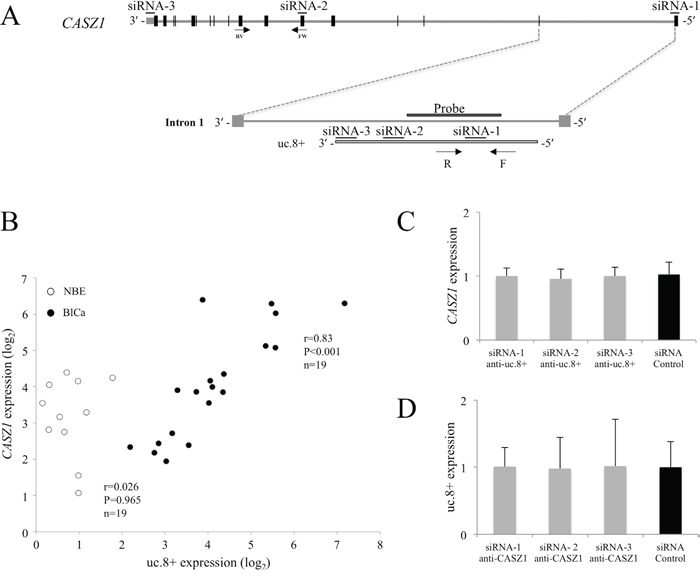
Independent regulation of ultraconserved RNA (uc). 8+ and CASZ1 in bladder cancer (BlCa) tissues **A.** Schematic representation of the intronic localization of uc.8+ within *CASZ1*. *CASZ1* exons are indicated by black boxes. The locations of the uc.8+ forward (F) and reverse (R) primers used for qRT-PCR and the probe used for *in situ* hybridization are shown. Of the siRNAs targeting *CASZ1*, siRNA-1 is located at the 5′ untranslated region (UTR), siRNA-2 is located in exon 6, and siRNA-3 is located at the 3′ UTR. **B.** RNA levels of *CASZ1* and uc.8+ were determined by qRT-PCR in BlCa (n=19, black dots) and control normal bladder epithelium (NBE) samples (n=11, empty circles). Results are presented as means ± standard deviation (SD). Spearman correlation coefficient and P values are indicated. **C.** *CASZ1* expression after silencing of uc.8+. The expression of *CASZ1* was not affected in J82 cells transfected with three different siRNAs anti-uc.8+ or siRNA control. **D.** J82 cells were transfected with siRNA anti-*CASZ1* or siRNA control. The *CASZ1* level was determined by qRT-PCR. uc.8+ expression was not affected by any of the siRNAs anti-*CASZ1* used. Data are expressed as the mean ± SD of triplicate values.

To evaluate the potential interrelationship between *CASZ1* and uc.8+ transcription, we first examined the expression of *CASZ1* by qRT-PCR for a subset of 19 patients with BlCa at different stages and 11 NBE controls randomly selected from dataset 4 (Table 1). *CASZ1*, which maps to 1p36.22, is a recently described zinc-finger transcription factor. *CASZ1* acts as a tumor suppressor gene, and researchers have shown that *CASZ1* is downregulated in high-risk phenotypes of neuroblastoma [10]. The primers used spanned a genomic region in *CASZ1* exon 6–8, which was distant from the uc.8+ region (Figure 3A). The results indicated a positive correlation between the expression of the host gene *CASZ1* and uc.8+ (r=0.83, P<0.001; Figure 3B). The *CASZ1*/uc.8+ ratio in NBE controls highly favored *CASZ1* expression, whereas in cancer patients, the ratio shifted in favor of uc.8+ expression in the different stages of BlCa (Supplementary Figure S3).

We next evaluated the uc.8+/*CASZ1* expression ratio in BlCa cell line J82, which had higher expression of uc.8+ than BlCa cell line RT112 (Supplementary Figure S4). We compared uc.8+ knockdown in J82 cells transfected with three different small interfering RNAs (siRNAs) against uc.8+ and a siRNA control (Supplementary Figure S5A) and found that siRNA-3 anti-uc.8+, at a final concentration of 200 nM, was the most effective.

The oligonucleotides used to silence the expression of uc.8+ had no effect on the expression of the host gene *CASZ1* (Figure 3C). Furthermore, uc.8+ expression was unchanged in siRNA-2 anti-*CASZ1*–transfected J82 cells (Figure 3D), despite a 78% of reduction in *CASZ1* expression (Supplementary Figure S5B). These observations confirmed that uc.8+ was not expressed as part of *CASZ1* gene.

**Figure 4:**
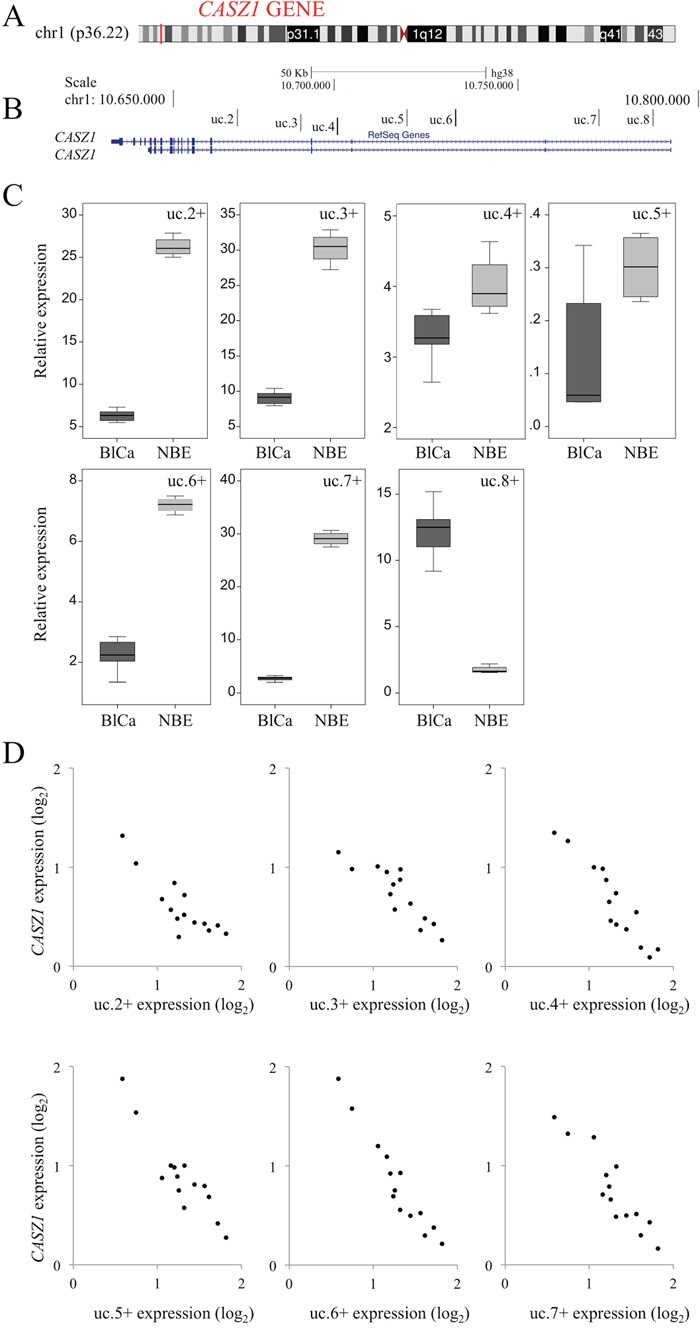
Correlation of transcribed ultraconserved regions (T-UCRs) with the host gene CASZ1 in bladder cancer (BlCa) **A.** Representation of the genomic localization of *CASZ1* with respect to 1p36.22 (red) obtained using the UCSC Genome Browser (University of California Santa Cruz). **B.** Representation of the seven T-UCRs in *CASZ1* according to their genomic locations with respect to protein-coding genes (*CASZ1* defined using the RefSeq database). **C.** The expression levels for all T-UCRs located in *CASZ1* were measured using qRT-PCR. RNA was extracted from BlCa tissues obtained from 24 patients (dark gray) and 17 normal bladder epithelium (NBE) samples (gray) (Table 1, Dataset 1). The bold lines inside the boxes represent the medians. The boxes represent the first (Q1) and the third (Q3) quartiles, and the two whiskers represent the minimum and the maximum values, except for outliers. Circles represent outliers, i.e., values lower than Q1-1.5 (Q3-Q1) or higher than Q3+1.5 (Q3-Q1). **D.** Representative negative correlation of *CASZ1* and T-UCR (uc.2+–uc.7+) expression in patients with BlCa (n=14). qRT-PCR analysis results of *CASZ1* expression (abscissa) versus T-UCR expression are shown.

In humans, we observed that uc.8+ is located within intron 1 of *CASZ1* near six other T-UCRs: uc.2+ and uc.3+ are located within intron 4, uc.4+ is located within intron 3, uc.5+ and uc.6+ are located within intron 2, and uc.7+ is located within intron 1 of the main transcript identified as *CASZ1* (Figure 4A and 4B). The six T-UCRs localized in *CASZ1* exhibited very low levels in BlCa compared with NBE samples (Figure 4C). Accordingly, we observed a negative correlation between the expression of all the T-UCRs contained in *CASZ1* and *CASZ1* (Figure 4D) in BlCa patients, supporting a role for uc.8+ in BlCa progression. These findings exclude the basic hypothesis that uc.8+ works as an antisense controller of *CASZ1* and indicate that the expression of uc.8+ is independent of the expression of *CASZ1*.

### Identification of the full-length transcript encoding uc.8+

**Figure 5:**
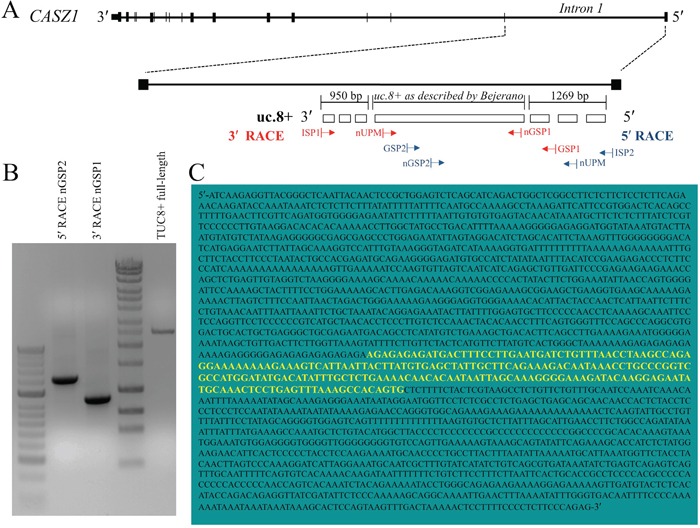
Features of intronic location of ultraconserved RNA (uc). 8+ in CASZ1 **A.** Schematic representation of the transcript including uc.8+ with respect to *CASZ1*. J82 RNA was retrotranscribed by using the SMARTer Rapid Amplification of cDNA Ends (RACE) cDNA Amplification kit (Clontech). Primers used for the 5′ RACE were as follows: Universal Primer Mix (UPM) that recognized the SMARTer oligonucleotide added at the 5′ end, gene specific primers 1 (GSP1) that recognized the sense transcript, and GSP2 primers that recognized the antisense transcript. The arrows represent the direction of amplification from the gene-specific primers that successfully amplified the unknown regions of the *TUC8* gene. **B.** 5′- and 3′-RACE polymerase chain reaction (PCR) performed to amplify the uc.8+ cDNA. **C.** Sequence of the complete uc.8+ transcript (2435 bases) as determined using RACE. The yellow sequence was reported by Bejerano *et al*, 2004 [v1].

We further characterized the uc.8+ transcript to understand its relevance observed in BlCa. We cloned the full-length uc.8+ transcript using rapid amplification of cDNA ends (RACE) and designated the DNA sequence as TUC8. The genomic location of TUC8 is shown in Figure 5A. J82 RNA was retrotranscribed with the SMARTScribe Reverse Transcriptase to generate a complete cDNA copy of the original RNA with an additional SMARTer sequence at the end that served as a template for the reverse transcription. To study TUC8, we used intronic primers that would not recognize the *CASZ1* coding sequence (Figure 5A). No products were produced with the antisense intronic primer, suggesting that TUC8 is not encoded in antisense. PCR with the intronic sense primer produced a single defined band of ∼1269 nt that was further sequenced, leading to the characterization of the 5′ end of TUC8 (Supplementary Figure S6A). As shown in Figure 5B and Supplementary Figure S6B, 3′ RACE studies identified 950 nt at the 3′ end downstream from the uc.8+ sequence identified by Bejerano *et al* [1]. The TUC8 gene has a total of 2435 bases, including the 216-nt ultraconserved (uc.8+) sequence from position −950 to position +1269 upstream from the 3′-untranslated region (Figure 5C).

We used the Open Reading Frame Finder in the Sequence Manipulation Suite [11] to scan TUC8 for open reading frames at least three codons long that could begin with any codon and be located in any possible frame on both direct and reverse strands according to the standard genetic code. We identified eight possible open reading frames starting with an ATG codon, ending with a stop codon, and comprising 3 to 34 codons (Supplementary Table S4). We scanned the full-length uc.8+ transcript sequence using the PROSITE protein database [12], excluded motifs with a high probability of occurrence, and found no protein domains or relevant functional sites within uc.8+ sequence. We concluded that uc.8+ is part of TUC8, which is transcribed independently on *CASZ1*.

### Uc.8+ and BlCa cell proliferation, migration, and invasion

**Figure 6:**
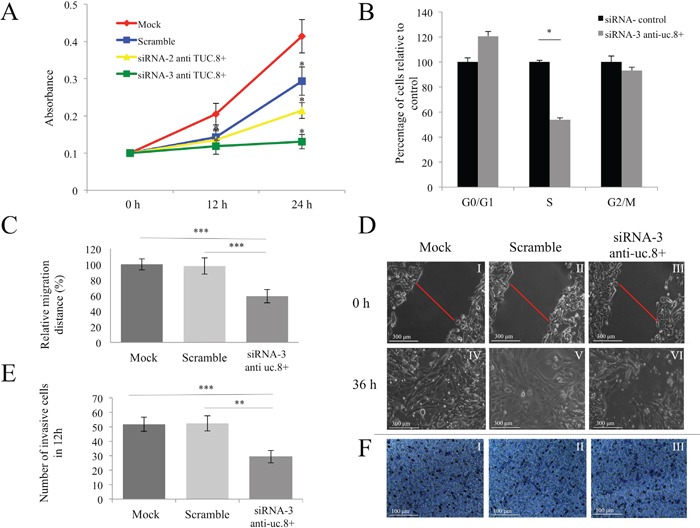
Effect of ultraconserved RNA (uc). 8+ silencing on bladder cancer (BlCa) cell proliferation, migration, and invasion **A.** J82 cells were transfected with siRNA anti-uc.8+ or siRNA control and were seeded in 96-well plates. Cell proliferation was determined at the indicated time points. The number of cells per well was measured by the absorbance at 595 nm. The results show data from at least three independent experiments. Cell growth after transfection with siRNA-3 anti-uc.8+ was not significantly different from that of cells transfected with siRNA-2 anti-uc.8+. P values were obtained using the Student t test for independent samples. *P<0.05. **B.** J82 cells were transfected with siRNA-3 anti-uc.8+ or siRNA control for 72 h, and analysis of cell-cycle distribution was performed by flow cytofluorometry. Bars represent the means and standard deviation (SD) of three experiments. P values were obtained using the Student t test for independent samples. *P<0.05. **C.** J82 cells were transfected with siRNA-3 anti-uc.8+ or siRNA control. After 24 h, a single scratch was made in the urothelial monolayer. Cell migration was quantified by measuring the distance between the invading front of the cells in three randomly selected microscopic fields (magnification 20x) for each condition and time point. The degree of motility is expressed as the percentage of wound closure compared with the zero time point. Data are representative of three experiments and are expressed as means ± SD. P values were obtained using the Student t test for independent samples. **P<0.01 and ***P<0.001. **D.** Representative views of wound healing assay was captured and recorded at 0 and 36 h demonstrating reduced migration of J82 cells after uc.8+ silencing. The scale bar in the image is 300 μm. Magnification, 100× (panel I–VI). **E.** The number of invasive J82 cells in mock, scrambled siRNA, and siRNA-3 anti-uc.8+ groups, which were significantly higher than those in the siRNA-3 anti-uc.8+–transfected group. Data are representative of three experiments and are expressed as means ± SD. P values were obtained using the Student t test for independent samples. **P<0.01 and ***P<0.001. **F.** Representative microscopic images (magnification, 20×) with crystal violet staining of migrated J82 cells. Mock, scrambled siRNA, and siRNA-3 anti-uc.8+ transfected cells are shown (panel I to III).

To determine the role of endogenous uc.8+ expression in BlCa, we examined the effect of uc.8+ silencing on J82 cell proliferation, migration, and invasion. As shown in Figure 6A, after 24 hours, growth was greatly reduced, by 55.46% ± 1.30% (mean ± standard deviation [SD]) in siRNA-3 anti-uc.8+–transfected cells and by 26.84% ± 1.10% (mean ± SD) in siRNA-2 anti-uc.8+–transfected cells, when compared with control siRNA–transfected cells. We next used cytofluorometry to assess the effects of uc.8+ silencing on J82 cell-cycle distribution. Compared with the control, siRNA-3 anti-uc.8+–transfected samples had 45% fewer cells in S phase (P<0.05) and 7% fewer cells in G2/M phase (Figure 6B).

We performed an *in vitro* wound-healing assay to measure cell migration, 36 h after silencing uc.8+ (Figure 6C). Imaging of cell migration revealed that uc.8+ silencing impaired the motility of J82 cells *in vitro* by about 40% when compared with control siRNA–transfected cells (Figure 6D). As shown in Figure 6E and 6F, uc.8+ silencing markedly inhibited cellular invasion when compared with corresponding control cells in a transwell invasion assay. Taken together, these results suggest that uc.8+ silencing suppressed the ability of BlCa cells to proliferate, migrate, and invade *in vitro*.

### Correlation between uc.8+ and miR-596 expression in BlCa

Since many long non-coding RNAs function as transcription regulators in RNA-RNA and RNA-DNA interactions via simple one-to-one base pairing [2, 3], we hypothesized that T-UCRs may exert their function by regulating other RNAs. To explore this possible function of UCR-encoded transcripts, we used the RNAhybrid target prediction software program [13] to identify putative miR target sites in all T-UCRs described by Bejerano *et al* [1]. By using highly stringent conditions to predict miR binding sites, we identified only two miR-binding sites—for miR-596 (Supplementary Figure S7A) and miR-381-3p (Supplementary Figure S7B)—on the uc.8+ sequence.

We focused on the uc.8+::miR-596 interaction for two reasons. First, the RNA duplex uc.8+::miR-596 has a high probability to form *in vivo* with a low minimum free energy (MFE) value of −33.5 kcal/mol and a statistically significant P value of 0.001926. With this small P value, it is unlikely that the MFE is the result of a random complementarity between miR and the target, and biological relevance can be assumed. Second, in a comparison of the predicted uc.8+ secondary structure (Supplementary Figure S7C and S7D) with the entire RNA sequence of 2435 bases transcribed *in vitro* (RACE analysis, Figure 5C), we observed that the uc.8+ sequence reported by Bejerano *et al* [1] folds in the same manner as the full-length transcript we found. The three-dimensional structure of uc.8+ remains sufficiently accessible, with a MFE value of only −3.90 kcal/mol, to bind to miR-596 (Supplementary Figure S7E). With a fairly good MFE value of −27.1 kcal/mol and a P value of 0.025596, the predicted RNA duplex uc.8+::miR-381-3p also has a high probability of forming *in vivo*. We found that the binding site for miR-596 on uc.8+ is localized in a region that is structurally available to binding, showing 18 out of the 23 bases involved in the interaction with miR-596. However, the secondary structure of uc.8+ suggests that it is more likely to form an RNA duplex with miR-596.

**Figure 7:**
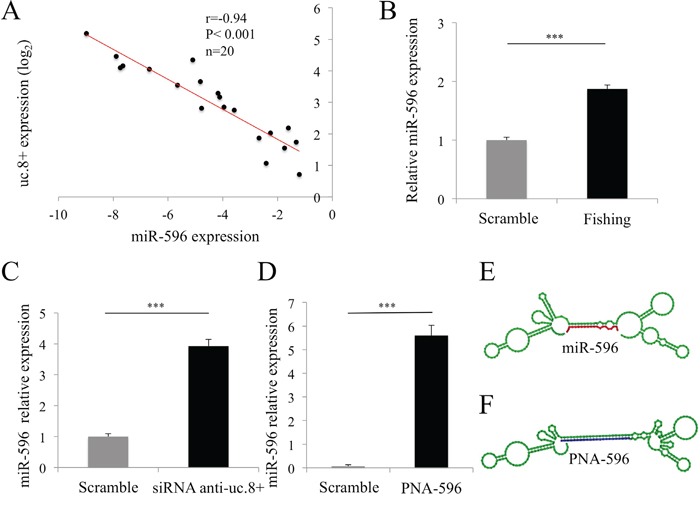
Ultraconserved RNA (uc). 8+ and microRNA (miR)-596 interaction and target regulation in bladder cancer (BlCa) cells **A.** Representative positive correlation between the expression of uc.8+ and that of miR-596 in BlCa samples from 20 patients (Table 1, dataset 4) measured using qRT-PCR. Correlation was computed using the Spearman correlation coefficient. **B.** Expression of miR-596 in J82 cell extracts after retrieval of endogenous uc.8+ with a peptide nucleic acid (PNA)/uc.8+ probe. Data are expressed as the means ± standard deviation (SD) of triplicate values. P values were obtained using the Student t test for independent samples. ***P<0.001. **C.** Relative expression of miR-596 in siRNA anti-uc.8+–transfected J82 cells. Data are expressed as the means ± SD of triplicate values. P values were obtained using the Student t test for independent samples. ***P<0.001. **D.** J82 cells were transfected with a PNA mimic of miR-596 (PNA-596). Minimum free energy = −51.10 kcal/mol. Data are expressed as the means ± SD of triplicate values. P values were obtained using the Student t test for independent samples. ***P<0.001. **E.** Predicted co-folded secondary structure of uc.8+ (green sequence) bound to miR-596 (in red), according to the RNAfold browser. **F.** The blue sequence shows that PNA-596 is perfectly complementary to uc.8+.

To determine the biological significance of the predicted uc.8+::miR-596 RNA duplex, we used qRT-PCR to investigate the correlation between uc.8+ and miR-596 expression in a subset of 20 BlCa tissue samples (Table 1, dataset 4). The BlCa samples had consistently lower miR-596 expression than the control; miR-596 expression inversely and significantly correlated with uc.8+ expression (r=−0.94, P<0.001; Figure 7A).

### Identification of uc.8+::miR-596 interaction using a fishing/competition approach

We used a fishing/competition approach to directly validate the interaction between uc.8+ and miR-596. We identified a single-strand region of uc.8+ to design an antisense complementary probe to capture endogenously expressed uc.8+. We used the RNAfold web server (version 2.1.5, Vienna RNA Package) to identify the uc.8+ secondary structure (216 bp) and sequences in a single strand, which formed loops even after miR-596 binding.

A key to our approach was to use biotinylated peptide nucleic acid (PNA) oligomers as a probe for fishing for uc.8+. The PNA sequence is complementary to the predicted single-strand region of uc.8+ (highlighted in blue in Supplementary Figure S7C and S7D). We chose the PNA oligomer instead of standard synthetic oligonucleotides because of its ability to bind complementary RNAs with high affinity and specificity independently from the ionic strength of the buffer (see Materials and Methods). We designed and tested probes PNA1 and PNA2, which are perfectly complementary to the uc.8+ sequence. We verified the ability of these PNA probes to capture uc.8+ in a sequence-specific manner by incubating the probes with total RNA extracts, immobilizing the endogenous hybrids PNA::uc.8+ on streptavidin beads, and amplifying uc.8+ using qRT-PCR. We found that PNA1 captured a large amount of uc.8+, so we chose PNA1 for the fishing approach (Supplementary Table S5, Supplementary Figure S7C and S7E [light blue], and Supplementary Figure S8). We used a scrambled PNA probe with no sequence homology with any human RNAs (input reads) as a negative control. Finally, we used streptavidin beads to isolate target RNAs (uc.8+ and its interactors) from J82 cells. RT-PCR analysis results for candidate miR demonstrated a marked enrichment of miR-596 compared with the input reads (Figure 7B). Furthermore, uc.8+ knockdown resulted in a concomitant increase in miR-596 expression by about four fold in J82 cells (P<0.001; Figure 7C), supporting a biological correlation between the two molecules.

To further demonstrate that miR-596 binds to uc.8+, we set up a competition assay. We transfected J82 cells with a PNA mimic of miR-596 (PNA-596; final concentration, 200 nM; Figure 7D). In contrast to the partial complementarity observed with miR target interactions, the PNA-596 was designed to be perfectly complementary to the uc.8+ sequence. PNA-596 competes only with endogenous miR-596 for binding to uc.8+ and efficiently displaces miR-596 in a sequence-specific manner (Figure 7E and 7F). After 48 h, levels of miR-596 were analyzed by RT-PCR. The complex formed by PNA-596/DNA (oligonucleotide primers used in the PCR) blocked the formation of a PCR product [14] and allowed the selective amplification of endogenous miR-596. Expression of miR-596 was significantly increased, by 77% (P<0.001), in PNA-596–transfected J82 cells compared with the control (Figure 7D), confirming the binding of miR-596 to uc.8+.

### Cellular localization of uc.8+ by light-up PNA probes

**Figure 8:**
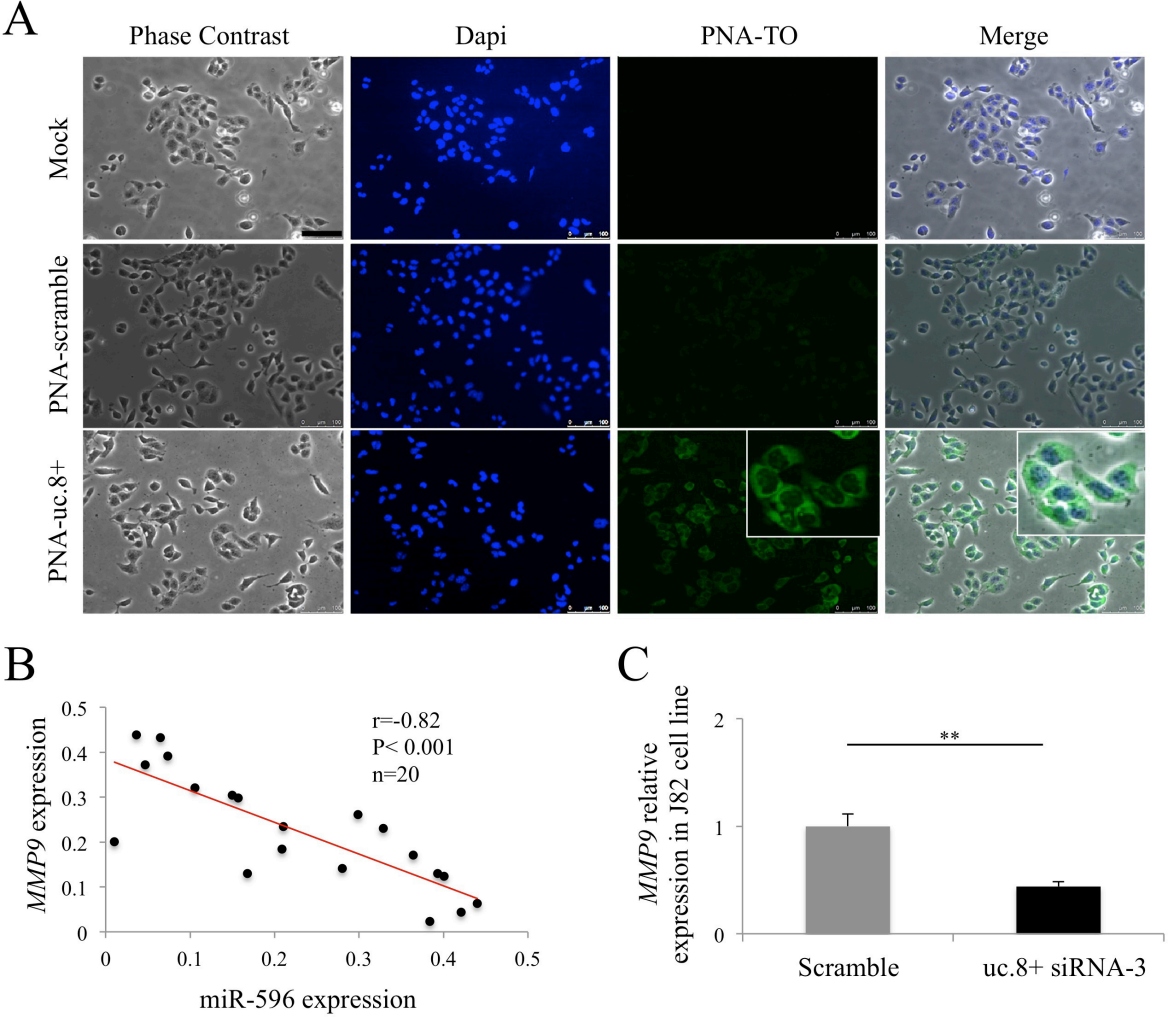
Cellular localization of ultraconserved RNA (uc). 8+ **A.** Images acquired using inverted fluorescence microscope (magnification, 20×) of J82 control cells (Mock) after transfection with PNA-TO scramble-R8 (PNA-scramble) or with TO-PNA1-R8, the PNA complementary to uc.8+ (PNA-uc.8+). Images were recorded with excitation wavelength (lex)=450–490 nm (DAPI) or lex=510–540 nm (PNA-TO); the superimposition of the images recorded is also reported (Merge). All images were taken with the same confocal microscopy settings. Scale bar, 100 mm. Nuclei of J82 cells were stained with DAPI (blue). **B.** Inverse correlation between the expression of microRNA (miR)-596 and *MMP9* in BlCa samples from 20 patients measured using qRT-PCR. **C.** Relative expression of *MMP9* in siRNA-3 anti-uc.8+ –transfected J82 cells. Endogenous uc.8+ levels in the control cells are shown in grey. Data are expressed as the means ± standard deviation of triplicate values. P values were obtained using the Student t test for independent samples. **P<0.01.

The interaction between uc.8+ and miR-596 suggests that T-UCRs are located in the cytoplasm because miR interaction occurs preferentially in this subcellular compartment. To confirm our previous *in situ* hybridization finding, which indicated that uc.8+ is preferentially located in the cytoplasm of BlCa cells, we employed the PNA1 sequence (PNA-uc.8+) further modified by a polyarginine at the C-terminus and by the fluorescent probe thiazole orange (TO) at the N-terminus. The polyarginine sequence at a final concentration of 4 μM has been demonstrated to efficiently carry PNAs into the cytoplasm of many cells [15]. TO is defined as a light-up probe because its fluorescence strongly increases after hybridization of the TO-modified oligomer to a complementary RNA sequence. As shown in Figure 8A, only a light background was evident in the cytoplasm of J82 cells transfected with the PNA scramble sequence, whereas cells transfected with PNA-uc.8+ showed increased fluorescence. This increased fluorescence indicates the presence of uc.8+ in the cytoplasm and suggests that the uc.8+::miR-596 complex is also located in the cytoplasm.

### Effect of uc.8+ silencing on BlCa progression

On the basis of our findings of the phenotypic consequences of uc.8+ downregulation, we investigated the effect of uc.8+::miR-596 complex formation on the target of miR-596 and on BlCa formation. Because Endo *et al* [16] demonstrated that matrix metallopeptidase 9 (*MMP9*) is modulated by miR-596 in oral squamous cell carcinoma cell lines, we hypothesized that uc.8+ may regulate the expression of *MMP9*, a mesenchymal marker that is overexpressed in muscle-invasive BlCa cells, via binding to miR-596. We observed an inverse correlation between the expression of miR-596 and that of its putative target *MMP9* in BlCa tissues (Figure 8B), suggesting that uc.8+ acts as a decoy for miR-596, preventing the binding to the miR targets. To confirm these observations, we evaluated the effect of uc.8+ silencing on *MMP9* expression and observed a decrease in *MMP9* expression of about 50% compared with the control (P<0.01) (Figure 8C). These results suggest that miR-596 has a tumor-suppressive effect in BlCa and that uc.8+ promotes BlCa.

## DISCUSSION

We identified a new long non-coding RNA transcript containing uc.8+, the highly ultraconserved sequence described by Bejerano *et al.* [1] uc.8+ is highly conserved across several species, including rats, mice, humans, chimpanzees (99%), dogs (99%), zebrafish (91%), and fugu (86%). This extremely high conservation suggests that these T-UCRs may play essential roles in cellular biology that are similar across species.

We found that the expression of uc.8+ was highly upregulated in BlCa tissues and cell lines and that the silencing of uc.8+ expression in BlCa cells, markedly reduced cell proliferation, migration, and invasion. These findings suggest that, in normal cells, uc.8+ has a function unrelated to tumorigenesis but that its increased expression after cell transformation, promotes tumor cell growth, migration, and invasion.

Recently, researchers demonstrated that uc.283+A controls pri-miR processing [17], pointing to another layer of regulation of miR activity; to determine whether T-UCRs modulate miR activity, we investigated the miR-binding domain accessibility, as determined by base-pairing interactions within the uc.8+ predicted secondary structure, RNA binding affinity, and RNA species abundance in bladder tissues. It has been reported that RNA conformations within a single transcript were a determinant of whether the transcript was targeted by specific miRs, as target sites buried in secondary structures may sterically hinder the sequence involved in the binding with miRs. The accessibility of miR target sites can change under different biological states, indicating an additional layer of gene regulation [18]. We found that the binding site for miR-596 on uc.8+ is localized in a region that is structurally available to binding; furthermore, the uc.8+ binding site for miR-596 is fully conserved across several species' genomes and occurs in a region with the highest energy score for the predicted co-folded secondary structure of uc.8+. Moreover, the 216 ultraconserved nucleotides in the entire 2435-bp uc.8+ transcript conserved the predicted secondary structure of uc.8+ that instead changed with the addition of more artificial flanking base pairs to the real transcript sequence. Therefore, the interaction between miR-596 and uc.8+ appears to be not only bp-mediated but also structurally accessible.

Cellular location is an important determinant in understanding the functional roles of non-coding RNAs. Fluorescence microscopy results for J82 cells showed that uc.8+ and miR-596 co-existed in cytoplasm and were mutually available to interact, supporting the role of T-UCRs as regulators of gene expression through the modulation of miR activity. Furthermore, our *in situ* hybridization findings in BlCa tissues indicated that positive spots for uc.8+ occurred mainly in the nucleus in both NBE and high-grade BlCa, whereas in low-grade BlCa, the spot signals were mainly delocalized in the cytoplasm, where uc.8+ can interact with other cytoplasmic molecules in the early stages of cancer. Additionally, the expression of uc.8+ tended to be inversely related to BlCa grade and stage, suggesting that an early alteration of uc.8+ expression is involved in BlCa development.

We validated the interaction between uc.8+ and miR-596 via a fishing/competition approach with a biotinylated PNA probe. The identification of RNA complexes is usually performed via incubating cells with cross-linking fixatives such as formaldehyde or 4′-aminomethyl trioxsalen, which may impair or alter the cell environment [19, 20]. The highly specific PNA probe for uc.8+ allowed us to isolate the uc.8+::miR-596 complex in an environment that approximated the physiological condition. To confirm this interaction, we designed and produced a PNA that was perfectly complementary to uc.8+ in the region of the miR-binding site. Our results show that miR-596 interacted with uc.8+. We also foud an inverse correlation between uc.8+ and miR-596 in cancer tissues, indicating that the interaction between miR-596 and uc.8+ actually occurs *in vivo*. We found that uc.8+ acts as a decoy for miR-596, inducing the upregulation of its targets, including MMP9, which supports previous findings that miR-596 is a tumor suppressor involved in regulating MMP9 [16]. Like other proteases in the MMP family, MMP9 is involved in the degradation of collagen IV in the basement membrane and extracellular matrix and facilitates tumor progression, including invasion and metastasis. Our results help explain how T-UCRs control cellular events via base pairing. Additionally, we show that the expression of miR-596 is reduced by uc.8+, which acts like a sponge; this finding is consistent with a previous report [21]. We speculate that the miRNA-596 bound to uc.8+ may be partially degraded by a mechanism analogous to that observed for antagomirs that promote miRNAs degradation. However, the exact mechanism is still unclear, and we will focus on this topic in future research.

Clustered T-UCRs localized in the intronic region of *CASZ1* are differentially expressed in cancer cells and are members of the BlCa signature: uc.3+, uc.4+, and uc.5+ expression is downregulated and uc.8+ expression is upregulated in BlCa tissue. In a mouse model, some of the scanned conserved regions in this locus, including uc.2+, uc.5+, and uc.8+, activate flanking genes and have been associated with gene regulation [22]. Given the decades of research focused on transcriptional control from a transcription factor point of view, it is interesting to speculate about the purpose of this additional layer of regulation carried out by non-coding RNAs as suggested by the intronic localization of the seven T-UCRs in *CASZ1*. Therefore, the discovery of uc.8+ as a sponge for miRs is an example of interepigenetic regulation and also indicates the richness of the genetic regulatory machinery. These interactions between evolutionarily conserved regions of DNA suggest that natural selection has preserved this potentially regulatory layer that uses RNA to modulate miR levels, opening up the possibility for development of useful markers for early diagnosis and prognosis as well as for development of new RNA-based cancer therapies.

## MATERIALS AND METHODS

### Patient samples

Five BlCa datasets were used for this study (Table 1). Dataset 1 included samples from 24 patients and 17 NBE samples. Dataset 2 included PBlCa and BlCa samples from three patients. Each of the BlCa samples was hybridized in triplicate, but the surrounding tissues were hybridized once owing to the limited available tissue. Dataset 3 included PBlCa and BlCa samples from 18 patients. Dataset 4 included BlCa samples from 40 patients and 16 NBE samples. Dataset 5 included BlCa samples from 18 patients. The control group consisted of NBE tissue samples obtained from men who underwent retropubic prostatic adenomectomy for benign prostatic hyperplasia. Tumor samples from datasets 1 and 2 were used to determine ultraconserved genome expression profiles, whereas tumor samples from dataset 3 and 4 were used as validation set. Dataset 5 samples were used for *in situ* hybridization. Subgroups of patients were chosen from the datasets 3 and 4 randomly as independent validation sets. All patients were classified according to the 1997 UICC TNM classification for the stage and OMS 2004 for the grade. Table 1 lists the clinical characteristics of the BlCa patients. The demographic data of the groups did not differ. Patients with low-grade BlCa underwent transurethral resection, and those with high-grade BlCa received radical cystectomy. None of the patients underwent preoperative radiotherapy, chemotherapy, or other cancer treatments. The participants provided written informed consent.

All of the tissue samples were collected at a major urological center (University “Federico II,” Naples, Italy) from 2008 to 2012, and the histological samples were obtained from the Pathology Unit and at the National Cancer Institute-IRCCS “G. Pascale Foundation. All tumor samples used in this study contained more than 80% tumor cells. Each sample was evaluated by a pathologist to confirm the postoperative pathologic diagnosis, which was performed by all of the tumor samples used in this study. All postoperative pathological diagnosis were done by two independent pathologists. All samples were stored in liquid nitrogen immediately after resection and were transferred to a −80°C freezer.

### Cell lines

Human BlCa cell lines J82 (ATCC) and RT112 (European Collection of Cell Cultures) were cultured as a monolayer in Minimal Essential Medium and Roswell Park Memorial Institute medium, respectively, supplemented with 10% fetal bovine serum. All cells grew in a humidified incubator in a 5% carbon dioxide atmosphere at 37°C.

### T-UCR expression profiling

RNA was extracted from tissue samples by using TRIzol reagent (Thermo Fisher Scientific). Total RNA (5 μg) was reverse-transcribed with biotin-end-labeled random oligonucleotide primers, and cDNA was hybridized to a custom microarray (OSU-CCC 4.0, Ohio State University Comprehensive Cancer Center), which included sense and antisense probes, one corresponding to the sense genomic sequence (named “+”) and the other to the complementary sequence (named “+A”) for all 481 human ultraconserved sequences reported by Bejerano *et al* [1]. Each probe was spotted in duplicate in two different slide locations, and therefore quadruplicate numerical values were available for analysis [2, 23]. Biotin-containing transcripts of all T-UCRs were detected in BlCa using an AlexaFluor 647 streptavidin conjugate (Thermo Fisher Scientific). Samples were scanned and analyzed using an Axon GenePix 4000B microarray scanner and GenePix software, version 6.0 (Axon Instruments). The mean fluorescence intensity of replicate spots of each probe on the microarray was subtracted from the background and was normalized using the global median method. T-UCRs present in all three replicates of each sample were selected for analysis. Differentially expressed T-UCRs were identified using class comparison analysis with BRB-ArrayTools software (version 3.6.0; Biometric Research Program, National Cancer Institute) [24]. The criterion for including a gene in the gene list was a P value less than 0.05. Normalized and raw T-UCR data files were uploaded to the Gene Expression Omnibus under accession number GSE68594.

### Reannotation of T-UCRs

All T-UCRs were reannotated using the current version of the reference genome (GRCh38/hg38), and their relationship with genomic annotations (ENCODE, Stanford University; GENCODE, Wellcome Trust Sanger Institute) was established.

### RNA extraction, reverse transcription, and qRT-PCR

Total RNA was extracted from BlCa and NBE samples and J82 and RT112 cells using TRIzol reagent. The concentration of RNA was determined by 260/280 nm absorbance using a NanoDrop ND-1000 spectrophotometer (Thermo Scientific), and the integrity of RNA was checked using gel electrophoresis. Total RNA (1 μg) was reverse-transcribed using an iScript Select cDNA Synthesis kit (Bio-Rad). qRT-PCR was performed using strand-specific primers for UCR analysis and random primers for *CASZ1* expression (Supplementary Table S5). A miRCURY LNA Universal RT miR PCR kit (Exiqon) was used for miR analysis according to the manufacturer's instructions. RT-PCR analysis was performed using an iQ SYBR Green Supermix (Bio-Rad) protocol with a CFX96Deep Well system Real-Time PCR Detection System (Bio-Rad) according to the manufacturer's instructions. Small nuclear RNA U6 was used as a reference for T-UCRs and miRs. Supplementary Table S5 lists primers used in this study for qRT-PCR. Each BlCa sample was analyzed in triplicate. The 2^−ΔΔct^ method was used for relative quantitation of gene expression, and results are expressed as log10 (2^−ΔΔct^).

### *In situ* RNA hybridization on paraffin sections

uc.8+ RNA antisense probe was synthesized from linearized plasmid in presence of digoxigenin-11-UTP (Roche). Tissue sections were dewaxed in xylene, rehydrated, postfixed in 4% paraformaldehyde, digested with proteinase K (10 μg/mL) for 10 min, subjected to acetylation for 15 min, prehybridized for 1 h, and then hybridized at 65°C overnight in 50% formamide; 0.25% sodium dodecyl sulfate; 10% dextran sulfate; 1× Denhardt solution; Tris HCl (pH 7.5, 10 mM); NaCl (600 mM); EDTA (1 mM); transfer RNA (200 mg/mL); and salmon sperm DNA (100 mg/mL), using a probe concentration of 0.8–1 mg/mL. Tissue sections were washed in 1× saline sodium citrate buffer (50% formamide) at 65°C for 30 min and then in 2× saline sodium citrate buffer for 20 min and in 0.2× saline sodium citrate buffer twice for 20 min each. After the washes, tissue sections were incubated overnight at 4°C with alkaline-phosphatase-coupled anti-digoxigenin antibodies (1:2000; Roche). After seven washes in maleic acid buffer containing Tween 20 for 1 h each and three washes in NTMT solution for 10 min each, tissue sections were incubated with nitro blue tetrazolium chloride and 5-bromo-4-chloro-3-indolyl-phosphate, 4-toluidine salt solution (Roche) and developed a blue color. Tissue sections were then mounted directly or after eosin staining. Stained sections were examined and photographed using a Leica MZ12 dissection microscope and a Nikon ECLIPSE Ni microscope. All the images were processed in Adobe Photoshop, version 10.0 (Adobe System Inc.).

### uc.8+ siRNA transfection of BlCa cells

J82 and RT112 cells were transfected with siRNAs using HiPerFect transfection reagent (QIAGEN). siRNAs were designed using siDirect software [25] with input of the complete uc.8+ sequence. uc.8+ gene silencing in J82 cells was studied to exclude the possibility of an off-target effect of siRNA anti-uc.8+. According to the scores of 16 siRNA candidates in the siDirect design site, we selected three sequences designated as siRNA-1, siRNA-2, and siRNA-3 (Supplementary Table S5) on the basis of their reduced capability to induce off-target effects correlated with the thermodynamic stability of the seed-target duplex (<10°C for siRNA-1 and siRNA-3 and <15° for siRNA-2).

All oligomers were synthesized using an ABI Expedite 8909 oligosynthesizer with standard protocols (1-μM scale), 5′-O-DMT-2′-O-TBDMS-RNA phosphoramidite monomers, and standard RNA synthesis reagents (Link Technologies) with Universal SynBase CPG 1000/110 solid support (CPG-OH, loading 0.04 meq g^−1^). High-performance liquid chromatographic analyses and purification of siRNAs were performed with NUCLEOGEL SAX (MACHEREY-NAGEL; 1000-8/46, eluted with a gradient from 0% to 100% B in A in 30 min, flow rate of 1 mL/min, λ = 260 nm; A: KH_2_PO_4_ [pH 7.0, 20 mM] and 20% [v/v] acetonitrile; B: KCl [1 M], KH_2_PO_4_ [pH 7.0, 20 mM], and 20% [v/v] acetonitrile) and RP-18 columns (Waters; C-18, 3.9 × 300 mm, eluted with solvent A [ammonium acetate (pH 7, 100 mM)] and solvent B [5%-60% acetonitrile] for 40 min, flow rate of 1.0 mL/min, λ = 260 nm) using a Waters 600 HPLC controller equipped with a Waters 996 photodiode array detector and Millennium software. The concentration of siRNA oligonucleotideswas estimated spectrophotometrically at 90°C using the following additive molar extinction coefficients: ε260 (L cm^−1^ mol^−1^) T=8800, A=15400, C=7200, G=11500, and U=9900 for the natural nucleobases.

RNA oligomers were analyzed using matrix-assisted laser desorption/ionization time-of-flight mass spectrometry (expressed in Da): 1 guide, mass calculated 7322, mass found 7331; 1 passenger, mass calculated 7204, mass found 7212; 2 guide, mass calculated 7081, mass found 7086; 2 passenger, mass calculated 7416, mass found 7418; 3 guide, mass calculated 7268, mass found 7275; and 3 passenger, mass calculated 7234, mass found 7244. Cells were transfected with siRNAs anti-uc.8+ or a nontargeting siRNA control at a concentration of 100 nM. Cells were collected 48 h after transfection.

### Transfection of mimic miR-596

J82 and RT112 cells (200,000 cells/well) were plated in six-well plates and incubated for about 3 h at 37°C. After incubation, cells were transfected with mimic miR-596 (QIAGEN) and AllStars Negative Control siRNA (QIAGEN) using HiPerFect transfection reagent at a final concentration of 5 nM. Cells were harvested 48 h after transfection and were subjected to RNA extraction. Reverse-transcriptase PCR and qRT-PCR analysis of miR-596 were conducted as described previously.

### RACE

To identify the 5′ and 3′ ends of the uc.8+ transcript, total RNA from J82 cells was extracted and treated with DNase I (RNase-free) endonuclease (Thermo Fisher Scientific), and the SMARTer RACE cDNA Amplification kit (Clontech) was used to generate RACE-ready cDNA according to the manufacturer's instructions. The cDNA ends were amplified with Platinum Taq DNA Polymerase High Fidelity (Thermo Fisher Scientific), and gene-specific primers (GSP1: 5′-AGAGAGAGATGACTTTCCTTG-3′; GSP2: 5′-TCCTGAGTTTAAAGCCACAGTG -3′) were used. The primer for the 5′ end was designed to keep it from overlapping with the transcript of the *CASZ1* host gene, ensuring that only the uc.8+ transcript was amplified. Furthermore, nested PCR analysis was performed with the nested universal primer provided with the kit (SMARTer RACE cDNA Amplification kit) and two nested gene-specific primers (NESTED-GSP1: 5′- GGTCGCCATGGATATGACA -3′; NESTED-GSP2: 5′-GGGGAAAGATACAAGGAGAA-3′). Placental RNA and transferrin receptor-specific primers provided with the kit were used as reaction controls.

The PCR fragments were then run on a 1.5% agarose gel, and DNA was extracted from the gel using a QIAquick Gel Extraction kit (QIAGEN) according to the manufacturer's instructions. The RACE products were then cloned into a TOPO TA pCR2.1 cloning vector (Thermo Fisher Scientific) according to the manufacturer's instructions, sequenced using the T7 and T3 primers and aligned using the UCSC Genome Browser (University of California Santa Cruz). DNA marker, 1-kb DNA ladder (Promega; catalog no. G571).

### Cell proliferation assays

Following uc.8+ silencing with siRNAs-2 and -3, BlCa cell proliferation was measured using the CellTiter 96 nonradioactive proliferation assay (Promega). J82 cells were seeded (500 cells per well) in 96-well plates and assayed 0, 12, and 24 h later according to the manufacturer's protocol.

### Cell cycle assays

J82 cells were harvested 72 h before treatment with siRNA control or siRNA-3 anti-uc.8+, fixed in 70% ethanol and stained in a solution containing 20 μg/ml of propidium iodide (Sigma Aldrich), 200 μg/ml (0.08 KU) of RNase (Serva). After 60 min, samples were analyzed by flow cytometry using a BD FACSCantoII™ cytofluorimeter (BD Biosciences). Data acquisition (10,000 events were collected for each sample) was performed by using the BD FACSDiva™ software (BD Biosciences), according to the manufacturer's instructions. Data were elaborated using the same software (BD FACSDiva™ software), according to the manufacturer's instructions and expressed as fractions of cells in the different cycle phases.

### *In vitro* wound-healing (migration) assay

A wound-healing assay was performed in siRNA-3 anti-uc.8+–transfected and untransfected cells to study cell migration with J82 cells (200,000 per well) seeded in six-well plates and incubated for about 3 h at 37°C, allowing the cells to adhere to and spread on the substrate completely. A single scratch was made in the urothelial monolayer using a micropipette tip. Subsequently, cells were washed once with phosphate-buffered saline solution and then were incubated. During the assay, cells were viewed under a Leica phase-contrast microscope with a 10× objective, and photographs of fixed positions on the wounds were taken after 0, 12, 24, and 36 h. The wound width was calculated by measuring the mean distance between the margins of the wound in randomly selected fields on the photographs. Cell migration was quantified by calculating the area of the wound at time points t0 (time of wounding), t24 (24 h after wounding), and t36 (36 h after wounding) by using the following formula: area (t0) - area (t24 or t36)/area (t0). Multiple photographs of the same spots in the wound area were then taken 12, 24, and 36 h after siRNA transfection for comparison. Four independent wound-healing assays were performed.

### Matrigel invasion assay

The invasiveness of J82 cells following uc.8+ silencing was determined using Boyden transwell assays. Polycarbonate filters with 8.0-μm pores (BD Biosciences) were coated with matrigel (40 μL) and were incubated in a humidified incubator at 37°C to allow for polymerization. The lower compartment of each chamber was filled with minimum essential medium supplemented with 10% fetal bovine serum. J82 cell groups and mock, scrambled, and siRNAs anti-uc.8+ were placed in serum-free minimum essential medium (0.2 mL) and were seeded on top of the collagen in the upper compartment of each chamber (4 × 10^4^ cells/chamber). After incubating at 37°C for 24 h, the cells on the upper surface of the filter were removed by wiping. Cells that traversed the filter were fixed with ice-cold methanol (1 mL) and were incubated for 10 min at room temperature. The methanol was aspired, and cells were stained with 1% crystal violet in 2% ethanol for 1 h in the dark. Excess crystal violet was removed by quickly merging the insert of the chamber in double-distilled water for 3–4 s several times. Excess water was drained from the side of the insert using a cotton swab, and the insert membrane was dried. We counted the cells on the lower side of the filter under a microscope (magnification 20x) by randomly choosing different views and calculating averages (five points for each transwell). Each migration condition was repeated in triplicate.

### Identification of miR-binding sites in ultraconserved RNA sequences

The T-UCR sequences described in the supplementary data reported by Bejerano *et al* [1] were converted to reverse-complementary RNA using Common Application Program Remote Interface software CAPRI, available at the bioinformatics portal of the CEINGE website. Human miR sequences with high confidence, i.e., those with a highly conserved level of identity throughout various mammalian species, were selected from the online database miRBase (release 19, University of Manchester) [26–30]. The target prediction tool RNAhybrid (version 2.1, Bielefeld University) was used to identify putative miR target sites in T-UCR sequences [13]. T-UCR::miR duplex formation was evaluated under highly stringent conditions using constraint-of-seed nucleotide matching of nucleotides 2–7 [31, 32] and a *P* value less than 0.05. The analysis focused on the 293 T-UCRs identified in microarray profiling of tissue samples obtained from BlCa patients.

### Prediction of the uc.8+ secondary structure

We used 216 ultraconserved nucleotides and the whole 2435-bp transcript of the uc.8+ sequence to predict the folding of the uc.8+ secondary structure with RNAfold software (Vienna RNA Package) [32]. Prediction of the folding of the secondary structure of the uc.8+::miR-596 duplex was carried out using RNAcofold software (version 2.1.5, Vienna RNA Package) [33].

### Magnetic labeling and isolation of biotinylated molecules (uc.8+ fishing in J82 cells)

Total RNA (300 μg) extracted from J82 cells was added to an appropriate buffer with 5′-biotinylated PNA oligomer uc.8+ (100 pmol) and was incubated overnight at 4°C with rotation. After complex formation, μMACS Streptavidin MicroBeads (100 μL, Miltenyi Biotec) were added to the mixture and incubated for 30 min at 4°C. μMACS columns were equilibrated with equilibration buffer for nucleic acids (100 μL) and were rinsed with the same buffer used for the binding reaction. Labeled complexes were applied to the top of the column matrix. Columns were washed with washing buffer (4 × 100 μL) to remove molecules with nonspecific binding. RNA labeled with biotinylated PNA oligomer was eluted with elution buffer (150 μL, μMACS Streptavidin kit 130-074-101, Miltenyi Biotec) according to the manufacturer's instructions. We used a 5′-biotinylated scrambled oligonucleotide as a control.

### PNA synthesis

HATU was purchased from InBios. Fmoc-PNA-cytosine(Bhoc)-OH, Fmoc-PNA-thymine-OH, Fmoc-PNA-guanine(Bhoc)-OH, and Fmoc-PNA-adenine(Bhoc)-OH were obtained from Link Technologies. Acetonitrile for liquid chromatography–mass spectrometry, N,N-dimethylformamide (DMF) for solid-phase synthesis, N,N-diisopropylethylamine (DIPEA), and dichloromethane were obtained from ROMIL. N-methylmorpholine and piperidine were obtained from Fluka and Biosolve, respectively. Fmoc-PAL-PEG-PS resin (0.18 mmol/g) was obtained from Applied Biosystems. All other chemicals were supplied by Sigma-Aldrich and were used without further purification.

Preparative purification of PNA oligomers was carried out using a Shimadzu LC-8A solvent delivery pump equipped with an SPD-M10AVP diode array detector. Preparative high-performance liquid chromatography was performed using a Phenomenex Jupiter 10-μm Proteo column (90 Ǻ, 250 × 10 mm) with a linear gradient of acetonitrile (0.1% trifluoroacetic acid) from 5% to 50% in water (0.1% trifluoroacetic acid) for 30 min with a flow rate of 5 mL/min^−1^. Pure products were characterized using liquid chromatography–mass spectrometry with an Agilent PL1110-6320 time-of-flight system.

### Biotinylated PNAs

PNA syntheses were carried out on a 2-μmol scale, following standard procedures [34, 35]. At the end of the synthesis, after deprotection of the N-terminal amine with a solution of a 20% piperidine in DMF, the PNA was subjected to derivatization with biotin. Five equivalents of N-biotinyl-aminohexanoic acid were dissolved in a solution of HATU (4.9 eq) in DMF (0.5 M) and DIPEA (7 eq) and reacted for 1 h. The reaction was performed twice to improve the coupling efficiency. Cleavage of the oligomers from the resin and deprotection were carried out as reported elsewhere [35].

### Fluorescent PNAs

Solid-phase synthesis of TO-PNA1-R8 was performed. The peptide (R8) was assembled following previously described procedures [15]. Next the PNA oligomer was assembled, as described above. We conjugated the fluorescent dye TO following previously described procedures [36, 37]. All PNA oligomers were purified and then characterized by liquid chromatography–mass spectrometry using a linear gradient of acetonitrile (0.05% trifluoroacetic acid) in water (0.05% trifluoroacetic acid) from 5% to 50% over 30 min. The sequences of PNA oligomers and mass spectrometry data of all the PNA used for this study are reported in Supplementry Table S6.

### PNA-596 and PNA-anti-uc.8+ transfections

J82 cells (150,000 cells per well) were seeded in 6-well plates and were incubated for approximately 1 h at 37°C. After incubation, cells were transfected with PNA-596 and PNA TO-PNA1-R8 using HiPerFect Transfection Reagent, at a final concentration of 200 nM for PNA-596 and 4 μM for TO-PNA1-R8. The transfected J82 cells were harvested after 48 h. The experiments were conducted in technician triplicate and in biological replicate.

### Statistical analysis

All statistical analyses were performed using the R programming language (version 3.0.1). Each experiment was done at least twice, and at least one of the two sets of experiments was done in triplicate. The differences in T-UCR expression between tumor and non-tumor tissue were compared pairwise using Significant Analysis of Microarray software (Stanford University). Comparisons between independent samples were performed using either the Student t test or the nonparametric Mann-Whitney U test with Bonferroni correction when appropriate, as appropriate. Comparison between dependent samples was performed using the nonparametric Wilcoxon signed-rank test for matched pairs. Unless otherwise specified, data are summarized and graphically represented as means ± SDs. Correlations were computed using the Spearman correlation coefficient. Two-sided P values less than 0.05 were considered statistically significant.

### Data and material availability

J82 cells are commercially available from the American Type Culture Collection (catalog number HTB-1), and RT112 cells are available from the European Collection of Cell Cultures (catalog number 85061106).

## SUPPLEMENTARY FIGURES AND TABLES




