# Supplementary material for: Long non-coding RNA containing ultraconserved genomic region 8 promotes bladder cancer tumorigenesis
Source: Oncotarget. 2016 Mar 1;7(15):20636–54. doi: 10.18632/oncotarget.7833 (PMC4991481; doi:10.18632/oncotarget.7833)
Supplement: Supplementary file 1 [file oncotarget-07-20636-s001.pdf]

SUPPLEMENTARY FIGURES AND TABLES

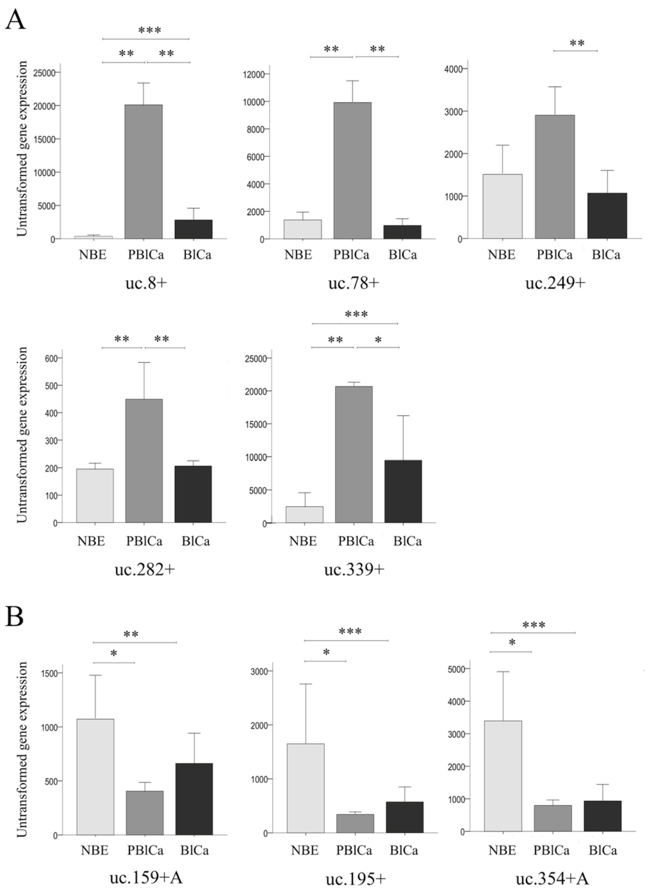

Supplementary Figure S1: Aberrant expression of transcribed ultraconserved RNAs in bladder cancer (BlCa) and pericancerous BlCa (PBlCa) patient samples and normal bladder epithelium (NBE) samples (A and B). The levels, expressed as means ± standard deviation of triplicate values, of untransformed gene expression in NBE, PBlCa, and primary BlCa are shown. Only ultraconserved RNA (uc).8+ and uc.339+ exhibited significant variation in expression between NBE, PBlCa, and BlCa samples. P values were obtained using the Mann-Whitney U test with Bonferroni correction for multiple comparisons. *P<0.05, **P<0.01, and ***P<0.001.

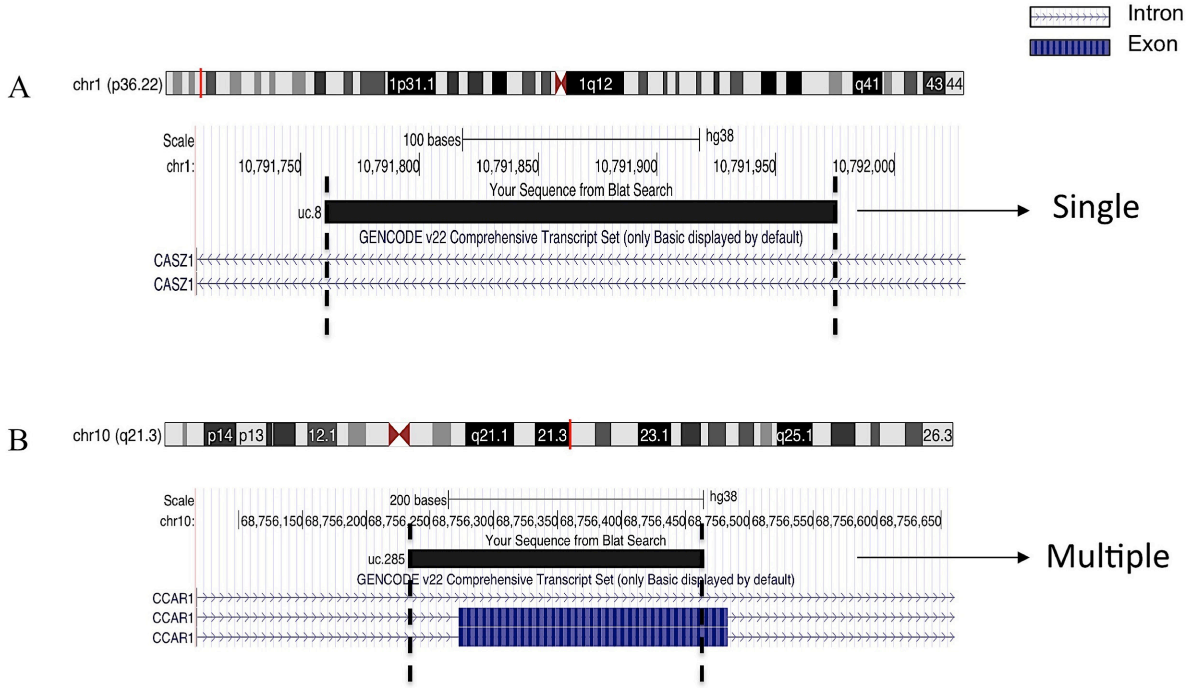

Supplementary Figure S2: Re-classification of transcribed ultraconserved regions (T-UCRs) with respect to the transcript. A. Since a gene may have multiple transcripts, the same T-UCR can have multiple localizations. Ultraconserved RNA (uc).8+ is always intronic in both transcripts of *CASZ1*. **B.** In the three transcripts of CCAR1, uc.285+ is both intronic and exon containing. This classification is critical to discriminate the host gene from T-UCRs.

A

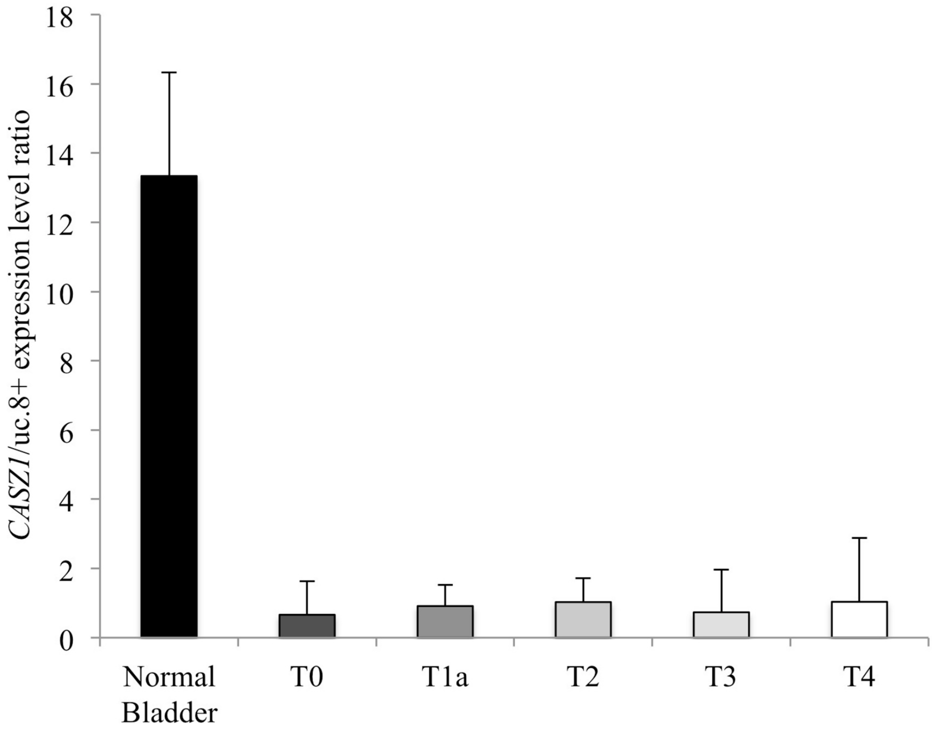

Supplementary Figure S3: CASZ1 ultraconserved RNA (uc). 8+ expression ratios in patients with bladder cancer (BlCa) during cancer progression. *CASZ1*:uc.8+ relative expression ratios at different stages of tumor progression, different shades of gray in the bars are shown. Data are expressed as the means ± standard deviation of triplicate values.

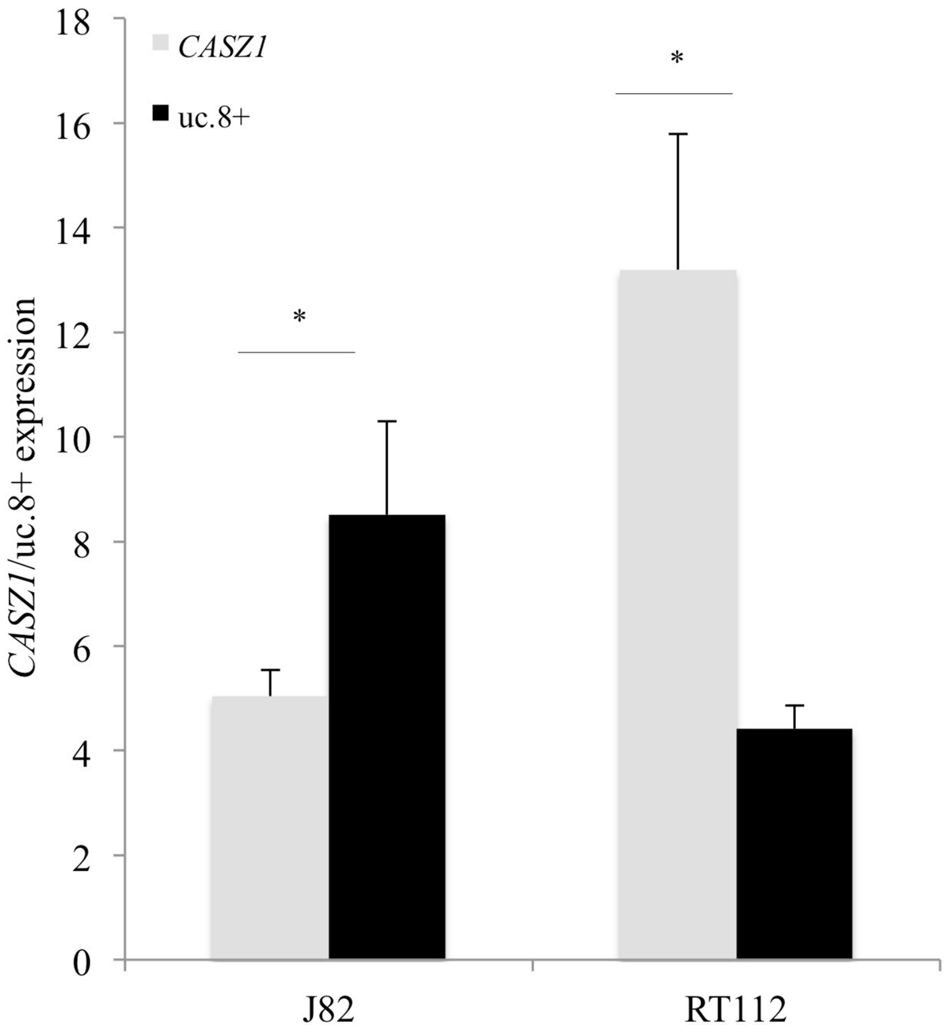

Supplementary Figure S4: Expression of ultraconserved RNA (uc). 8+ in J82 and RT112 bladder cancer cells. We performed qRT-PCR analysis of uc.8+ and *CASZ1* host gene expression in two different cell lines. Data are represented as means ± standard deviation of relative expression of uc.8+ in three experiments performed four times in triplicate. P values were obtained using the Mann-Whitney U test. *P<0.05.

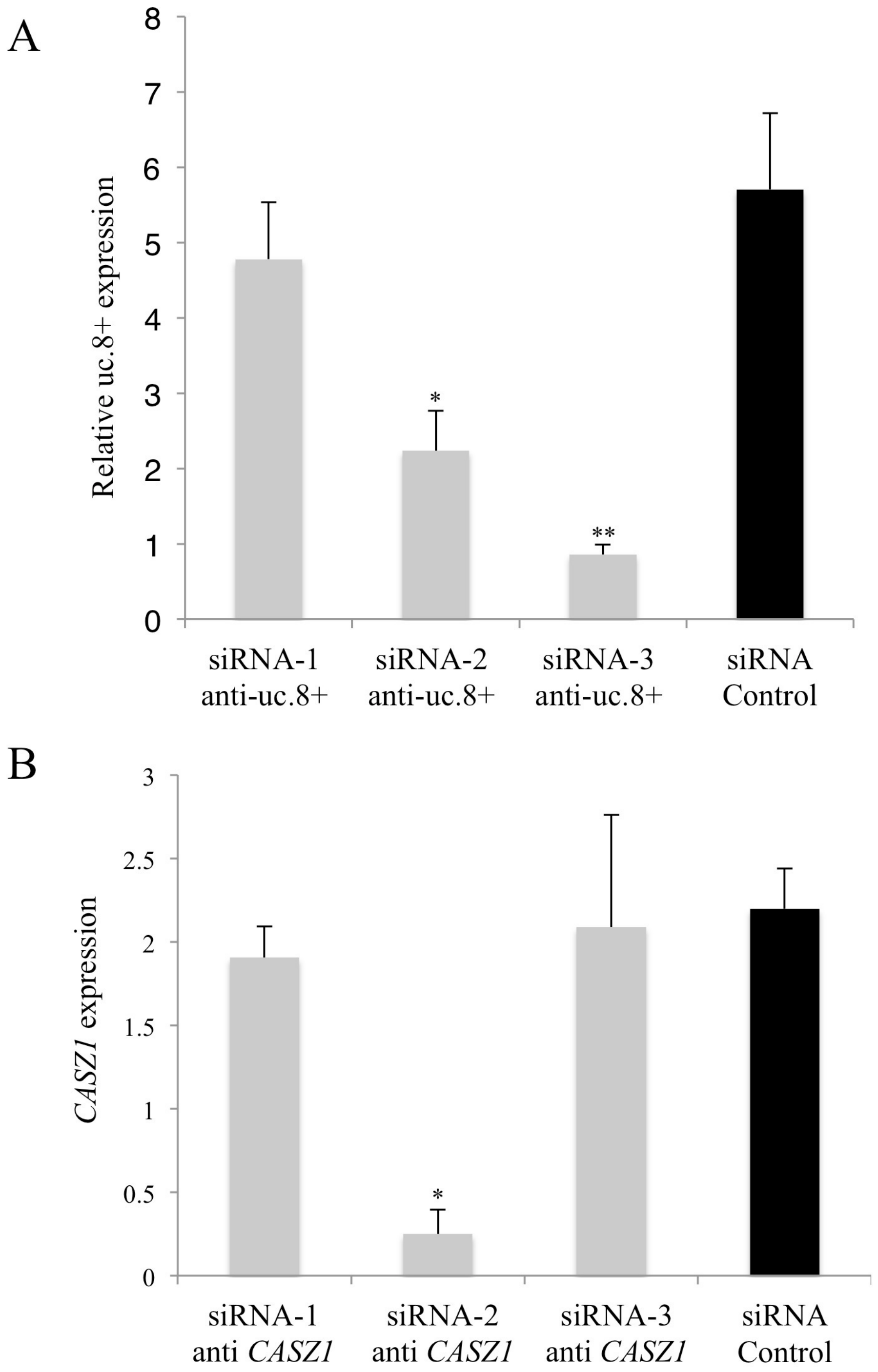

Supplementary Figure S5: Efficiency of silencing ultraconserved RNA (uc). 8+ and CASZ1 in J82 cells. A. Expression of uc.8+ after transfection with three different siRNAs anti-uc.8+. Of the three different siRNAs used, we found that siRNA-3 anti-uc. 8+ was the most efficient in decreasing uc.8+ expression (88%), compared with siRNA control. Means ± standard deviation (SD) are shown. **B.** *CASZ1* expression in J82 cells transfected with three different siRNAs anti-CASZ1. Means ± SD are shown. P values were obtained using the Mann-Whitney U test. *P<0.05 and **P<0.01.

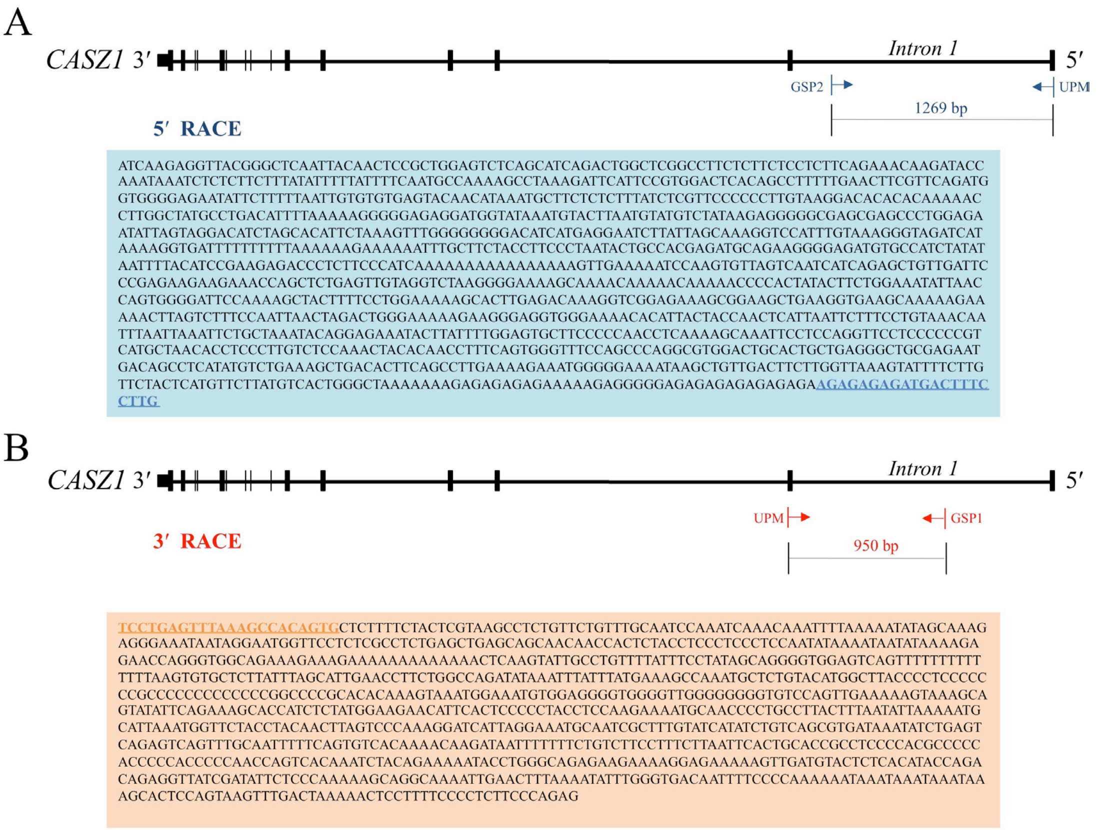

Supplementary Figure S6: Cloned transcript including ultraconserved RNA (uc). 8+. **A.** At the top of figure, the schematic representation of the transcript includes uc.8+ in relation to *CASZ1*. Sequence of PCR products generated by using gene-specific primer 2 (GSP2) and Universal Primer Mix (UPM). The blue sequence shows the 5′ rapid amplification of cDNA ends (RACE) primers and intronic sense primer 2 (ISP2). **B.** Sequence of PCR products generated using GSP1 and UPM primers. The orange sequence shows 3′ RACE primers and ISP1. By performing 5′ and 3′ RACE, we identified 950 nt at the 3′ end and 1269 nt at 5′ of the ultraconserved sequence identified by Bejerano *et al* [1].

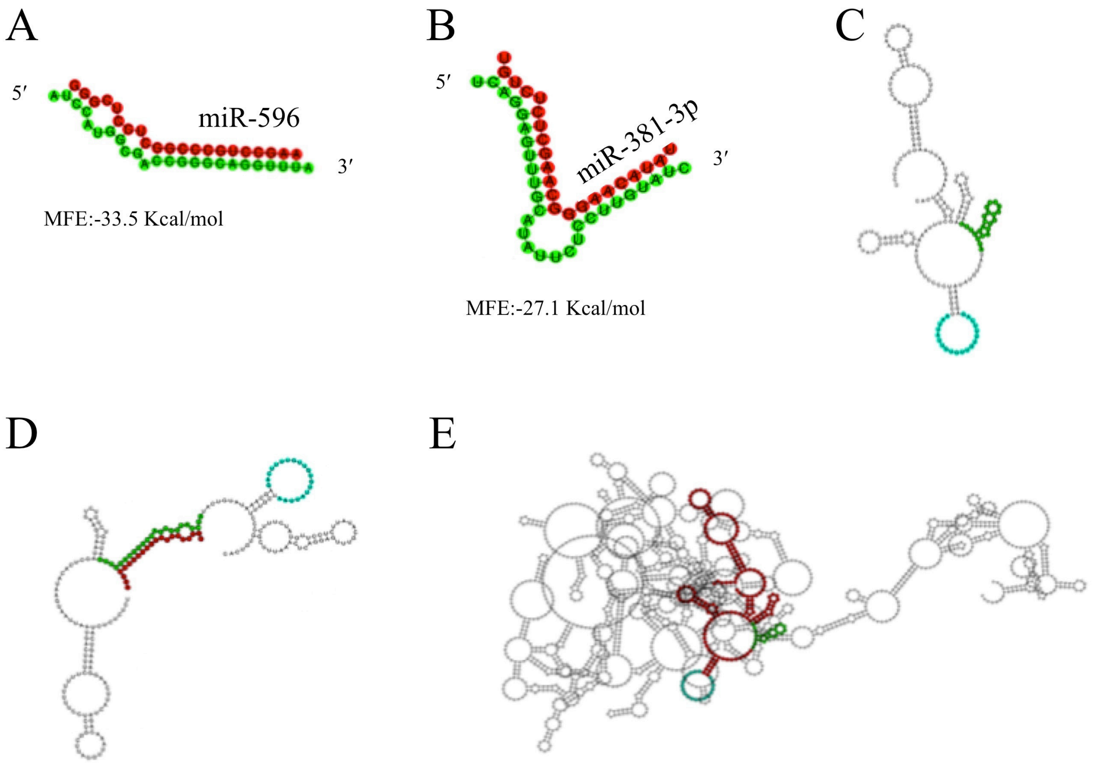

Supplementary Figure S7: Ultraconserved RNA (uc). 8+ secondary structure and microRNA (miR) binding prediction for uc.8+. Graphic representation of the predicted interaction of the two uc.8+ binding sites (green) for **A.** miR-596 and **B.** miR-381-3p (miR-596 and miR-381-3p are shown in red). Minimum free energy (MFE) values for the binding sites are indicated. Predicted RNA secondary structure of **C.** uc.8+ alone and **D.** uc.8+ co-folded with miR-596. The uc.8+ target sequence (green) for miR-596 (red) is shown. Also shown is the sequence of the predicted RNA secondary structure of uc.8+ structurally conserved after miR-596 binding and selected for the designed antisense peptide nucleic acid (PNA)/uc.8+ probe (light blue). **E.** The RNA secondary structure of the complete uc.8+ transcript as predicted using the RNAfold browser. The predicted RNA structure of uc.8+ is shown in red, the predicted binding site for miR-596 is highlighted in green, and the single-strand sequence used for the fishing experiments with a complementary PNA/uc.8+ oligomer (as shown in C and D) is shown in light blue.

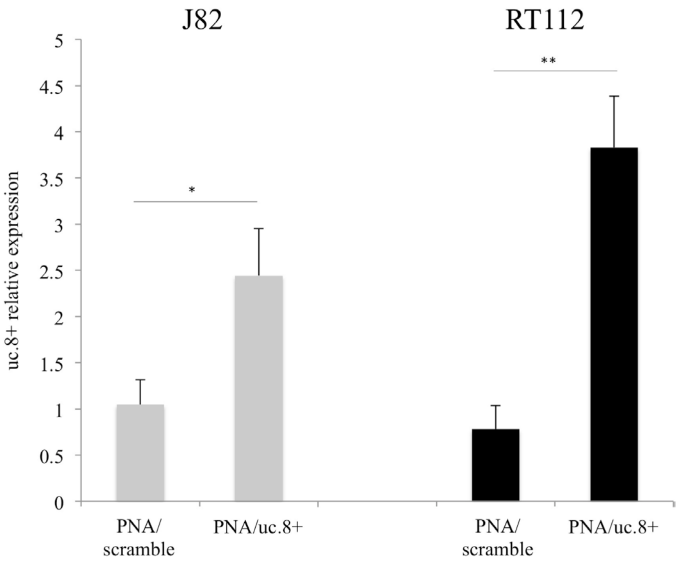

Supplementary Figure S8: Yield of ultraconserved RNA (uc).8+ expression in J82 cells after the fishing/competition experiment. uc.8+ expression increased in J82 cells after fishing with the complementary peptide nucleic acid (PNA)/uc.8+ oligomer CTGAAAACAACACAATAA. A complementary PNA/uc.8+ oligomer effectively retrieved about 2.5 times more uc.8+ than the PNA scramble. Means ± standard deviation are shown. *P<0.05 and **P<0.01.

Supplementary Table S1: Comparison of top-ranked transcribed ultraconserved regions (T-UCRs) in bladder cancer (BlCa) and normal bladder epithelium samples* on the basis of fold change.

See Supplementary File 1

Supplementary Table S2: Comparison of top-ranked transcribed ultraconserved regions (T-UCRs) in bladder cancer (BlCa) and pericancerous BlCa (PBlCa) samples* on the basis of fold change.

See Supplementary File 1

Supplementary Table S3: Genomic features of transcribed ultraconserved regions (T-UCRs).

See Supplementary File 1

Supplementary Table S5: Primers used in the study.

See Supplementary File 1

Supplementary Table S4: Identification of open reading frames starting with an ATG codon in the uc.8+ transcript.

Strand	Frame	DNA start	DNA end	DNA_seq	Prot_seq
Direct	1	262	366	ATGCTTCTCTCTTTATCTCGTTCCCCCCTTG TAAGGACACACACAAAAACCT TGGCTATGCCTGACATTTTAAAAAGGGGGAG AGGATGGTATAAATGTACTTAA	MLLSLSRSPLVRTHTKTL AMPDILKRGRGWYKCT*
		1900	1950	ATGGAAATGTGGAGGGGTGGGGTTGGGG GGGGTGTCCAGTTGAAAAAGTAA	MEMWRGGVGGGVQLKK*
	2	2054	2074	ATGGTTCTACCTACAACTTAG	MVLPTT*
	3	357	410	ATGTACTTAATGTATGTCTATAAGAGGGG GCGAGCGAGCCCTGGAGAATATTAG	MYLMYVYKRGRASPGEY*
		1323	1340	ATGATCTGTTTAACCTAA	MICLT*
Reverse	1	796	825	ATGTTCTTCCATAGAGATGGTGCTTTCTGA	MFFHRDGAF*
		2203	2241	ATGGCACATCTCCCCTTCTGCATC TCGTGGCAGTATTAG	MAHLPFCISWQY*
		2368	2379	ATGTCCTACTAA	MSY*

*STOP codon.
Abbreviations: DNA_seq, DNA sequence; Prot_seq, protein sequence.

Supplementary Table S6. Sequence of PNA oligomers and Mass Spectrometry data.

Name	Sequence	Molecular weight (Da)
PNA1	Biotin-HAx-CTGAAAACAACACAATAA-NH_2	5213.19 (calculated: 5213.18)
PNA 2	Biotin-HAx-CAATAATTAGCAAAGGGGA-NH_2	5591.30 (calculated: 5591.49)
PNA scramble	Biotin-HAx-ACATAACATAACGAACAA-NH_2	5213.19 (calculated: 5213.18)
TO-PNA1 -R8	TO- CTGAAAACAACACAATAA-R8	6510.09 (calculated: 6510.22)
PNA-596	AAGCCTGCCCGGCTCCTCGGG-R8	5967.72 (calculated: 5967.72)
