# Supplementary material for: Long non-coding RNA containing ultraconserved genomic region 8 promotes bladder cancer tumorigenesis
Source: Oncotarget. 2016 Mar 1;7(15):20636–54. doi: 10.18632/oncotarget.7833 (PMC4991481; doi:10.18632/oncotarget.7833)
Supplement: Supplementary file 2 [file oncotarget-07-20636-s002.docx]

**Supplementary Tables**

**Supplementary Table S1. Comparison of top-ranked transcribed ultraconserved regions (T-UCRs) in bladder cancer (BlCa) and normal bladder epithelium samples* on the basis of fold change.**

| **Gene** | **Score**  **(d)^1^** | **Numerator (r)^2^** | **Denominator**  **(s + s0)^3^** | **Fold change in BlCa samples** | **q Value (%)** | **Up (+) /down (-)** |
| --- | --- | --- | --- | --- | --- | --- |
| uc.8+ | 8.782 | 2.735 | 0.311 | 6.657 | 0.000 | + |
| uc.282+A | 6.393 | 1.850 | 0.289 | 3.605 | 0.000 | + |
| uc.339+ | 4.451 | 1.838 | 0.413 | 3.576 | 0.000 | + |
| uc.283+A | 6.033 | 1.762 | 0.292 | 3.391 | 0.000 | + |
| uc.346+ | 7.618 | 1.318 | 0.173 | 2.494 | 0.000 | + |
| uc.369+ | 7.456 | 1.313 | 0.176 | 2.484 | 0.000 | + |
| uc.145+A | 4.234 | 1.218 | 0.288 | 2.326 | 0.000 | + |
| uc.31+ | 3.688 | 1.212 | 0.329 | 2.317 | 0.000 | + |
| uc.230+ | 3.204 | 1.172 | 0.366 | 2.254 | 0.000 | + |
| uc.160+ | 5.674 | 1.108 | 0.195 | 2.156 | 0.000 | + |
| uc.325+ | 5.076 | 1.106 | 0.218 | 2.153 | 0.000 | + |
| uc.466+A | 5.908 | 1.067 | 0.181 | 2.094 | 0.000 | + |
| uc.278+ | 4.365 | 1.000 | 0.229 | 1.999 | 0.000 | + |
| uc.359+A | 4.376 | 0.952 | 0.218 | 1.934 | 0.000 | + |
| uc.269+A | 3.971 | 0.862 | 0.217 | 1.817 | 0.000 | + |
| uc.266+A | 3.870 | 0.824 | 0.213 | 1.770 | 0.000 | + |
| uc.327+A | 5.104 | 0.822 | 0.161 | 1.768 | 0.000 | + |
| uc.18+ | 2.903 | 0.788 | 0.271 | 1.726 | 0.000 | + |
| uc.398+ | 3.020 | 0.749 | 0.248 | 1.680 | 0.000 | + |
| uc.136+ | 5.143 | 0.743 | 0.144 | 1.673 | 0.000 | + |
| uc.319+A | 4.007 | 0.739 | 0.184 | 1.669 | 0.000 | + |
| uc.84+A | 2.582 | 0.722 | 0.280 | 1.650 | 0.000 | + |
| uc.244+ | 5.357 | 0.719 | 0.134 | 1.646 | 0.000 | + |
| uc.346+A | 3.019 | 0.700 | 0.232 | 1.624 | 0.000 | + |
| uc.326+ | 2.963 | 0.688 | 0.232 | 1.611 | 0.000 | + |
| uc.352+ | 3.073 | 0.665 | 0.216 | 1.585 | 0.000 | + |
| uc.139+ | 2.234 | 0.585 | 0.262 | 1.500 | 1.020 | + |
| uc.129+ | 3.205 | 0.567 | 0.177 | 1.481 | 0.000 | + |
| uc.390+ | 2.403 | 0.553 | 0.230 | 1.467 | 0.380 | + |
| uc.9+A | 3.766 | 0.517 | 0.137 | 1.431 | 0.000 | + |
| uc.317+ | 3.508 | 0.489 | 0.139 | 1.403 | 0.000 | + |
| uc.349+ | 4.072 | 0.486 | 0.119 | 1.400 | 0.000 | + |
| uc.257+ | 2.236 | 0.475 | 0.212 | 1.390 | 1.020 | + |
| uc.366+ | 2.672 | 0.469 | 0.176 | 1.384 | 0.000 | + |
| uc.422+ | 2.234 | 0.465 | 0.208 | 1.380 | 1.020 | + |
| uc.302+A | 3.746 | 0.438 | 0.117 | 1.355 | 0.000 | + |
| uc.335+ | 2.417 | 0.430 | 0.178 | 1.347 | 0.380 | + |
| uc.192+A | 4.104 | 0.424 | 0.103 | 1.342 | 0.000 | + |
| uc.1+ | 2.918 | 0.423 | 0.145 | 1.341 | 0.000 | + |
| uc.399+A | 3.846 | 0.423 | 0.110 | 1.340 | 0.000 | + |
| uc.24+A | 2.384 | 0.416 | 0.175 | 1.335 | 0.380 | + |
| uc.241+A | 2.591 | 0.413 | 0.159 | 1.331 | 0.000 | + |
| uc.252+A | 3.068 | 0.401 | 0.131 | 1.320 | 0.000 | + |
| uc.172+A | 3.512 | 0.397 | 0.113 | 1.317 | 0.000 | + |
| uc.304+A | 2.959 | 0.378 | 0.128 | 1.300 | 0.000 | + |
| uc.414+ | 2.721 | 0.349 | 0.128 | 1.274 | 0.000 | + |
| uc.458+A | 2.533 | 0.350 | 0.138 | 1.274 | 0.202 | + |
| uc.73+ | 3.576 | 0.348 | 0.097 | 1.273 | 0.000 | + |
| uc.44+ | 2.601 | 0.347 | 0.134 | 1.272 | 0.000 | + |
| uc.452+ | 2.804 | 0.341 | 0.122 | 1.266 | 0.000 | + |
| uc.396+ | 2.656 | 0.309 | 0.116 | 1.239 | 0.000 | + |
| uc.482+A | 2.482 | 0.310 | 0.125 | 1.239 | 0.202 | + |
| uc.343+A | 2.682 | 0.300 | 0.112 | 1.232 | 0.000 | + |
| uc.121+A | 3.273 | 0.299 | 0.091 | 1.230 | 0.000 | + |
| uc.54+ | 3.627 | 0.285 | 0.079 | 1.219 | 0.000 | + |
| uc.334+ | 2.586 | 0.279 | 0.108 | 1.213 | 0.000 | + |
| uc.238+A | 2.299 | 0.274 | 0.119 | 1.209 | 0.380 | + |
| uc.201+ | 3.882 | 0.263 | 0.068 | 1.200 | 0.000 | + |
| uc.343+ | 2.551 | 0.261 | 0.102 | 1.199 | 0.202 | + |
| uc.68+ | 2.481 | 0.262 | 0.106 | 1.199 | 0.202 | + |
| uc.399+ | 2.577 | 0.253 | 0.098 | 1.192 | 0.000 | + |
| uc.419+ | 2.655 | 0.248 | 0.093 | 1.187 | 0.000 | + |
| uc.14+ | 3.099 | 0.235 | 0.076 | 1.177 | 0.000 | + |
| uc.283+ | 2.750 | 0.231 | 0.084 | 1.174 | 0.000 | + |
| uc.421+ | 2.219 | 0.220 | 0.099 | 1.165 | 1.020 | + |
| uc.416+ | 2.423 | 0.218 | 0.090 | 1.163 | 0.202 | + |
| uc.479+ | 2.286 | 0.212 | 0.093 | 1.158 | 1.020 | + |
| uc.56+ | 2.278 | 0.207 | 0.091 | 1.154 | 1.020 | + |
| uc.221+ | 2.364 | 0.200 | 0.085 | 1.149 | 0.380 | + |
| uc.12+A | 2.748 | 0.197 | 0.072 | 1.146 | 0.000 | + |
| uc.291+ | 2.251 | 0.192 | 0.085 | 1.142 | 1.020 | + |
| uc.261+ | 2.396 | 0.177 | 0.074 | 1.130 | 0.380 | + |
| uc.179+ | 2.246 | 0.165 | 0.073 | 1.121 | 1.020 | + |
| uc.247+ | 2.223 | 0.155 | 0.070 | 1.114 | 1.020 | + |
| uc.344+A | 2.220 | 0.152 | 0.068 | 1.111 | 1.020 | + |
| uc.85+A | -1.731 | -0.119 | 0.069 | 0.921 | 1.020 | - |
| uc.274+ | -1.700 | -0.139 | 0.082 | 0.908 | 1.020 | - |
| uc.451+ | -1.834 | -0.143 | 0.078 | 0.906 | 0.380 | - |
| uc.239+A | -1.782 | -0.142 | 0.080 | 0.906 | 0.380 | - |
| uc.12+ | -2.222 | -0.152 | 0.068 | 0.900 | 0.000 | - |
| uc.403+ | -1.736 | -0.156 | 0.090 | 0.898 | 1.020 | - |
| uc.311+ | -1.975 | -0.167 | 0.085 | 0.891 | 0.202 | - |
| uc.59+ | -2.506 | -0.168 | 0.067 | 0.890 | 0.000 | - |
| uc.24+ | -2.520 | -0.179 | 0.071 | 0.883 | 0.000 | - |
| uc.191+A | -2.133 | -0.189 | 0.089 | 0.877 | 0.000 | - |
| uc.30+ | -2.059 | -0.200 | 0.097 | 0.870 | 0.202 | - |
| uc.102+A | -2.379 | -0.204 | 0.086 | 0.868 | 0.000 | - |
| uc.410+ | -2.311 | -0.205 | 0.089 | 0.868 | 0.000 | - |
| uc.276+ | -1.705 | -0.205 | 0.120 | 0.868 | 1.020 | - |
| uc.36+A | -1.815 | -0.206 | 0.113 | 0.867 | 0.380 | - |
| uc.67+ | -1.807 | -0.208 | 0.115 | 0.865 | 0.380 | - |
| uc.358+ | -2.440 | -0.220 | 0.090 | 0.859 | 0.000 | - |
| uc.329+ | -1.808 | -0.227 | 0.125 | 0.855 | 0.380 | - |
| uc.112+A | -2.181 | -0.228 | 0.104 | 0.854 | 0.000 | - |
| uc.227+A | -2.128 | -0.228 | 0.107 | 0.854 | 0.000 | - |
| uc.92+A | -2.181 | -0.230 | 0.105 | 0.853 | 0.000 | - |
| uc.295+A | -1.996 | -0.238 | 0.119 | 0.848 | 0.202 | - |
| uc.289+A | -1.713 | -0.240 | 0.140 | 0.847 | 1.020 | - |
| uc.156+A | -2.308 | -0.251 | 0.109 | 0.840 | 0.000 | - |
| uc.253+ | -2.565 | -0.260 | 0.101 | 0.835 | 0.000 | - |
| uc.115+A | -2.181 | -0.260 | 0.119 | 0.835 | 0.000 | - |
| uc.67+A | -2.153 | -0.262 | 0.122 | 0.834 | 0.000 | - |
| uc.247+A | -2.775 | -0.264 | 0.095 | 0.833 | 0.000 | - |
| uc.6+ | -1.793 | -0.264 | 0.147 | 0.833 | 0.380 | - |
| uc.126+ | -2.276 | -0.274 | 0.120 | 0.827 | 0.000 | - |
| uc.472+A | -2.610 | -0.284 | 0.109 | 0.822 | 0.000 | - |
| uc.471+A | -3.113 | -0.289 | 0.093 | 0.819 | 0.000 | - |
| uc.166+ | -2.453 | -0.291 | 0.119 | 0.817 | 0.000 | - |
| uc.436+ | -2.345 | -0.298 | 0.127 | 0.813 | 0.000 | - |
| uc.208+A | -1.779 | -0.301 | 0.169 | 0.812 | 0.380 | - |
| uc.289+ | -1.940 | -0.304 | 0.157 | 0.810 | 0.380 | - |
| uc.57+ | -1.729 | -0.305 | 0.177 | 0.809 | 1.020 | - |
| uc.321+A | -2.157 | -0.308 | 0.143 | 0.808 | 0.000 | - |
| uc.125+A | -2.034 | -0.307 | 0.151 | 0.808 | 0.202 | - |
| uc.324+A | -2.375 | -0.315 | 0.133 | 0.804 | 0.000 | - |
| uc.180+A | -2.852 | -0.319 | 0.112 | 0.801 | 0.000 | - |
| uc.458+ | -2.390 | -0.325 | 0.136 | 0.798 | 0.000 | - |
| uc.372+A | -1.969 | -0.326 | 0.166 | 0.798 | 0.202 | - |
| uc.302+ | -2.141 | -0.335 | 0.157 | 0.793 | 0.000 | - |
| uc.122+ | -1.688 | -0.335 | 0.199 | 0.793 | 1.020 | - |
| uc.5+A | -3.590 | -0.351 | 0.098 | 0.784 | 0.000 | - |
| uc.417+ | -2.970 | -0.352 | 0.118 | 0.784 | 0.000 | - |
| uc.144+ | -2.274 | -0.358 | 0.157 | 0.780 | 0.000 | - |
| uc.126+A | -3.229 | -0.364 | 0.113 | 0.777 | 0.000 | - |
| uc.185+A | -2.021 | -0.363 | 0.180 | 0.777 | 0.202 | - |
| uc.181+ | -2.163 | -0.374 | 0.173 | 0.772 | 0.000 | - |
| uc.111+ | -2.272 | -0.381 | 0.168 | 0.768 | 0.000 | - |
| uc.381+ | -3.529 | -0.386 | 0.109 | 0.765 | 0.000 | - |
| uc.371+A | -2.254 | -0.389 | 0.173 | 0.764 | 0.000 | - |
| uc.385+A | -1.708 | -0.395 | 0.232 | 0.760 | 1.020 | - |
| uc.180+ | -2.321 | -0.399 | 0.172 | 0.758 | 0.000 | - |
| uc.122+A | -2.115 | -0.399 | 0.189 | 0.758 | 0.000 | - |
| uc.454+ | -3.032 | -0.416 | 0.137 | 0.750 | 0.000 | - |
| uc.141+ | -2.177 | -0.415 | 0.190 | 0.750 | 0.000 | - |
| uc.465+ | -1.983 | -0.421 | 0.213 | 0.747 | 0.202 | - |
| uc.377+A | -2.052 | -0.426 | 0.208 | 0.744 | 0.202 | - |
| uc.208+ | -2.235 | -0.432 | 0.193 | 0.741 | 0.000 | - |
| uc.223+A | -2.333 | -0.435 | 0.186 | 0.740 | 0.000 | - |
| uc.274+A | -3.812 | -0.437 | 0.115 | 0.739 | 0.000 | - |
| uc.77+ | -2.057 | -0.436 | 0.212 | 0.739 | 0.202 | - |
| uc.10+A | -1.798 | -0.437 | 0.243 | 0.739 | 0.380 | - |
| uc.196+ | -2.219 | -0.441 | 0.199 | 0.737 | 0.000 | - |
| uc.476+ | -2.291 | -0.445 | 0.194 | 0.735 | 0.000 | - |
| uc.425+A | -3.195 | -0.446 | 0.139 | 0.734 | 0.000 | - |
| uc.446+ | -2.072 | -0.449 | 0.217 | 0.733 | 0.202 | - |
| uc.137+A | -4.648 | -0.453 | 0.097 | 0.731 | 0.000 | - |
| uc.359+ | -2.824 | -0.471 | 0.167 | 0.721 | 0.000 | - |
| uc.405+A | -3.520 | -0.474 | 0.135 | 0.720 | 0.000 | - |
| uc.345+A | -2.210 | -0.473 | 0.214 | 0.720 | 0.000 | - |
| uc.402+ | -4.002 | -0.479 | 0.120 | 0.717 | 0.000 | - |
| uc.218+ | -3.872 | -0.489 | 0.126 | 0.713 | 0.000 | - |
| uc.340+ | -2.117 | -0.488 | 0.231 | 0.713 | 0.000 | - |
| uc.269+ | -2.508 | -0.490 | 0.195 | 0.712 | 0.000 | - |
| uc.88+A | -1.800 | -0.506 | 0.281 | 0.704 | 0.380 | - |
| uc.183+ | -1.771 | -0.509 | 0.287 | 0.703 | 1.020 | - |
| uc.476+A | -2.286 | -0.510 | 0.223 | 0.702 | 0.000 | - |
| uc.333+A | -3.357 | -0.513 | 0.153 | 0.701 | 0.000 | - |
| uc.77+A | -1.724 | -0.513 | 0.297 | 0.701 | 1.020 | - |
| uc.249+ | -1.886 | -0.521 | 0.276 | 0.697 | 0.380 | - |
| uc.305+A | -1.986 | -0.524 | 0.264 | 0.696 | 0.202 | - |
| uc.215+A | -3.994 | -0.526 | 0.132 | 0.694 | 0.000 | - |
| uc.432+ | -3.184 | -0.526 | 0.165 | 0.694 | 0.000 | - |
| uc.149+A | -2.121 | -0.527 | 0.248 | 0.694 | 0.000 | - |
| uc.70+ | -3.217 | -0.529 | 0.164 | 0.693 | 0.000 | - |
| uc.393+ | -3.852 | -0.547 | 0.142 | 0.684 | 0.000 | - |
| uc.128+ | -2.214 | -0.555 | 0.251 | 0.681 | 0.000 | - |
| uc.382+A | -2.825 | -0.559 | 0.198 | 0.679 | 0.000 | - |
| uc.185+ | -2.704 | -0.559 | 0.207 | 0.679 | 0.000 | - |
| uc.398+A | -2.657 | -0.557 | 0.210 | 0.679 | 0.000 | - |
| uc.281+ | -1.867 | -0.558 | 0.299 | 0.679 | 0.380 | - |
| uc.78+ | -2.218 | -0.563 | 0.254 | 0.677 | 0.000 | - |
| uc.63+ | -1.834 | -0.565 | 0.308 | 0.676 | 0.380 | - |
| uc.265+A | -2.440 | -0.566 | 0.232 | 0.675 | 0.000 | - |
| uc.443+A | -3.461 | -0.576 | 0.166 | 0.671 | 0.000 | - |
| uc.146+ | -2.519 | -0.576 | 0.228 | 0.671 | 0.000 | - |
| uc.296+ | -4.663 | -0.578 | 0.124 | 0.670 | 0.000 | - |
| uc.420+ | -2.285 | -0.577 | 0.253 | 0.670 | 0.000 | - |
| uc.102+ | -3.354 | -0.592 | 0.176 | 0.664 | 0.000 | - |
| uc.61+A | -3.041 | -0.599 | 0.197 | 0.660 | 0.000 | - |
| uc.10+ | -2.185 | -0.609 | 0.279 | 0.656 | 0.000 | - |
| uc.306+ | -3.317 | -0.631 | 0.190 | 0.646 | 0.000 | - |
| uc.97+A | -4.419 | -0.635 | 0.144 | 0.644 | 0.000 | - |
| uc.323+ | -3.242 | -0.639 | 0.197 | 0.642 | 0.000 | - |
| uc.140+A | -3.618 | -0.651 | 0.180 | 0.637 | 0.000 | - |
| uc.168+A | -4.396 | -0.663 | 0.151 | 0.632 | 0.000 | - |
| uc.157+A | -3.189 | -0.664 | 0.208 | 0.631 | 0.000 | - |
| uc.356+ | -3.944 | -0.666 | 0.169 | 0.630 | 0.000 | - |
| uc.292+ | -2.167 | -0.666 | 0.308 | 0.630 | 0.000 | - |
| uc.13+A | -3.423 | -0.671 | 0.196 | 0.628 | 0.000 | - |
| uc.453+A | -2.625 | -0.699 | 0.266 | 0.616 | 0.000 | - |
| uc.134+ | -4.428 | -0.707 | 0.160 | 0.613 | 0.000 | - |
| uc.159+A | -2.986 | -0.706 | 0.236 | 0.613 | 0.000 | - |
| uc.158+A | -3.506 | -0.713 | 0.203 | 0.610 | 0.000 | - |
| uc.450+A | -2.496 | -0.712 | 0.285 | 0.610 | 0.000 | - |
| uc.449+A | -4.072 | -0.716 | 0.176 | 0.609 | 0.000 | - |
| uc.402+A | -1.918 | -0.715 | 0.373 | 0.609 | 0.380 | - |
| uc.143+A | -3.451 | -0.723 | 0.209 | 0.606 | 0.000 | - |
| uc.432+A | -2.082 | -0.724 | 0.348 | 0.605 | 0.202 | - |
| uc.149+ | -3.577 | -0.730 | 0.204 | 0.603 | 0.000 | - |
| uc.19+ | -3.858 | -0.734 | 0.190 | 0.601 | 0.000 | - |
| uc.403+A | -5.177 | -0.740 | 0.143 | 0.599 | 0.000 | - |
| uc.408+A | -4.140 | -0.740 | 0.179 | 0.599 | 0.000 | - |
| uc.162+A | -2.705 | -0.740 | 0.274 | 0.599 | 0.000 | - |
| uc.397+A | -4.449 | -0.746 | 0.168 | 0.596 | 0.000 | - |
| uc.188+ | -4.613 | -0.749 | 0.162 | 0.595 | 0.000 | - |
| uc.322+A | -3.175 | -0.760 | 0.239 | 0.591 | 0.000 | - |
| uc.334+A | -4.466 | -0.766 | 0.172 | 0.588 | 0.000 | - |
| uc.360+ | -4.028 | -0.767 | 0.190 | 0.588 | 0.000 | - |
| uc.170+A | -2.276 | -0.770 | 0.338 | 0.587 | 0.000 | - |
| uc.162+ | -3.620 | -0.777 | 0.215 | 0.584 | 0.000 | - |
| uc.310+ | -2.445 | -0.792 | 0.324 | 0.578 | 0.000 | - |
| uc.150+ | -5.436 | -0.819 | 0.151 | 0.567 | 0.000 | - |
| uc.220+ | -4.523 | -0.819 | 0.181 | 0.567 | 0.000 | - |
| uc.299+A | -2.085 | -0.829 | 0.398 | 0.563 | 0.202 | - |
| uc.17+A | -3.692 | -0.835 | 0.226 | 0.561 | 0.000 | - |
| uc.377+ | -3.751 | -0.836 | 0.223 | 0.560 | 0.000 | - |
| uc.322+ | -4.859 | -0.851 | 0.175 | 0.555 | 0.000 | - |
| uc.182+ | -2.389 | -0.850 | 0.356 | 0.555 | 0.000 | - |
| uc.285+ | -3.282 | -0.853 | 0.260 | 0.553 | 0.000 | - |
| uc.173+A | -4.737 | -0.866 | 0.183 | 0.549 | 0.000 | - |
| uc.305+ | -2.469 | -0.869 | 0.352 | 0.547 | 0.000 | - |
| uc.170+ | -2.383 | -0.876 | 0.368 | 0.545 | 0.000 | - |
| uc.440+A | -4.528 | -0.883 | 0.195 | 0.542 | 0.000 | - |
| uc.246+ | -3.034 | -0.889 | 0.293 | 0.540 | 0.000 | - |
| uc.276+A | -5.047 | -0.894 | 0.177 | 0.538 | 0.000 | - |
| uc.435+ | -3.943 | -0.908 | 0.230 | 0.533 | 0.000 | - |
| uc.133+A | -3.704 | -0.928 | 0.251 | 0.526 | 0.000 | - |
| uc.389+ | -3.970 | -0.933 | 0.235 | 0.524 | 0.000 | - |
| uc.350+ | -3.615 | -0.934 | 0.258 | 0.523 | 0.000 | - |
| uc.47+ | -3.081 | -0.939 | 0.305 | 0.521 | 0.000 | - |
| uc.163+ | -4.778 | -0.966 | 0.202 | 0.512 | 0.000 | - |
| uc.392+A | -4.974 | -0.977 | 0.196 | 0.508 | 0.000 | - |
| uc.225+A | -4.800 | -0.978 | 0.204 | 0.508 | 0.000 | - |
| uc.177+A | -4.057 | -0.978 | 0.241 | 0.508 | 0.000 | - |
| uc.190+A | -3.373 | -0.981 | 0.291 | 0.507 | 0.000 | - |
| uc.88+ | -3.191 | -1.002 | 0.314 | 0.499 | 0.000 | - |
| uc.200+ | -3.506 | -1.006 | 0.287 | 0.498 | 0.000 | - |
| uc.324+ | -3.080 | -1.010 | 0.328 | 0.497 | 0.000 | - |
| uc.151+A | -3.377 | -1.039 | 0.308 | 0.487 | 0.000 | - |
| uc.21+A | -4.969 | -1.043 | 0.210 | 0.485 | 0.000 | - |
| uc.456+A | -3.719 | -1.046 | 0.281 | 0.484 | 0.000 | - |
| uc.374+A | -5.460 | -1.056 | 0.193 | 0.481 | 0.000 | - |
| uc.363+A | -4.458 | -1.059 | 0.238 | 0.480 | 0.000 | - |
| uc.34+A | -4.399 | -1.059 | 0.241 | 0.480 | 0.000 | - |
| uc.44+A | -3.721 | -1.096 | 0.295 | 0.468 | 0.000 | - |
| uc.427+ | -3.109 | -1.101 | 0.354 | 0.466 | 0.000 | - |
| uc.138+ | -5.052 | -1.103 | 0.218 | 0.465 | 0.000 | - |
| uc.28+ | -4.747 | -1.118 | 0.236 | 0.461 | 0.000 | - |
| uc.275+A | -4.580 | -1.127 | 0.246 | 0.458 | 0.000 | - |
| uc.43+ | -3.592 | -1.128 | 0.314 | 0.457 | 0.000 | - |
| uc.298+ | -5.581 | -1.138 | 0.204 | 0.454 | 0.000 | - |
| uc.4+ | -5.374 | -1.144 | 0.213 | 0.452 | 0.000 | - |
| uc.372+ | -4.544 | -1.160 | 0.255 | 0.448 | 0.000 | - |
| uc.404+ | -5.633 | -1.161 | 0.206 | 0.447 | 0.000 | - |
| uc.288+A | -5.623 | -1.162 | 0.207 | 0.447 | 0.000 | - |
| uc.412+A | -4.500 | -1.186 | 0.264 | 0.439 | 0.000 | - |
| uc.457+ | -4.741 | -1.206 | 0.254 | 0.434 | 0.000 | - |
| uc.96+ | -4.960 | -1.231 | 0.248 | 0.426 | 0.000 | - |
| uc.268+ | -4.663 | -1.271 | 0.273 | 0.414 | 0.000 | - |
| uc.189+ | -4.552 | -1.306 | 0.287 | 0.404 | 0.000 | - |
| uc.234+ | -4.115 | -1.319 | 0.320 | 0.401 | 0.000 | - |
| uc.89+ | -5.368 | -1.330 | 0.248 | 0.398 | 0.000 | - |
| uc.342+ | -5.263 | -1.352 | 0.257 | 0.392 | 0.000 | - |
| uc.195+ | -4.481 | -1.363 | 0.304 | 0.389 | 0.000 | - |
| uc.206+A | -5.476 | -1.384 | 0.253 | 0.383 | 0.000 | - |
| uc.16+ | -4.986 | -1.399 | 0.281 | 0.379 | 0.000 | - |
| uc.468+ | -5.311 | -1.410 | 0.265 | 0.376 | 0.000 | - |
| uc.48+A | -4.405 | -1.417 | 0.322 | 0.374 | 0.000 | - |
| uc.3+ | -6.007 | -1.450 | 0.241 | 0.366 | 0.000 | - |
| uc.450+ | -6.034 | -1.488 | 0.247 | 0.357 | 0.000 | - |
| uc.106+A | -5.188 | -1.490 | 0.287 | 0.356 | 0.000 | - |
| uc.462+A | -4.667 | -1.501 | 0.322 | 0.353 | 0.000 | - |
| uc.448+A | -5.227 | -1.532 | 0.293 | 0.346 | 0.000 | - |
| uc.238+ | -5.163 | -1.595 | 0.309 | 0.331 | 0.000 | - |
| uc.142+A | -5.347 | -1.605 | 0.300 | 0.329 | 0.000 | - |
| uc.388+A | -5.746 | -1.610 | 0.280 | 0.328 | 0.000 | - |
| uc.469+A | -5.802 | -1.612 | 0.278 | 0.327 | 0.000 | - |
| uc.33+ | -5.282 | -1.621 | 0.307 | 0.325 | 0.000 | - |
| uc.362+A | -6.898 | -1.640 | 0.238 | 0.321 | 0.000 | - |
| uc.445+A | -6.035 | -1.646 | 0.273 | 0.319 | 0.000 | - |
| uc.378+A | -4.869 | -1.652 | 0.339 | 0.318 | 0.000 | - |
| uc.477+A | -5.561 | -1.748 | 0.314 | 0.298 | 0.000 | - |
| uc.325+A | -8.001 | -1.796 | 0.224 | 0.288 | 0.000 | - |
| uc.213+A | -5.288 | -1.802 | 0.341 | 0.287 | 0.000 | - |
| uc.473+A | -5.947 | -1.884 | 0.317 | 0.271 | 0.000 | - |
| uc.248+A | -5.932 | -1.884 | 0.318 | 0.271 | 0.000 | - |
| uc.354+A | -6.646 | -1.909 | 0.287 | 0.266 | 0.000 | - |
| uc.153+A | -6.107 | -1.931 | 0.316 | 0.262 | 0.000 | - |
| uc.20+ | -5.863 | -1.935 | 0.330 | 0.262 | 0.000 | - |
| uc.287+A | -5.675 | -1.978 | 0.349 | 0.254 | 0.000 | - |
| uc.229+A | -6.381 | -1.991 | 0.312 | 0.252 | 0.000 | - |
| uc.263+A | -6.363 | -2.119 | 0.333 | 0.230 | 0.000 | - |
| uc.217+A | -6.443 | -2.220 | 0.345 | 0.215 | 0.000 | - |

^1^Score (d) Numerator/Denominator: It is equivalent to a T test statistics.

^2^Numerator (r): Difference of the sample means in the two classes (BlCa and PBlCa). It is equivalent to the numerator of the independent samples T-test statistics.

^3^Denominator (s + s0): Estimate of the standard error of the difference in means. It is obtained summing the usual denominator of the independent samples T-test statistics (s) plus an an exchangeability constant shrinking the scores of genes with expression near 0.

*Clinical characteristics listed in Table 1, data set 1.

Abbreviations: uc, ultraconserved region.

**Supplementary Table S2. Comparison of top-ranked transcribed ultraconserved regions (T-UCRs) in bladder cancer (BlCa) and pericancerous BlCa (PBlCa) samples* on the basis of fold change.**

| **Gene** | **Score**  **(d)^1^** | **Numerator (r)^2^** | **Denominator (s + s0)^3^** | **Fold change in BlCa samples** | **q Value (%)** | **Up (+) / down (-)** |
| --- | --- | --- | --- | --- | --- | --- |
| uc.195+ | 3.736 | 0.957 | 0.256 | 1.941 | 0.000 | + |
| uc.159+A | 2.311 | 0.678 | 0.294 | 1.600 | 2.562 | + |
| uc.264+ | 2.334 | 0.538 | 0.231 | 1.452 | 2.562 | + |
| uc.355+ | 2.402 | 0.515 | 0.214 | 1.429 | 2.562 | + |
| uc.335+A | 2.616 | 0.424 | 0.162 | 1.342 | 2.562 | + |
| uc.187+A | 2.328 | 0.424 | 0.182 | 1.342 | 2.562 | + |
| uc.458+ | -1.534 | -0.298 | 0.194 | 0.813 | 2.562 | - |
| uc.94+A | -1.660 | -0.344 | 0.207 | 0.788 | 1.038 | - |
| uc.344+ | -1.588 | -0.358 | 0.225 | 0.780 | 1.896 | - |
| uc.12+A | -1.637 | -0.360 | 0.220 | 0.779 | 1.038 | - |
| uc.90+ | -2.200 | -0.363 | 0.165 | 0.778 | 0.000 | - |
| uc.297+ | -1.940 | -0.371 | 0.191 | 0.773 | 0.000 | - |
| uc.202+ | -2.071 | -0.374 | 0.181 | 0.771 | 0.000 | - |
| uc.324+ | -1.508 | -0.378 | 0.250 | 0.770 | 2.562 | - |
| uc.125+ | -1.682 | -0.378 | 0.225 | 0.769 | 1.038 | - |
| uc.42+ | -1.798 | -0.381 | 0.212 | 0.768 | 0.634 | - |
| uc.358+A | -2.238 | -0.385 | 0.172 | 0.766 | 0.000 | - |
| uc.102+A | -1.727 | -0.394 | 0.228 | 0.761 | 0.634 | - |
| uc.70+A | -2.073 | -0.398 | 0.192 | 0.759 | 0.000 | - |
| uc.229+ | -1.512 | -0.407 | 0.269 | 0.754 | 2.562 | - |
| uc.442+A | -1.974 | -0.417 | 0.211 | 0.749 | 0.000 | - |
| uc.57+ | -1.676 | -0.417 | 0.249 | 0.749 | 1.038 | - |
| uc.298+ | -1.920 | -0.420 | 0.219 | 0.747 | 0.000 | - |
| uc.214+ | -1.578 | -0.434 | 0.275 | 0.740 | 1.896 | - |
| uc.114+ | -2.475 | -0.436 | 0.176 | 0.739 | 0.000 | - |
| uc.186+ | -1.579 | -0.444 | 0.281 | 0.735 | 1.896 | - |
| uc.66+A | -2.368 | -0.446 | 0.188 | 0.734 | 0.000 | - |
| uc.290+A | -1.622 | -0.449 | 0.277 | 0.732 | 1.038 | - |
| uc.450+ | -1.844 | -0.451 | 0.245 | 0.731 | 0.634 | - |
| uc.54+A | -2.099 | -0.468 | 0.223 | 0.723 | 0.000 | - |
| uc.406+ | -1.665 | -0.469 | 0.281 | 0.723 | 1.038 | - |
| uc.244+ | -1.542 | -0.467 | 0.303 | 0.723 | 1.896 | - |
| uc.364+A | -1.946 | -0.470 | 0.241 | 0.722 | 0.000 | - |
| uc.318+A | -1.899 | -0.474 | 0.250 | 0.720 | 0.000 | - |
| uc.56+A | -1.755 | -0.473 | 0.270 | 0.720 | 0.634 | - |
| uc.270+ | -1.686 | -0.479 | 0.284 | 0.718 | 1.038 | - |
| uc.266+A | -1.642 | -0.479 | 0.292 | 0.717 | 1.038 | - |
| uc.294+A | -1.521 | -0.483 | 0.318 | 0.715 | 2.562 | - |
| uc.46+A | -1.945 | -0.487 | 0.251 | 0.713 | 0.000 | - |
| uc.122+ | -1.537 | -0.487 | 0.317 | 0.713 | 1.896 | - |
| uc.410+ | -2.204 | -0.490 | 0.222 | 0.712 | 0.000 | - |
| uc.186+A | -1.528 | -0.492 | 0.322 | 0.711 | 2.562 | - |
| uc.34+A | -1.990 | -0.496 | 0.249 | 0.709 | 0.000 | - |
| uc.53+ | -2.215 | -0.499 | 0.225 | 0.708 | 0.000 | - |
| uc.289+A | -2.106 | -0.505 | 0.240 | 0.705 | 0.000 | - |
| uc.150+ | -1.622 | -0.513 | 0.316 | 0.701 | 1.038 | - |
| uc.274+ | -2.222 | -0.519 | 0.233 | 0.698 | 0.000 | - |
| uc.457+A | -2.010 | -0.524 | 0.261 | 0.696 | 0.000 | - |
| uc.202+A | -2.575 | -0.535 | 0.208 | 0.690 | 0.000 | - |
| uc.298+A | -1.513 | -0.536 | 0.354 | 0.690 | 2.562 | - |
| uc.46+ | -1.772 | -0.547 | 0.309 | 0.685 | 0.634 | - |
| uc.58+ | -1.563 | -0.547 | 0.350 | 0.684 | 1.896 | - |
| uc.420+A | -1.651 | -0.551 | 0.334 | 0.682 | 1.038 | - |
| uc.302+A | -1.575 | -0.552 | 0.350 | 0.682 | 1.896 | - |
| uc.354+A | -1.541 | -0.552 | 0.358 | 0.682 | 1.896 | - |
| uc.310+A | -2.402 | -0.563 | 0.234 | 0.677 | 0.000 | - |
| uc.158+A | -1.814 | -0.563 | 0.311 | 0.677 | 0.634 | - |
| uc.178+ | -2.023 | -0.566 | 0.280 | 0.676 | 0.000 | - |
| uc.62+ | -2.438 | -0.567 | 0.232 | 0.675 | 0.000 | - |
| uc.406+A | -2.520 | -0.570 | 0.226 | 0.674 | 0.000 | - |
| uc.313+A | -1.715 | -0.571 | 0.333 | 0.673 | 0.634 | - |
| uc.390+A | -2.349 | -0.577 | 0.246 | 0.670 | 0.000 | - |
| uc.382+A | -1.959 | -0.577 | 0.295 | 0.670 | 0.000 | - |
| uc.4+A | -2.408 | -0.583 | 0.242 | 0.668 | 0.000 | - |
| uc.221+A | -1.765 | -0.590 | 0.334 | 0.664 | 0.634 | - |
| uc.249+A | -1.829 | -0.593 | 0.324 | 0.663 | 0.634 | - |
| uc.170+A | -2.868 | -0.596 | 0.208 | 0.661 | 0.000 | - |
| uc.133+ | -1.843 | -0.599 | 0.325 | 0.660 | 0.634 | - |
| uc.138+ | -1.733 | -0.604 | 0.349 | 0.658 | 0.634 | - |
| uc.286+A | -1.751 | -0.609 | 0.348 | 0.656 | 0.634 | - |
| uc.334+A | -2.072 | -0.613 | 0.296 | 0.654 | 0.000 | - |
| uc.253+ | -2.143 | -0.618 | 0.288 | 0.652 | 0.000 | - |
| uc.90+A | -2.426 | -0.620 | 0.256 | 0.651 | 0.000 | - |
| uc.465+A | -1.987 | -0.619 | 0.312 | 0.651 | 0.000 | - |
| uc.174+ | -3.224 | -0.623 | 0.193 | 0.649 | 0.000 | - |
| uc.50+A | -2.307 | -0.623 | 0.270 | 0.649 | 0.000 | - |
| uc.100+ | -1.679 | -0.623 | 0.371 | 0.649 | 1.038 | - |
| uc.396+A | -2.307 | -0.628 | 0.272 | 0.647 | 0.000 | - |
| uc.418+ | -2.272 | -0.632 | 0.278 | 0.645 | 0.000 | - |
| uc.161+ | -2.064 | -0.637 | 0.308 | 0.643 | 0.000 | - |
| uc.182+ | -1.741 | -0.636 | 0.365 | 0.643 | 0.634 | - |
| uc.422+ | -1.835 | -0.655 | 0.357 | 0.635 | 0.634 | - |
| uc.21+ | -2.863 | -0.658 | 0.230 | 0.634 | 0.000 | - |
| uc.165+ | -2.509 | -0.659 | 0.263 | 0.633 | 0.000 | - |
| uc.270+A | -2.921 | -0.668 | 0.229 | 0.630 | 0.000 | - |
| uc.402+A | -2.212 | -0.666 | 0.301 | 0.630 | 0.000 | - |
| uc.158+ | -1.909 | -0.677 | 0.355 | 0.625 | 0.000 | - |
| uc.318+ | -3.037 | -0.680 | 0.224 | 0.624 | 0.000 | - |
| uc.462+ | -1.730 | -0.689 | 0.399 | 0.620 | 0.634 | - |
| uc.38+A | -1.905 | -0.691 | 0.363 | 0.619 | 0.000 | - |
| uc.230+A | -2.817 | -0.699 | 0.248 | 0.616 | 0.000 | - |
| uc.410+A | -2.643 | -0.700 | 0.265 | 0.616 | 0.000 | - |
| uc.329+A | -3.249 | -0.705 | 0.217 | 0.614 | 0.000 | - |
| uc.29+ | -1.584 | -0.711 | 0.449 | 0.611 | 1.896 | - |
| uc.190+ | -2.062 | -0.715 | 0.347 | 0.609 | 0.000 | - |
| uc.342+ | -2.433 | -0.718 | 0.295 | 0.608 | 0.000 | - |
| uc.413+A | -1.946 | -0.717 | 0.369 | 0.608 | 0.000 | - |
| uc.142+ | -2.008 | -0.721 | 0.359 | 0.607 | 0.000 | - |
| uc.354+ | -3.632 | -0.726 | 0.200 | 0.605 | 0.000 | - |
| uc.422+A | -3.129 | -0.733 | 0.234 | 0.602 | 0.000 | - |
| uc.390+ | -1.613 | -0.743 | 0.461 | 0.597 | 1.038 | - |
| uc.341+ | -1.926 | -0.750 | 0.390 | 0.594 | 0.000 | - |
| uc.277+A | -2.421 | -0.763 | 0.315 | 0.589 | 0.000 | - |
| uc.278+A | -2.699 | -0.765 | 0.284 | 0.588 | 0.000 | - |
| uc.206+A | -3.170 | -0.777 | 0.245 | 0.584 | 0.000 | - |
| uc.170+ | -1.759 | -0.781 | 0.444 | 0.582 | 0.634 | - |
| uc.433+ | -2.437 | -0.790 | 0.324 | 0.578 | 0.000 | - |
| uc.338+A | -1.903 | -0.796 | 0.418 | 0.576 | 0.000 | - |
| uc.128+A | -1.960 | -0.799 | 0.408 | 0.575 | 0.000 | - |
| uc.77+ | -2.503 | -0.803 | 0.321 | 0.573 | 0.000 | - |
| uc.359+A | -2.265 | -0.820 | 0.362 | 0.567 | 0.000 | - |
| uc.234+A | -3.223 | -0.821 | 0.255 | 0.566 | 0.000 | - |
| uc.134+ | -1.957 | -0.833 | 0.426 | 0.561 | 0.000 | - |
| uc.206+ | -3.030 | -0.852 | 0.281 | 0.554 | 0.000 | - |
| uc.398+A | -3.323 | -0.878 | 0.264 | 0.544 | 0.000 | - |
| uc.167+ | -2.265 | -0.884 | 0.390 | 0.542 | 0.000 | - |
| uc.177+ | -2.022 | -0.888 | 0.439 | 0.540 | 0.000 | - |
| uc.350+ | -3.876 | -0.900 | 0.232 | 0.536 | 0.000 | - |
| uc.326+ | -2.431 | -0.908 | 0.374 | 0.533 | 0.000 | - |
| uc.110+A | -2.391 | -0.909 | 0.380 | 0.533 | 0.000 | - |
| uc.126+A | -2.781 | -0.940 | 0.338 | 0.521 | 0.000 | - |
| uc.417+A | -1.824 | -0.940 | 0.516 | 0.521 | 0.634 | - |
| uc.82+ | -2.421 | -0.978 | 0.404 | 0.508 | 0.000 | - |
| uc.278+ | -2.637 | -0.984 | 0.373 | 0.506 | 0.000 | - |
| uc.309+A | -3.100 | -0.986 | 0.318 | 0.505 | 0.000 | - |
| uc.269+A | -1.982 | -0.995 | 0.502 | 0.502 | 0.000 | - |
| uc.160+ | -2.851 | -1.003 | 0.352 | 0.499 | 0.000 | - |
| uc.470+A | -3.580 | -1.041 | 0.291 | 0.486 | 0.000 | - |
| uc.249+ | -3.871 | -1.105 | 0.285 | 0.465 | 0.000 | - |
| uc.345+ | -2.229 | -1.122 | 0.503 | 0.459 | 0.000 | - |
| uc.466+A | -3.426 | -1.138 | 0.332 | 0.455 | 0.000 | - |
| uc.414+ | -3.046 | -1.144 | 0.376 | 0.452 | 0.000 | - |
| uc.382+ | -3.642 | -1.149 | 0.315 | 0.451 | 0.000 | - |
| uc.13+ | -2.137 | -1.155 | 0.541 | 0.449 | 0.000 | - |
| uc.84+A | -4.313 | -1.231 | 0.285 | 0.426 | 0.000 | - |
| uc.198+A | -3.051 | -1.233 | 0.404 | 0.425 | 0.000 | - |
| uc.282+A | -2.534 | -1.315 | 0.519 | 0.402 | 0.000 | - |
| uc.398+ | -3.511 | -1.321 | 0.376 | 0.400 | 0.000 | - |
| uc.346+ | -4.030 | -1.617 | 0.401 | 0.326 | 0.000 | - |
| uc.78+ | -3.466 | -1.650 | 0.476 | 0.319 | 0.000 | - |
| uc.8+ | -2.959 | -1.913 | 0.646 | 0.266 | 0.000 | - |

^1^Score (d) Numerator/Denominator: It is equivalent to a T test statistics.

^2^Numerator (r): Difference of the sample means in the two classes (BlCa and PBlCa). It is equivalent to the numerator of the independent samples T-test statistics.

^3^Denominator (s + s0): Estimate of the standard error of the difference in means. It is obtained summing the usual denominator of the independent samples T-test statistics (s) plus an an exchangeability constant shrinking the scores of genes with expression near 0.

*Clinical characteristics listed in Table 1, data set 2.

Abbreviations: uc, ultraconserved RNA.

**Supplementary Table S3. Genomic features of transcribed ultraconserved regions (T-UCRs).**

| **chr** | **uc_star_**  **bp** | **uc_end_**  **bp** | **uc_**  **name** | **type** | **feature_**  **type** | **uc_host_transcript** | **Enhancer** | **UTR** |
| --- | --- | --- | --- | --- | --- | --- | --- | --- |
| 1 | 10597697 | 10597903 | 1 | S | intron | PEX14-001,PEX14-004 | na | na |
| 1 | 10965574 | 10965848 | 10 | S | intergene | na | na | na |
| 2 | 174114780 | 174114986 | 100 | S | intron | AC013461 1-001,AC013461 1-003,MLK7-AS1,MLK7-AS1-001,MLK7-AS1-003 | na | na |
| 2 | 174774481 | 174774734 | 101 | S | exon | SP3-001,SP3-002,SP3-003,SP3-004,SP3-201 | na | yes |
| 2 | 174946409 | 174946746 | 102 | M | exon_intron | OLA1-001,OLA1-002,OLA1-003,OLA1-005,OLA1-006,OLA1-008,OLA1-009 | na | na |
| 2 | 174969672 | 174969904 | 103 | S | intron | OLA1-001,OLA1-002,OLA1-003,OLA1-005,OLA1-006,OLA1-008,OLA1-009 | na | na |
| 2 | 174986934 | 174987149 | 104 | S | intron | OLA1-001,OLA1-002,OLA1-003,OLA1-005,OLA1-006,OLA1-008,OLA1-009 | na | na |
| 2 | 174989787 | 174990009 | 105 | S | intron | OLA1-001,OLA1-002,OLA1-003,OLA1-005,OLA1-009 | na | na |
| 2 | 175025423 | 175025628 | 106 | S | intron | OLA1-001,OLA1-002,OLA1-003,OLA1-005,OLA1-009 | na | na |
| 2 | 175207608 | 175207844 | 107 | S | downstream | na | na | na |
| 2 | 176940357 | 176940730 | 108 | S | downstream | na | na | na |
| 2 | 177503338 | 177503561 | 109 | M | intron,  upstream | AC017048 4-001 | na | na |
| 2 | 237071382 | 237071624 | 110 | M | downstream, upstream | na | na | na |
| 3 | 9471461 | 9471756 | 111 | M | exon_intron,  intron | SETD5-001,SETD5-002,SETD5-003,SETD5-004,SETD5-007,SETD5-009,SETD5-010,SETD5-020,SETD5-021,SETD5-023 | na | yes |
| 3 | 18169564 | 18169909 | 112 | S | intron | TBC1D5-003 | na | na |
| 3 | 18676404 | 18676650 | 113 | S | intergene | na | na | na |
| 3 | 18844450 | 18844743 | 114 | S | intron | AC105750 1-002 | na | na |
| 3 | 19034158 | 19034376 | 115 | S | intergene | na | na | na |
| 3 | 70566424 | 70566629 | 116 | S | intergene | na | na | na |
| 3 | 70871840 | 70872090 | 117 | S | intergene | na | na | na |
| 3 | 70872092 | 70872310 | 118 | S | intergene | na | na | na |
| 3 | 114433467 | 114433767 | 119 | S | intron | ZBTB20-002,ZBTB20-004,ZBTB20-005,ZBTB20-006,ZBTB20-008,ZBTB20-010,ZBTB20-013,ZBTB20-014,ZBTB20-015,ZBTB20-017 | na | na |
| 1 | 35650227 | 35650427 | 12 | S | intron | SFPQ-001,SFPQ-002,SFPQ-003 | na | na |
| 3 | 114435039 | 114435308 | 120 | S | intron | ZBTB20-002,ZBTB20-004,ZBTB20-005,ZBTB20-006,ZBTB20-008,ZBTB20-010,ZBTB20-013,ZBTB20-014,ZBTB20-015,ZBTB20-017 | na | na |
| 3 | 114575474 | 114575766 | 121 | S | intron | ZBTB20-005,ZBTB20-006,ZBTB20-008,ZBTB20-010,ZBTB20-013,ZBTB20-014,ZBTB20-015,ZBTB20-017 | na | na |
| 3 | 114611891 | 114612105 | 122 | S | intron | ZBTB20-005,ZBTB20-006,ZBTB20-008,ZBTB20-010,ZBTB20-013,ZBTB20-015,ZBTB20-017 | na | na |
| 3 | 136983544 | 136984035 | 123 | S | intergene | na | na | na |
| 3 | 137048444 | 137048730 | 124 | S | intergene | na | yes | na |
| 3 | 137068317 | 137068581 | 125 | S | intergene | na | na | na |
| 3 | 137125648 | 137125918 | 126 | S | intergene | na | na | na |
| 3 | 147030708 | 147030979 | 127 | S | intron | RP11-649A16 1,RP11-649A16 1-001 | na | na |
| 3 | 147049638 | 147049936 | 128 | S | intron | RP11-649A16 1,RP11-649A16 1-001 | na | na |
| 3 | 152164387 | 152164598 | 129 | M | intron,  exon_intron,  upstream | MBNL1-001,MBNL1-003,MBNL1-004,MBNL1-006,MBNL1-007,MBNL1-008,MBNL1-009,MBNL1-010,MBNL1-011,MBNL1-012,MBNL1-013,MBNL1-019,MBNL1-201 | na | na |
| 1 | 36359190 | 36359426 | 13 | S | exon_intron | EIF2C1-001,EIF2C1-002 | na | na |
| 3 | 157776574 | 157776797 | 130 | S | intron | RP11-290K4 2,RP11-290K4 2-001,RP11-290K4 2-002 | na | na |
| 3 | 157990040 | 157990246 | 131 | M | downstream,  intron | RSRC1-001,RSRC1-002,RSRC1-003,RSRC1-004,RSRC1-005,RSRC1-006,RSRC1-007,RSRC1-010,RSRC1-011,RSRC1-012,RSRC1-013,RSRC1-014 | na | na |
| 3 | 158026159 | 158026366 | 132 | S | intron | RSRC1-001,RSRC1-002,RSRC1-003,RSRC1-004,RSRC1-005,RSRC1-006,RSRC1-007,RSRC1-010,RSRC1-011,RSRC1-012,RSRC1-013 | na | na |
| 3 | 158026478 | 158026754 | 133 | S | intron | RSRC1-001,RSRC1-002,RSRC1-003,RSRC1-004,RSRC1-005,RSRC1-006,RSRC1-007,RSRC1-010,RSRC1-011,RSRC1-012,RSRC1-013 | na | na |
| 3 | 158245904 | 158246114 | 134 | S | intron | RSRC1-001,RSRC1-002,RSRC1-004,RSRC1-006,RSRC1-007,RSRC1-012,RSRC1-013 | na | na |
| 3 | 168834283 | 168834483 | 135 | M | exon,intron | MECOM-004,MECOM-015,MECOM-016,MECOM-017,MECOM-018,MECOM-019,MECOM-020,MECOM-201,MECOM-202 | na | na |
| 3 | 169193952 | 169194298 | 136 | S | intron | MECOM-001,MECOM-004,MECOM-005,RP11-641D5 2,RP11-641D5 2-001 | na | na |
| 3 | 180436857 | 180437241 | 137 | S | intron | CCDC39-002,CCDC39-003,CCDC39-004,CCDC39-005 | na | na |
| 3 | 185649296 | 185649714 | 138 | M | exon_intron, intron | TRA2B-001,TRA2B-003,TRA2B-004,TRA2B-008,TRA2B-009,TRA2B-010,TRA2B-011,TRA2B-012,TRA2B-013,TRA2B-017 | na | yes |
| 4 | 4523440 | 4523777 | 139 | S | intron | STX18-001,STX18-002,STX18-004,STX18-005 | na | na |
| 1 | 38495004 | 38495216 | 14 | S | downstream | na | na | na |
| 4 | 13009870 | 13010092 | 140 | S | intergene | na | na | na |
| 4 | 24529162 | 24529456 | 141 | M | exon,intron | DHX15-001,DHX15-002,DHX15-003,DHX15-004,DHX15-008,DHX15-009,DHX15-010 | na | yes |
| 4 | 41750069 | 41750327 | 142 | M | exon,intron, upstream | PHOX2B-001,RP11-227F19 1-001 | na | na |
| 4 | 76579124 | 76579341 | 143 | M | exon_intron, intron | G3BP2-001,G3BP2-002,G3BP2-003,MT1P2-001 | na | na |
| 4 | 83346665 | 83346869 | 144 | M | exon,intron, exon_intron, upstream | HNRPDL-001,HNRPDL-002,HNRPDL-003,HNRPDL-004,HNRPDL-005,MT1P2-001 | na | yes |
| 4 | 105346313 | 105346560 | 145 | S | intron | MT1P2-001 | na | na |
| 4 | 111916476 | 111916689 | 146 | S | intron | MT1P2-001 | na | na |
| 4 | 151236383 | 151236690 | 147 | M | exon,intron | LRBA-001,LRBA-002,LRBA-003,LRBA-004,LRBA-008,LRBA-010,MT1P2-001 | na | yes |
| 4 | 151493952 | 151494191 | 148 | S | intron | LRBA-001,LRBA-002,LRBA-003,LRBA-004,LRBA-008,LRBA-013,MT1P2-001 | na | na |
| 4 | 151494193 | 151494396 | 149 | S | intron | LRBA-001,LRBA-002,LRBA-003,LRBA-004,LRBA-008,LRBA-013,MT1P2-001 | na | na |
| 1 | 38561076 | 38561308 | 15 | S | intron | RP5-884C9 2,RP5-884C9 2-001,RP5-884C9 2-002 | na | na |
| 5 | 3512621 | 3512882 | 150 | S | intron | RP11-121L11 1,RP11-121L11 1-001 | na | na |
| 5 | 32380137 | 32380350 | 151 | M | exon,  exon_intron,  intron,  upstream | ZFR-001,ZFR-004,ZFR-005,ZFR-006 | na | na |
| 5 | 50335638 | 50335838 | 152 | S | intergene | na | na | na |
| 5 | 72195686 | 72195925 | 153 | M | exon,  exon_intron | TNPO1-001,TNPO1-006,TNPO1-013,TNPO1-014,TNPO1-015,TNPO1-202 | na | na |
| 5 | 72210015 | 72210217 | 154 | S | exon | TNPO1-001,TNPO1-202 | na | yes |
| 5 | 76934364 | 76934570 | 155 | S | exon | OTP-001 | na | yes |
| 5 | 76935419 | 76935631 | 156 | M | exon, upstream | OTP-001 | na | yes |
| 5 | 76941161 | 76941367 | 157 | S | intergene | na | na | na |
| 5 | 77140253 | 77140476 | 158 | S | intron | TBCA-005 | na | na |
| 5 | 77147932 | 77148403 | 159 | S | intron | TBCA-005 | na | na |
| 1 | 38628199 | 38628409 | 16 | S | intergene | na | na | na |
| 5 | 77268844 | 77269165 | 160 | S | intergene | na | yes | na |
| 5 | 77358529 | 77358806 | 161 | S | intron | AP3B1-001,AP3B1-002 | na | na |
| 5 | 81147361 | 81147578 | 162 | M | exon, upstream | CTD-2249K22 1-001 | na | na |
| 5 | 87168623 | 87168998 | 163 | S | intergene | na | na | na |
| 5 | 87240405 | 87240607 | 164 | S | intergene | na | na | na |
| 5 | 87692933 | 87693156 | 165 | S | intron | CTC-358I24 1,CTC-358I24 1-001,CTC-358I24 1-003,CTC-358I24 1-004,CTC-358I24 1-005,CTC-358I24 1-008 | na | na |
| 5 | 87961805 | 87962114 | 166 | M | downstream, exon,intron | LINC00461-001,LINC00461-003,LINC00461-005,LINC00461-007,LINC00461-009,LINC00461-010,LINC00461-013,LINC00461-014 | na | na |
| 5 | 88179624 | 88179824 | 167 | S | intron | CTC-454M9 1,CTC-454M9 1-004,CTC-454M9 1-005,CTC-454M9 1-006,MEF2C-004,MEF2C-005,MEF2C-006,MEF2C-007,MEF2C-010 | na | na |
| 5 | 90928793 | 90929012 | 168 | S | intergene | na | na | na |
| 5 | 92921017 | 92921220 | 169 | M | exon_intron, intron | NR2F1-001,RP11-65F13 2,RP11-65F13 2-001 | na | na |
| 1 | 38802151 | 38802387 | 17 | S | intergene | na | na | na |
| 5 | 93227670 | 93227979 | 170 | S | intron | FAM172A-001,FAM172A-002,FAM172A-004,FAM172A-005,FAM172A-006,FAM172A-007 | na | na |
| 5 | 93575847 | 93576054 | 171 | S | intron | KIAA0825-001 | na | na |
| 5 | 93650719 | 93650936 | 172 | S | intron | KIAA0825-001 | na | na |
| 5 | 133726160 | 133726435 | 173 | S | exon | UBE2B-001,UBE2B-006,UBE2B-007,UBE2B-008 | na | yes |
| 5 | 138643654 | 138643913 | 174 | M | exon,intron | MATR3-001,MATR3-002,MATR3-003,MATR3-007,MATR3-008,MATR3-009,MATR3-010,MATR3-011,MATR3-012,MATR3-013,MATR3-018,MATR3-019,MATR3-025,MATR3-028,MATR3-029,MATR3-030,MATR3-031,MATR3-033,MATR3-034,MATR3-035,MATR3-036,MATR3-037,MATR3-039,MATR3-201 | na | na |
| 5 | 158341838 | 158342087 | 175 | S | intron | EBF1-001,EBF1-002,EBF1-003,EBF1-005,EBF1-006,EBF1-008,EBF1-009,EBF1-010 | na | na |
| 5 | 167332695 | 167332940 | 176 | S | intron | ODZ2-001,ODZ2-003,ODZ2-004,ODZ2-009,ODZ2-010 | na | na |
| 5 | 170417629 | 170417885 | 177 | S | intron | RANBP17-001,RANBP17-002,RANBP17-003,RANBP17-005,RANBP17-007,RANBP17-008,RANBP17-009,RANBP17-015 | na | na |
| 5 | 170417998 | 170418246 | 178 | S | intron | RANBP17-001,RANBP17-002,RANBP17-003,RANBP17-005,RANBP17-007,RANBP17-008,RANBP17-009,RANBP17-015 | na | na |
| 5 | 170628212 | 170628430 | 179 | S | intron | RANBP17-001,RANBP17-002,RANBP17-003,RANBP17-005,RANBP17-007,RANBP17-008,RANBP17-009,RANBP17-010,RANBP17-016,RANBP17-017,RANBP17-018 | na | na |
| 1 | 44715661 | 44715898 | 18 | S | intron | ERI3-001,ERI3-003,ERI3-004,ERI3-006,ERI3-009 | na | na |
| 5 | 170628489 | 170628713 | 180 | S | intron | RANBP17-001,RANBP17-002,RANBP17-003,RANBP17-005,RANBP17-007,RANBP17-008,RANBP17-009,RANBP17-010,RANBP17-016,RANBP17-017,RANBP17-018 | na | na |
| 5 | 170629479 | 170629756 | 181 | S | intron | RANBP17-001,RANBP17-002,RANBP17-003,RANBP17-005,RANBP17-007,RANBP17-008,RANBP17-009,RANBP17-010,RANBP17-016,RANBP17-017,RANBP17-018 | na | na |
| 5 | 170703079 | 170703317 | 182 | S | intron | RANBP17-001,RANBP17-005,RANBP17-007,RANBP17-008,RANBP17-009,RANBP17-010 | na | na |
| 5 | 171384520 | 171384755 | 183 | M | exon_intron, intron | FBXW11-001,FBXW11-002,FBXW11-003,FBXW11-004,FBXW11-005,FBXW11-006,FBXW11-007,FBXW11-008,FBXW11-011 | na | yes |
| 5 | 173385292 | 173385521 | 184 | S | exon | CPEB4-001,CPEB4-002 | na | yes |
| 5 | 178044307 | 178044717 | 185 | M | downstream, exon,  exon_intron,  intron | CLK4-001,CLK4-002,CLK4-003,CLK4-005,CLK4-006,CLK4-008,CLK4-009,CLK4-011,CLK4-013 | na | yes |
| 5 | 179046197 | 179046501 | 186 | M | exon,intron | HNRNPH1-001,HNRNPH1-002,HNRNPH1-003,HNRNPH1-005,HNRNPH1-006,HNRNPH1-007,HNRNPH1-008,HNRNPH1-009,HNRNPH1-010,HNRNPH1-013,HNRNPH1-015,HNRNPH1-021,HNRNPH1-022,HNRNPH1-025,HNRNPH1-026,HNRNPH1-027,HNRNPH1-028,HNRNPH1-036,HNRNPH1-037,HNRNPH1-039,HNRNPH1-040,HNRNPH1-045,HNRNPH1-046,HNRNPH1-048,HNRNPH1-049,HNRNPH1-050 | na | na |
| 6 | 10394677 | 10394888 | 187 | S | intron | TFAP2A-017 | na | na |
| 6 | 16299384 | 16299598 | 188 | M | downstream, exon | ATXN1-001,ATXN1-201 | na | yes |
| 6 | 36567517 | 36568089 | 189 | M | exon_intron, intron | SRSF3-001,SRSF3-002,SRSF3-201,T-001 | na | yes |
| 1 | 44990312 | 44990567 | 19 | S | intron | RNF220-001,RNF220-002 | na | na |
| 6 | 41523440 | 41523639 | 190 | S | intron | FOXP4-001,FOXP4-003,FOXP4-006,T-001 | na | na |
| 6 | 51076794 | 51077001 | 191 | S | intron | T-001 | na | na |
| 6 | 51148997 | 51149240 | 192 | S | intron | T-001 | na | na |
| 6 | 86321686 | 86322004 | 193 | M | exon,intron | SYNCRIP-002,SYNCRIP-004,T-001 | na | yes |
| 6 | 93969064 | 93969264 | 194 | M | exon_intron, intron | EPHA7-001,T-001 | na | na |
| 6 | 97663356 | 97663628 | 195 | S | intron | MMS22L-001,MMS22L-010,T-001 | na | na |
| 6 | 98116540 | 98116760 | 196 | S | intron | T-001 | yes | na |
| 6 | 98362799 | 98363022 | 197 | S | intron | T-001 | na | na |
| 6 | 98719889 | 98720195 | 198 | M | intron, upstream | T-001 | na | na |
| 6 | 98813855 | 98814110 | 199 | S | intron | T-001 | na | na |
| 1 | 10732543 | 10732749 | 2 | S | intron | CASZ1-001,CASZ1-003,CASZ1-006,CASZ1-007 | na | na |
| 1 | 45002372 | 45002640 | 20 | S | intron | RNF220-001,RNF220-002 | na | na |
| 6 | 98995533 | 98995786 | 200 | S | intron | T-001 | na | na |
| 6 | 100051984 | 100052223 | 201 | M | intron, upstream | T-001 | na | na |
| 6 | 100973983 | 100974212 | 202 | M | intron, upstream | ASCC3-001,T-001 | na | na |
| 6 | 163991704 | 163991906 | 203 | M | exon,  exon_intron, intron | QKI-001,QKI-006,QKI-010,QKI-011,QKI-017,QKI-019,T-001 | na | yes |
| 7 | 1265904 | 1266105 | 204 | S | intergene | na | na | na |
| 7 | 20829789 | 20830040 | 205 | S | upstream | na | na | na |
| 7 | 21003765 | 21004263 | 206 | S | intron | AC006481 1-001 | na | na |
| 7 | 21811493 | 21811722 | 207 | S | intron | DNAH11-001 | na | na |
| 7 | 23561670 | 23561887 | 208 | M | exon,  exon_intron,  intron | TRA2A-001,TRA2A-002,TRA2A-003,TRA2A-004,TRA2A-005,TRA2A-201,TRA2A-202 | na | yes |
| 7 | 23561888 | 23562137 | 209 | M | exon,  exon_intron,  intron | TRA2A-001,TRA2A-002,TRA2A-003,TRA2A-004,TRA2A-005,TRA2A-201,TRA2A-202 | na | yes |
| 1 | 49112986 | 49113220 | 21 | S | intron | AGBL4-001,AGBL4-002,AGBL4-004 | na | na |
| 7 | 26697078 | 26697334 | 210 | S | intergene | na | na | na |
| 7 | 26729472 | 26729762 | 211 | S | intron | SKAP2-001 | na | na |
| 7 | 27141938 | 27142142 | 212 | M | downstream, exon | HOXA2-001 | na | yes |
| 7 | 27183132 | 27183332 | 213 | M | downstream, exon,intron, upstream | HOXA-AS3,HOXA-AS3-003,HOXA-AS3-004,HOXA3-008,HOXA3-009,HOXA5-001 | na | yes |
| 7 | 31402398 | 31402640 | 214 | S | intergene | na | na | na |
| 7 | 42192585 | 42192846 | 215 | S | intron | GLI3-001,GLI3-005 | na | na |
| 7 | 50358155 | 50358466 | 216 | S | intron | IKZF1-001,IKZF1-002,IKZF1-003,IKZF1-004,IKZF1-007,IKZF1-008,IKZF1-009,IKZF1-011,IKZF1-201 | na | na |
| 7 | 54636656 | 54636876 | 217 | M | exon_intron, intron | GS1-18A18 1,GS1-18A18 1-001,VSTM2A-002,VSTM2A-003,VSTM2A-004,VSTM2A-006,VSTM2A-007 | na | yes |
| 7 | 69802975 | 69803260 | 218 | S | intron | AUTS2-001,AUTS2-002,AUTS2-004 | na | na |
| 7 | 69980780 | 69980989 | 219 | S | intron | AUTS2-001,AUTS2-002 | na | na |
| 1 | 51006231 | 51006538 | 22 | S | intron | FAF1-001,FAF1-002,FAF1-003,FAF1-005 | na | na |
| 7 | 96633916 | 96634172 | 220 | M | downstream, intron, upstream | DLX6-AS1,DLX6-AS1-001,DLX6-AS1-002,DLX6-AS1-004,DLX6-AS1-005,DLX6-AS1-009,DLX6-AS1-010 | na | na |
| 7 | 96641301 | 96641649 | 221 | M | downstream, exon,intron, upstream | DLX6-AS1,DLX6-AS1-001,DLX6-AS1-002,DLX6-AS1-004,DLX6-AS1-010 | na | na |
| 7 | 114057171 | 114057371 | 222 | S | intron | AC073626 2-001,FOXP2-001,FOXP2-002,FOXP2-003,FOXP2-004,FOXP2-005,FOXP2-006,FOXP2-007,FOXP2-008,FOXP2-009,FOXP2-010,FOXP2-011,FOXP2-014,FOXP2-020 | na | na |
| 7 | 114058185 | 114058452 | 223 | S | intron | AC073626 2-001,FOXP2-001,FOXP2-002,FOXP2-003,FOXP2-004,FOXP2-005,FOXP2-006,FOXP2-007,FOXP2-008,FOXP2-009,FOXP2-010,FOXP2-011,FOXP2-014,FOXP2-020 | na | na |
| 7 | 114063019 | 114063313 | 224 | M | intron, upstream | FOXP2-001,FOXP2-002,FOXP2-003,FOXP2-004,FOXP2-005,FOXP2-006,FOXP2-007,FOXP2-008,FOXP2-009,FOXP2-010,FOXP2-011,FOXP2-014,FOXP2-020 | na | na |
| 7 | 114072855 | 114073055 | 225 | S | intron | FOXP2-001,FOXP2-002,FOXP2-003,FOXP2-004,FOXP2-005,FOXP2-006,FOXP2-007,FOXP2-008,FOXP2-009,FOXP2-010,FOXP2-014,FOXP2-015,FOXP2-016,FOXP2-017,FOXP2-018,FOXP2-020 | na | na |
| 7 | 114209318 | 114209522 | 226 | S | intron | FOXP2-001,FOXP2-002,FOXP2-003,FOXP2-004,FOXP2-005,FOXP2-006,FOXP2-008,FOXP2-009,FOXP2-010,FOXP2-014,FOXP2-015,FOXP2-016,FOXP2-017,FOXP2-018,FOXP2-020 | na | na |
| 7 | 114295316 | 114295546 | 227 | S | intron | FOXP2-001,FOXP2-004,FOXP2-005,FOXP2-006,FOXP2-008,FOXP2-014,FOXP2-020 | na | na |
| 7 | 115116697 | 115116961 | 228 | S | intergene | na | yes | na |
| 7 | 115134645 | 115134940 | 229 | S | intergene | na | na | na |
| 1 | 51035777 | 51036005 | 23 | S | intron | FAF1-001,FAF1-002,FAF1-003,FAF1-005 | na | na |
| 7 | 115319461 | 115319698 | 230 | M | exon, upstream | RP11-458K10 2-001 | na | na |
| 7 | 115582117 | 115582340 | 231 | S | intron | TFEC-001,TFEC-002,TFEC-006,TFEC-007,TFEC-008 | na | na |
| 7 | 121944606 | 121944852 | 232 | M | exon_intron, intron | FEZF1-003,FEZF1-AS1-001,RP11-560I19 4,RP11-560I19 4-001 | na | na |
| 7 | 150828314 | 150828579 | 233 | M | exon,intron | AGAP3-001,AGAP3-002,AGAP3-021,AGAP3-022,AGAP3-023,AGAP3-024 | na | na |
| 7 | 156812385 | 156812656 | 234 | S | downstream | na | na | na |
| 8 | 25775921 | 25776147 | 235 | S | intron | EBF2-001,EBF2-002 | na | na |
| 8 | 37250469 | 37250735 | 236 | S | intergene | na | na | na |
| 8 | 53137902 | 53138369 | 237 | S | intron | ST18-001,ST18-002,ST18-005,ST18-013,ST18-014,ST18-015,ST18-016,ST18-017 | na | na |
| 8 | 53167117 | 53167474 | 238 | S | intron | ST18-001,ST18-002,ST18-005,ST18-021 | na | na |
| 8 | 59942373 | 59942672 | 239 | S | intron | TOX-001 | na | na |
| 1 | 51099145 | 51099480 | 24 | S | intron | FAF1-001,FAF1-002,FAF1-003 | na | na |
| 8 | 65492539 | 65492744 | 240 | M | intron, upstream | RP11-21C4 1,RP11-21C4 1-001 | na | na |
| 8 | 65497130 | 65497331 | 241 | M | downstream, upstream | na | na | na |
| 8 | 66149297 | 66149561 | 242 | S | intergene | na | na | na |
| 8 | 77690962 | 77691177 | 243 | S | intron | ZFHX4-002,ZFHX4-005,ZFHX4-010,ZFHX4-011,ZFHX4-013 | na | na |
| 8 | 105962349 | 105962669 | 244 | S | intron | RP11-127H5 1,RP11-127H5 1-002 | na | na |
| 8 | 106333832 | 106334170 | 245 | S | intron | RP11-127H5 1,RP11-127H5 1-002,ZFPM2-001,ZFPM2-002 | na | na |
| 8 | 119123218 | 119123501 | 246 | S | exon | EXT1-001,EXT1-004 | na | yes |
| 9 | 969154 | 969514 | 247 | S | downstream | na | na | na |
| 9 | 974189 | 974410 | 248 | S | upstream | na | na | na |
| 9 | 8095728 | 8095987 | 249 | S | intergene | na | na | na |
| 1 | 51166034 | 51166268 | 25 | S | intron | FAF1-001,FAF1-002,FAF1-003 | na | na |
| 9 | 13939910 | 13940118 | 250 | S | intron | C9orf146-001 | na | na |
| 9 | 15874309 | 15874521 | 251 | S | intron | C9orf93-001,C9orf93-003,C9orf93-005,C9orf93-006,C9orf93-008 | na | na |
| 9 | 16710753 | 16710983 | 252 | S | intron | BNC2-001,BNC2-002,BNC2-007,BNC2-008,BNC2-009,BNC2-010 | na | na |
| 9 | 17332212 | 17332433 | 253 | S | intron | CNTLN-001 | na | na |
| 9 | 23496725 | 23497003 | 254 | S | intergene | na | na | na |
| 9 | 23691768 | 23691999 | 255 | M | exon, upstream | ELAVL2-001,ELAVL2-002,ELAVL2-004,ELAVL2-201,ELAVL2-202 | na | yes |
| 9 | 23692234 | 23692439 | 256 | M | exon, upstream | ELAVL2-001,ELAVL2-002,ELAVL2-004,ELAVL2-201,ELAVL2-202 | na | yes |
| 9 | 37215204 | 37215467 | 257 | S | intron | ZCCHC7-002,ZCCHC7-003,ZCCHC7-004 | na | na |
| 9 | 37324424 | 37324624 | 258 | M | downstream, intron | ZCCHC7-002,ZCCHC7-003,ZCCHC7-004,ZCCHC7-006 | na | na |
| 9 | 79627879 | 79628186 | 259 | S | intergene | na | na | na |
| 1 | 63369646 | 63369857 | 26 | M | downstream, upstream | na | na | na |
| 9 | 81472424 | 81472654 | 260 | S | intergene | na | na | na |
| 9 | 81871574 | 81871884 | 261 | S | intergene | na | na | na |
| 9 | 83727722 | 83727976 | 262 | S | intergene | na | na | na |
| 9 | 86590284 | 86590490 | 263 | M | exon_intron, intron | HNRNPK-001,HNRNPK-002,HNRNPK-003,HNRNPK-005,HNRNPK-006,HNRNPK-008,HNRNPK-011,HNRNPK-012,HNRNPK-201,HNRNPK-202,HNRNPK-203,RP11-575L7 8,RP11-575L7 8-001 | na | yes |
| 9 | 86590492 | 86590758 | 264 | M | downstream, exon,intron, upstream | HNRNPK-001,HNRNPK-002,HNRNPK-003,HNRNPK-005,HNRNPK-006,HNRNPK-008,HNRNPK-011,HNRNPK-012,HNRNPK-202,HNRNPK-203,RP11-575L7 8-001 | na | yes |
| 9 | 108118471 | 108118687 | 265 | M | exon,  exon_intron | SLC44A1-001,SLC44A1-002,SLC44A1-003,SLC44A1-004,SLC44A1-201 | na | yes |
| 9 | 109378292 | 109378534 | 266 | M | exon, upstream | RP11-308N19 4-002 | na | na |
| 9 | 125053890 | 125054092 | 267 | M | exon_intron, intron | MRRF-001,MRRF-002,MRRF-004,MRRF-008,MRRF-009,MRRF-201,MRRF-202,MRRF-204 | na | yes |
| 9 | 125606828 | 125607078 | 268 | M | downstream, exon | RC3H2-001 | na | yes |
| 9 | 126537937 | 126538153 | 269 | S | intron | DENND1A-002,DENND1A-003,DENND1A-004,DENND1A-007 | na | na |
| 1 | 63369880 | 63370169 | 27 | M | downstream, upstream | na | na | na |
| 9 | 128304069 | 128304346 | 270 | S | intron | MAPKAP1-003,MAPKAP1-005,MAPKAP1-007,MAPKAP1-008,MAPKAP1-009,MAPKAP1-015,MAPKAP1-016,MAPKAP1-017 | na | na |
| 9 | 128304352 | 128304562 | 271 | S | intron | MAPKAP1-003,MAPKAP1-005,MAPKAP1-007,MAPKAP1-008,MAPKAP1-009,MAPKAP1-015,MAPKAP1-016,MAPKAP1-017 | na | na |
| 9 | 128432588 | 128432800 | 272 | S | intron | MAPKAP1-003,MAPKAP1-006,MAPKAP1-007,MAPKAP1-008,MAPKAP1-009,MAPKAP1-012,MAPKAP1-013,MAPKAP1-015 | na | na |
| 9 | 128517598 | 128517918 | 273 | S | intron | PBX3-001,PBX3-002,PBX3-003,PBX3-005,PBX3-006,PBX3-007,PBX3-009,PBX3-010,PBX3-012 | na | na |
| 9 | 128521871 | 128522197 | 274 | S | intron | PBX3-001,PBX3-002,PBX3-003,PBX3-005,PBX3-006,PBX3-007,PBX3-009,PBX3-010,PBX3-012 | na | na |
| 9 | 128584116 | 128584370 | 275 | S | intron | PBX3-001,PBX3-002,PBX3-005,PBX3-006,PBX3-007,PBX3-009,PBX3-010,PBX3-012 | na | na |
| 9 | 128605812 | 128606243 | 276 | M | exon_intron, intron | PBX3-001,PBX3-002,PBX3-005,PBX3-006,PBX3-007,PBX3-009,PBX3-010,PBX3-012,PBX3-013,PBX3-014 | na | yes |
| 9 | 128607710 | 128607985 | 277 | S | intron | PBX3-001,PBX3-002,PBX3-005,PBX3-006,PBX3-007,PBX3-009,PBX3-010,PBX3-012,PBX3-013,PBX3-014 | na | na |
| 9 | 128646165 | 128646401 | 278 | S | intron | PBX3-001,PBX3-002,PBX3-005,PBX3-006,PBX3-007,PBX3-008,PBX3-009,PBX3-010,PBX3-012,PBX3-013,PBX3-014 | na | na |
| 9 | 128672559 | 128672894 | 279 | S | intron | PBX3-001,PBX3-002,PBX3-005,PBX3-006,PBX3-007,PBX3-008,PBX3-009,PBX3-010,PBX3-012,PBX3-013,PBX3-014 | na | na |
| 1 | 70696713 | 70697067 | 28 | M | exon,  exon_intron,  intron | SRSF11-001,SRSF11-002,SRSF11-003,SRSF11-005,SRSF11-007,SRSF11-008,SRSF11-010,SRSF11-011,SRSF11-012,SRSF11-015,SRSF11-201,SRSF11-203 | na | yes |
| 9 | 128678006 | 128678225 | 280 | M | exon,  exon_intron,  intron | PBX3-001,PBX3-002,PBX3-005,PBX3-006,PBX3-007,PBX3-008,PBX3-009,PBX3-010,PBX3-012,PBX3-013,PBX3-014 | na | yes |
| 9 | 135495525 | 135495762 | 281 | S | intron | DDX31-001,DDX31-002 | na | na |
| 9 | 140042490 | 140042696 | 282 | M | exon_intron, intron | GRIN1-002,GRIN1-005,GRIN1-006,GRIN1-007,GRIN1-008,GRIN1-009,GRIN1-010,GRIN1-012,GRIN1-013 | na | na |
| 10 | 50604757 | 50605033 | 283 | S | upstream | na | na | na |
| 10 | 50606856 | 50607064 | 284 | S | upstream | na | na | na |
| 10 | 70515991 | 70516222 | 285 | M | downstream, exon,intron, upstream | CCAR1-001,CCAR1-005,CCAR1-006,CCAR1-009,CCAR1-010,CCAR1-011,CCAR1-012,CCAR1-013,CCAR1-014,CCAR1-015,CCAR1-201 | na | yes |
| 10 | 77139024 | 77139275 | 286 | M | downstream, intron | ZNF503-005 | na | na |
| 10 | 77495943 | 77496199 | 287 | S | intron | C10orf11-006 | na | na |
| 10 | 77727183 | 77727405 | 288 | S | intron | C10orf11-001,C10orf11-006 | na | na |
| 10 | 77990539 | 77990792 | 289 | S | intron | C10orf11-001,C10orf11-006,C10orf11-007 | na | na |
| 1 | 87822383 | 87822601 | 29 | S | intron | RP4-544H6 2,RP4-544H6 2-001 | na | na |
| 10 | 78042282 | 78042487 | 290 | M | downstream, exon,intron | C10orf11-001,C10orf11-006,C10orf11-007,RP11-369F10 3-001 | na | na |
| 10 | 78283624 | 78283854 | 291 | S | intron | C10orf11-001,C10orf11-002,C10orf11-003,C10orf11-004,C10orf11-005,C10orf11-006,C10orf11-007,C10orf11-008 | na | na |
| 10 | 98715455 | 98715671 | 292 | M | exon,intron | LCOR-009,LCOR-010,LCOR-012,LCOR-014,LCOR-015,LCOR-201 | na | na |
| 10 | 102372669 | 102372911 | 293 | S | intergene | na | na | na |
| 10 | 102373617 | 102374060 | 294 | S | intergene | na | na | na |
| 10 | 102375100 | 102375308 | 295 | S | intergene | na | na | na |
| 10 | 102415106 | 102415566 | 296 | S | intergene | na | yes | na |
| 10 | 102419220 | 102419583 | 297 | S | intergene | na | na | na |
| 10 | 102447658 | 102448016 | 298 | S | intergene | na | na | na |
| 10 | 102509435 | 102509644 | 299 | M | exon,  exon_intron,  intron | PAX2-001,PAX2-002,PAX2-003,PAX2-004,PAX2-005,PAX2-006,PAX2-007,PAX2-008,PAX2-201,PAX2-202 | na | yes |
| 1 | 10751165 | 10751389 | 3 | S | intron | CASZ1-001,CASZ1-003,CASZ1-006,CASZ1-007 | na | na |
| 1 | 88029000 | 88029242 | 30 | S | intergene | na | na | na |
| 10 | 102547118 | 102547325 | 300 | S | intron | PAX2-001,PAX2-002,PAX2-005,PAX2-006,PAX2-007,PAX2-008 | na | na |
| 10 | 102567791 | 102568074 | 301 | S | intron | PAX2-001,PAX2-002,PAX2-005,PAX2-006,PAX2-007 | na | na |
| 10 | 102979180 | 102979520 | 302 | S | intergene | na | na | na |
| 10 | 103052427 | 103052698 | 303 | S | intergene | na | yes | na |
| 10 | 103082504 | 103082775 | 304 | M | exon,  exon_intron, upstream | RP11-107I14 5,RP11-107I14 5-001 | na | na |
| 10 | 103211435 | 103211739 | 305 | S | intron | BTRC-001,BTRC-002,BTRC-004,BTRC-005,BTRC-006 | na | na |
| 10 | 103212039 | 103212262 | 306 | S | intron | BTRC-001,BTRC-002,BTRC-004,BTRC-005,BTRC-006 | na | na |
| 10 | 103243983 | 103244214 | 307 | S | intron | BTRC-001,BTRC-002,BTRC-004,BTRC-005,BTRC-006 | na | na |
| 10 | 103245812 | 103246088 | 308 | S | intron | BTRC-001,BTRC-002,BTRC-004,BTRC-005,BTRC-006 | na | na |
| 10 | 103267031 | 103267298 | 309 | S | intron | BTRC-001,BTRC-002,BTRC-004,BTRC-005,BTRC-006 | na | na |
| 1 | 88928018 | 88928270 | 31 | S | intergene | na | yes | na |
| 10 | 114404223 | 114404451 | 310 | S | intron | VTI1A-001,VTI1A-009 | na | na |
| 10 | 120074402 | 120074620 | 311 | S | intron | FAM204A,C10orf84 | na | na |
| 10 | 120076537 | 120076858 | 312 | S | intron | FAM204A,C10orf84 | na | na |
| 10 | 121340174 | 121340404 | 313 | M | exon,intron | TIAL1-001,TIAL1-002,TIAL1-005,TIAL1-006,TIAL1-007,TIAL1-008,TIAL1-009,TIAL1-012,TIAL1-201 | na | yes |
| 10 | 124852203 | 124852404 | 314 | S | intergene | na | na | na |
| 10 | 124852615 | 124852849 | 315 | S | intergene | na | na | na |
| 10 | 126905565 | 126905804 | 316 | S | intergene | na | na | na |
| 10 | 131446338 | 131446555 | 317 | S | intron | MGMT-001 | na | na |
| 10 | 131691586 | 131691906 | 318 | S | intergene | na | na | na |
| 11 | 8304695 | 8305010 | 319 | S | intergene | na | na | na |
| 11 | 8317834 | 8318168 | 320 | S | intergene | na | na | na |
| 11 | 15624424 | 15624627 | 321 | S | intron | RP11-531H8 2,RP11-531H8 2-001 | na | na |
| 11 | 16316351 | 16316573 | 322 | S | intron | SOX6-001,SOX6-002,SOX6-003,SOX6-009,SOX6-011,SOX6-012,SOX6-201 | na | na |
| 11 | 16475336 | 16475535 | 323 | S | intron | SOX6-006,SOX6-011,SOX6-201 | na | na |
| 11 | 30557521 | 30557745 | 324 | M | exon_intron, intron | MIR4454-001,MIR4454-002,MPPED2-001,MPPED2-003,MPPED2-005,MPPED2-006,MPPED2-009 | na | na |
| 11 | 31685644 | 31685878 | 325 | S | intron | ELP4-001,ELP4-003,ELP4-004,MIR4454-001,MIR4454-002,Z83001 1-002 | na | na |
| 11 | 31785680 | 31785994 | 326 | S | intron | ELP4-001,ELP4-003,ELP4-004,MIR4454-001,MIR4454-002,Z83001 1-001,Z83001 1-002,Z83001 1-003 | na | na |
| 11 | 31786284 | 31786551 | 327 | S | intron | ELP4-001,ELP4-003,ELP4-004,MIR4454-001,MIR4454-002,Z83001 1-001,Z83001 1-002,Z83001 1-003 | na | na |
| 11 | 31825663 | 31825893 | 328 | M | exon,intron | MIR4454-001,MIR4454-002,PAX6-001,PAX6-002,PAX6-003,PAX6-004,PAX6-005,PAX6-008,PAX6-009,PAX6-010,PAX6-011,PAX6-012,PAX6-013,PAX6-016,PAX6-018,PAX6-019,PAX6-021,PAX6-024,PAX6-029,PAX6-201 | na | yes |
| 11 | 32197992 | 32198298 | 329 | S | intron | MIR4454-001,MIR4454-002,RP1-65P5 1,RP1-65P5 1-001 | na | na |
| 1 | 97271737 | 97272048 | 33 | M | exon,  exon_intron,  intron | PTBP2-001,PTBP2-002,PTBP2-003,PTBP2-004,PTBP2-005,PTBP2-006,PTBP2-007,PTBP2-008,PTBP2-010,PTBP2-201 | na | na |
| 11 | 66393896 | 66394102 | 330 | M | exon_intron, intron | MIR4454-001,MIR4454-002,RBM14-001,RBM14-002,RBM14-010,RBM14-014,RBM14-RBM4,RBM14-RBM4-002,RBM14-RBM4-003,RBM14-RBM4-004,RBM4-002,RBM4-003 | na | yes |
| 11 | 83195159 | 83195376 | 331 | M | exon,  exon_intron, intron | DLG2-001,DLG2-003,DLG2-007,DLG2-008,DLG2-009,DLG2-010,DLG2-012,DLG2-013,DLG2-014,DLG2-015,DLG2-016,DLG2-023,DLG2-024,DLG2-201,DLG2-204,MIR4454-001,MIR4454-002 | na | na |
| 11 | 116232690 | 116233026 | 332 | S | intron | MIR4454-001,MIR4454-002 | na | na |
| 11 | 124644647 | 124644916 | 333 | M | exon,intron | MIR4454-001,MIR4454-002,MSANTD2-001,MSANTD2-002,MSANTD2-003,MSANTD2-004,MSANTD2-005 | na | yes |
| 11 | 131867945 | 131868166 | 334 | S | intron | MIR4454-001,MIR4454-002,NTM-001,NTM-002,NTM-003,NTM-007,NTM-008,NTM-013,NTM-016 | na | na |
| 12 | 16715414 | 16715627 | 335 | M | intron, upstream | LMO3-001,LMO3-002,LMO3-003,LMO3-004,LMO3-005,LMO3-006,LMO3-007,LMO3-009,LMO3-010,LMO3-011,LMO3-012,LMO3-013,LMO3-014,LMO3-016,LMO3-017,LMO3-019,LMO3-020,LMO3-021,LMO3-022,LMO3-023,LMO3-024,LMO3-025,LMO3-027,LMO3-028,LMO3-029,LMO3-031,LMO3-202,MGST1-015,MGST1-017 | na | na |
| 12 | 24292006 | 24292256 | 336 | S | intergene | na | na | na |
| 12 | 41749179 | 41749396 | 337 | S | intron | PDZRN4-001 | na | na |
| 12 | 53858489 | 53858711 | 338 | M | downstream, exon,  exon_intron,  intron | PCBP2-002,PCBP2-003,PCBP2-004,PCBP2-005,PCBP2-006,PCBP2-007,PCBP2-008,PCBP2-009,PCBP2-011,PCBP2-012,PCBP2-013,PCBP2-020,PCBP2-021,PCBP2-023,PCBP2-025,PCBP2-026,PCBP2-027,PCBP2-028,PCBP2-203,RP11-793H13 8,RP11-793H13 8-001 | na | yes |
| 12 | 54071096 | 54071347 | 339 | M | exon, upstream | ATP5G2-002,ATP5G2-004 | na | yes |
| 1 | 97280172 | 97280379 | 34 | M | downstream, exon | PTBP2-001,PTBP2-002,PTBP2-003,PTBP2-004 | na | yes |
| 12 | 54090832 | 54091090 | 340 | S | intergene | na | yes | na |
| 12 | 54382918 | 54383231 | 341 | M | exon_intron, intron, upstream | HOXC10-001,HOXC10-003,HOXC10-004,HOXC10-005,HOXC5-003 | na | yes |
| 12 | 54410494 | 54410720 | 342 | M | downstream, exon,intron, upstream | HOXC4-002,HOXC5-003,HOXC6-002,HOXC6-003,HOXC6-004 | na | yes |
| 12 | 54422441 | 54422828 | 343 | M | exon,  exon_intron,  intron,  upstream | HOXC4-002,HOXC5-002,HOXC5-003,HOXC6-001,HOXC6-002,HOXC6-003,HOXC6-004 | na | yes |
| 12 | 54426886 | 54427139 | 344 | M | downstream, exon,intron, upstream | HOXC4-002,HOXC5-001,HOXC5-002,HOXC5-003 | na | yes |
| 12 | 54447600 | 54447900 | 345 | M | exon,  exon_intron, upstream | HOXC4-001,HOXC4-002 | na | yes |
| 12 | 106976510 | 106976711 | 346 | M | exon,intron, upstream | RFX4-002,RP11-144F15 1,RP11-144F15 1-001,RP11-144F15 1-002 | na | yes |
| 13 | 71793988 | 71794196 | 347 | S | intergene | na | na | na |
| 13 | 72063357 | 72063596 | 348 | S | intron | DACH1-001,DACH1-002 | na | na |
| 13 | 72121302 | 72121504 | 349 | S | intron | DACH1-001,DACH1-002 | na | na |
| 1 | 98002282 | 98002486 | 35 | S | intron | DPYD-001 | na | na |
| 13 | 72256100 | 72256339 | 350 | S | intron | DACH1-001,DACH1-002 | na | na |
| 13 | 72668900 | 72669154 | 351 | S | intergene | na | na | na |
| 13 | 72694165 | 72694364 | 352 | S | intergene | na | na | na |
| 13 | 72771553 | 72771875 | 353 | S | intergene | na | na | na |
| 13 | 78976829 | 78977063 | 354 | S | intron | BX647243,AK095779,RNF219AS1 | na | na |
| 13 | 95618882 | 95619109 | 355 | S | intergene | na | na | na |
| 13 | 98008820 | 98009070 | 356 | M | exon,  exon_intron,  intron | MBNL2-001,MBNL2-002,MBNL2-003,MBNL2-004,MBNL2-201 | na | yes |
| 13 | 112716337 | 112716578 | 357 | S | intergene | na | na | na |
| 14 | 26378034 | 26378259 | 358 | S | intron | HMGN2P6-001,RP11-314P15 2,RP11-314P15 2-001 | na | na |
| 14 | 26914968 | 26915291 | 359 | M | exon,intron | HMGN2P6-001,NOVA1-002,NOVA1-003,NOVA1-004 | na | yes |
| 1 | 109240428 | 109240691 | 36 | M | exon,intron | PRPF38B-001,PRPF38B-002,PRPF38B-005 | na | na |
| 14 | 26915382 | 26915668 | 360 | M | exon,intron | HMGN2P6-001,NOVA1-002,NOVA1-003,NOVA1-004 | na | yes |
| 14 | 29233135 | 29233401 | 361 | M | intron, upstream | HMGN2P6-001,RP11-966I7 1,RP11-966I7 1-001,RP11-966I7 1-002,RP11-966I7 1-003 | na | na |
| 14 | 29348752 | 29348990 | 362 | S | intron | CTD-2384A14 1,CTD-2384A14 1-001,HMGN2P6-001 | na | na |
| 14 | 29861319 | 29861583 | 363 | S | intron | HMGN2P6-001,RP11-260G13 1,RP11-260G13 1-001 | na | na |
| 14 | 30712759 | 30712965 | 364 | S | intron | CTD-2251F13 1,CTD-2251F13 1-001,CTD-2251F13 1-002,HMGN2P6-001 | na | na |
| 14 | 30742362 | 30742639 | 365 | S | intron | CTD-2251F13 1,CTD-2251F13 1-001,CTD-2251F13 1-002,HMGN2P6-001 | na | na |
| 14 | 31382707 | 31382908 | 366 | M | exon_intron, intron | HMGN2P6-001,STRN3-001,STRN3-002,STRN3-003,STRN3-004,STRN3-005,STRN3-007 | na | na |
| 14 | 33844463 | 33844760 | 367 | S | intron | HMGN2P6-001,NPAS3-001,NPAS3-004,NPAS3-005,NPAS3-006,NPAS3-007,NPAS3-008,NPAS3-009,NPAS3-010,NPAS3-011,NPAS3-013 | na | na |
| 14 | 34068576 | 34068803 | 368 | S | intron | HMGN2P6-001,NPAS3-001,NPAS3-004,NPAS3-005,NPAS3-006,NPAS3-007,NPAS3-008,NPAS3-009,NPAS3-010,NPAS3-013 | na | na |
| 14 | 34122617 | 34122829 | 369 | S | intron | HMGN2P6-001,NPAS3-001,NPAS3-004,NPAS3-005,NPAS3-006,NPAS3-007,NPAS3-008,NPAS3-009,NPAS3-010 | na | na |
| 1 | 115280053 | 115280254 | 37 | M | exon_intron, intron | CSDE1-001,CSDE1-002,CSDE1-003,CSDE1-004,CSDE1-007,CSDE1-008,CSDE1-009,CSDE1-014,CSDE1-015 | na | na |
| 14 | 34202571 | 34202963 | 370 | S | intron | HMGN2P6-001,NPAS3-001,NPAS3-004,NPAS3-005,NPAS3-006,NPAS3-007,NPAS3-010 | na | na |
| 14 | 36020189 | 36020484 | 371 | S | intron | HMGN2P6-001,RALGAPA1-001,RALGAPA1-002,RALGAPA1-003,RALGAPA1-006,RALGAPA1-007,RALGAPA1-015,RALGAPA1-019 | na | na |
| 14 | 36043035 | 36043311 | 372 | S | intron | HMGN2P6-001,RALGAPA1-001,RALGAPA1-002,RALGAPA1-003,RALGAPA1-006,RALGAPA1-007,RALGAPA1-019 | na | na |
| 14 | 36581807 | 36582200 | 373 | S | intron | HMGN2P6-001,RP11-259K15 3,RP11-259K15 3-001 | na | na |
| 14 | 37715913 | 37716136 | 374 | S | intron | HMGN2P6-001,MIPOL1-001,MIPOL1-002,MIPOL1-003,MIPOL1-004,MIPOL1-007,MIPOL1-008,MIPOL1-011,MIPOL1-012 | na | na |
| 14 | 37777220 | 37777519 | 375 | M | exon,intron | HMGN2P6-001,MIPOL1-001,MIPOL1-002,MIPOL1-003,MIPOL1-004,MIPOL1-007,MIPOL1-010,MIPOL1-201,MIPOL1-202,MIPOL1-203,MIPOL1-204 | na | na |
| 14 | 45565750 | 45566039 | 376 | M | exon,intron | HMGN2P6-001,PRPF39-001,PRPF39-002,PRPF39-003,PRPF39-004,PRPF39-006,PRPF39-007,PRPF39-013 | na | yes |
| 14 | 45579023 | 45579239 | 377 | M | exon,intron, upstream | HMGN2P6-001,PRPF39-001,PRPF39-002,PRPF39-003,PRPF39-004,PRPF39-006,PRPF39-007,PRPF39-008,PRPF39-011,PRPF39-012 | na | yes |
| 14 | 80327477 | 80327727 | 378 | M | exon,intron | NRXN3-001,NRXN3-002,NRXN3-004,NRXN3-005,NRXN3-006,NRXN3-007,NRXN3-013,NRXN3-203 | na | yes |
| 14 | 97431368 | 97431619 | 379 | S | intergene | na | na | na |
| 1 | 163939955 | 163940178 | 38 | S | intergene | na | na | na |
| 14 | 97762594 | 97762825 | 380 | S | intergene | na | na | na |
| 14 | 97879290 | 97879527 | 381 | S | intergene | na | na | na |
| 15 | 35918912 | 35919111 | 382 | S | intron | RP11-702M1 1,RP11-702M1 1-001,RP11-702M1 1-003 | na | na |
| 15 | 36819927 | 36820195 | 383 | S | intergene | na | na | na |
| 15 | 36965393 | 36965658 | 384 | M | intron, upstream | C15orf41-001,C15orf41-002,C15orf41-003,C15orf41-004,C15orf41-007,C15orf41-008,C15orf41-010,C15orf41-011,C15orf41-201 | na | na |
| 15 | 37185670 | 37185878 | 385 | S | intron | MEIS2-001,MEIS2-002,MEIS2-003,MEIS2-005,MEIS2-007,MEIS2-008,MEIS2-009,MEIS2-012,MEIS2-016,MEIS2-020,MEIS2-024,MEIS2-025,MEIS2-027 | na | na |
| 15 | 37522009 | 37522211 | 386 | S | intergene | na | na | na |
| 15 | 42032054 | 42032291 | 387 | M | exon,  exon_intron | MGA-001,MGA-003,MGA-005,MGA-201,MGA-202,MGA-203 | na | na |
| 15 | 57425525 | 57425822 | 388 | S | intron | TCF12-001,TCF12-002,TCF12-003,TCF12-013,TCF12-016,TCF12-024,TCF12-025,TCF12-026,TCF12-027,TCF12-028 | na | na |
| 15 | 67665385 | 67665655 | 389 | S | intron | IQCH-001,IQCH-002,IQCH-005,IQCH-006 | na | na |
| 1 | 164023535 | 164023890 | 39 | S | intergene | na | na | na |
| 15 | 67878170 | 67878374 | 390 | M | exon,  exon_intron | MAP2K5-001,MAP2K5-003,MAP2K5-004,MAP2K5-005,MAP2K5-006,MAP2K5-007,MAP2K5-013 | na | na |
| 15 | 68040304 | 68040614 | 391 | M | exon, exon_intron, intron | MAP2K5-001,MAP2K5-002,MAP2K5-005,MAP2K5-007,MAP2K5-009,MAP2K5-010,MAP2K5-013 | na | na |
| 15 | 70392134 | 70392389 | 392 | S | upstream | na | yes | na |
| 15 | 74914242 | 74914516 | 393 | M | exon,  exon_intron,  intron | CLK3-001,CLK3-002,CLK3-003,CLK3-006,CLK3-007,CLK3-008,CLK3-010,CLK3-013,CLK3-014,CLK3-015,CLK3-016,CLK3-018,CLK3-026,CLK3-027,CLK3-201,CLK3-202 | na | na |
| 15 | 97236100 | 97236301 | 394 | S | intergene | na | na | na |
| 16 | 24579003 | 24579251 | 395 | M | exon_intron,  intron | RBBP6-001,RBBP6-002,RBBP6-004,RBBP6-005,RBBP6-006 | na | na |
| 16 | 49094731 | 49094938 | 396 | S | intergene | na | na | na |
| 16 | 49735848 | 49736158 | 397 | S | intron | ZNF423-001,ZNF423-002,ZNF423-003,ZNF423-005 | yes | na |
| 16 | 49890891 | 49891212 | 398 | S | intron | ZNF423-002 | yes | na |
| 16 | 50848991 | 50849204 | 399 | S | intergene | na | na | na |
| 1 | 10758249 | 10758607 | 4 | S | intron | CASZ1-001,CASZ1-003 | na | na |
| 1 | 164637962 | 164638208 | 40 | S | intron | PBX1-001,PBX1-002,PBX1-003,PBX1-004,PBX1-007,PBX1-009,PBX1-010,PBX1-011,PBX1-012,PBX1-013,PBX1-015,PBX1-016,PBX1-017 | na | na |
| 16 | 51671487 | 51671692 | 400 | S | intergene | na | na | na |
| 16 | 52494715 | 52494964 | 401 | S | intron | TOX3-002,TOX3-003,TOX3-004 | na | na |
| 16 | 53654385 | 53654629 | 402 | S | intron | RPGRIP1L-001,RPGRIP1L-003,RPGRIP1L-007,RPGRIP1L-009 | na | na |
| 16 | 54323855 | 54324060 | 403 | S | upstream | na | na | na |
| 16 | 55223357 | 55223591 | 404 | S | intergene | na | na | na |
| 16 | 59575395 | 59575604 | 405 | S | downstream | na | na | na |
| 16 | 69680364 | 69680574 | 406 | M | exon,  exon_intron, intron | NFAT5-001,NFAT5-002,NFAT5-003,NFAT5-004,NFAT5-006,NFAT5-009,NFAT5-201 | na | yes |
| 16 | 69681155 | 69681480 | 407 | M | exon,intron | NFAT5-001,NFAT5-002,NFAT5-003,NFAT5-004,NFAT5-006,NFAT5-007,NFAT5-009,NFAT5-201,NFAT5-202 | na | yes |
| 16 | 72821045 | 72821296 | 408 | M | downstream,  exon, exon_intron, intron, upstream | RP5-991G20 1,RP5-991G20 1-001,ZFHX3-001,ZFHX3-002 | na | yes |
| 16 | 73092871 | 73093114 | 409 | M | exon, exon_intron, intron | RP11-346C20 1,RP11-346C20 1-001,ZFHX3-002 | yes | yes |
| 1 | 213598767 | 213598982 | 41 | S | downstream | na | na | na |
| 17 | 35012230 | 35012448 | 410 | S | intron | CYCS-001,YBX2-001 | na | na |
| 17 | 35329619 | 35329847 | 411 | S | intron | CYCS-001,YBX2-001 | na | na |
| 17 | 35336486 | 35336753 | 412 | S | intron | CYCS-001,YBX2-001 | yes | na |
| 17 | 37566519 | 37566790 | 413 | M | exon,intron | CYCS-001,MED1-001,MED1-002,MED1-003,YBX2-001 | na | yes |
| 17 | 38249174 | 38249419 | 414 | M | exon,  exon_intron,  intron | CYCS-001,NR1D1-001,THRA-001,THRA-003,THRA-005,THRA-201,YBX2-001 | na | yes |
| 17 | 46663906 | 46664112 | 415 | M | downstream,  intron,  upstream | CYCS-001,HOXB-AS3,HOXB-AS3-012,HOXB3-002,HOXB3-003,HOXB3-004,HOXB3-006,HOXB3-013,YBX2-001 | na | na |
| 17 | 46670887 | 46671172 | 416 | M | downstream, exon,intron | CYCS-001,HOXB-AS3,HOXB-AS3-005,HOXB-AS3-007,HOXB-AS3-009,HOXB-AS3-010,HOXB-AS3-012,HOXB-AS3-014,HOXB-AS3-015,HOXB-AS3-017,HOXB3-013,HOXB5-001,YBX2-001 | na | yes |
| 17 | 46682312 | 46682533 | 417 | M | downstream, exon,intron,  upstream | CYCS-001,HOXB-AS3,HOXB-AS3-001,HOXB-AS3-003,HOXB-AS3-004,HOXB-AS3-005,HOXB-AS3-017,HOXB6-001,HOXB6-002,YBX2-001 | na | yes |
| 17 | 56082228 | 56082444 | 418 | M | exon,intron | CYCS-001,RP11-159D12 5-001,SRSF1-001,SRSF1-002,SRSF1-003,SRSF1-004,SRSF1-005,SRSF1-006,SRSF1-008,SRSF1-009,YBX2-001 | na | yes |
| 17 | 56082715 | 56083003 | 419 | M | exon,intron | CYCS-001,SRSF1-001,SRSF1-002,SRSF1-003,SRSF1-004,SRSF1-005,SRSF1-008,YBX2-001 | na | yes |
| 1 | 215889032 | 215889297 | 42 | S | intron | USH2A-002 | na | na |
| 17 | 62496771 | 62497003 | 420 | M | exon_intron, upstream | CYCS-001,DDX5-001,DDX5-002,DDX5-003,DDX5-004,DDX5-005,DDX5-006,DDX5-023,DDX5-030,DDX5-031,DDX5-032,MIR3064-202,YBX2-001 | na | yes |
| 18 | 22693155 | 22693499 | 421 | S | intron | ZNF521 | na | na |
| 18 | 22748188 | 22748413 | 422 | S | intron | ZNF521 | na | na |
| 18 | 22756355 | 22756577 | 423 | S | intron | ZNF521 | na | na |
| 18 | 22767779 | 22767993 | 424 | S | intron | ZNF521 | na | na |
| 18 | 22865207 | 22865531 | 425 | S | intron | ZNF521 | na | na |
| 18 | 23915592 | 23915853 | 426 | S | intron | TAF04B | na | na |
| 18 | 24237199 | 24237413 | 427 | S | exon | KCTD1-008,KCTD1-009 | yes | yes |
| 18 | 30353209 | 30353447 | 428 | M | exon_intron, upstream | AC012123 1-001 | na | yes |
| 18 | 34480541 | 34480778 | 429 | S | intergene | na | na | na |
| 1 | 243896035 | 243896291 | 43 | S | intron | AKT3-001,AKT3-002,AKT3-003,AKT3-004,AKT3-007,AKT3-008,RP11-370K11 1,RP11-370K11 1-001 | na | na |
| 18 | 35063650 | 35063862 | 430 | S | intron | CELF4 | na | na |
| 18 | 35178600 | 35178829 | 431 | S | intergene | na | na | na |
| 18 | 35564932 | 35565142 | 432 | S | intergene | na | na | na |
| 18 | 36063632 | 36063837 | 433 | S | intergene | na | na | na |
| 18 | 44770788 | 44771036 | 434 | S | intergene | na | na | na |
| 18 | 53089931 | 53090157 | 435 | S | intron | TCF4-001,TCF4-002,TCF4-003,TCF4-004,TCF4-005,TCF4-006,TCF4-007,TCF4-008,TCF4-009,TCF4-017,TCF4-019,TCF4-021,TCF4-022,TCF4-023,TCF4-025,TCF4-026,TCF4-027,TCF4-028,TCF4-029,TCF4-030,TCF4-040,TCF4-041,TCF4-045,TCF4-046,TCF4-201 | na | na |
| 18 | 53254241 | 53254450 | 436 | M | exon_intron,  intron | TCF4-001,TCF4-002,TCF4-003,TCF4-004,TCF4-005,TCF4-006,TCF4-019,TCF4-020,TCF4-021,TCF4-022,TCF4-023,TCF4-024,TCF4-039,TCF4-040,TCF4-041,TCF4-045,TCF4-046,TCF4-201 | na | yes |
| 18 | 72357658 | 72357872 | 437 | S | intron | ZNF407 | na | na |
| 18 | 72357874 | 72358114 | 438 | S | intron | ZNF407 | na | na |
| 18 | 72363031 | 72363293 | 439 | S | intron | ZNF407 | na | na |
| 1 | 244217707 | 244217936 | 44 | S | exon | ZBTB18-001 | na | na |
| 18 | 72363365 | 72363693 | 440 | S | intron | ZNF407 | na | na |
| 18 | 72568592 | 72568839 | 441 | S | intron | ZNF407 | na | na |
| 18 | 72592712 | 72592960 | 442 | S | intron | ZNF407 | na | na |
| 19 | 8527269 | 8527507 | 443 | S | exon | HNRNPM-001,HNRNPM-002,HNRNPM-003,HNRNPM-009,HNRNPM-014,HNRNPM-017 | na | na |
| 19 | 30494779 | 30495166 | 444 | S | intron | URI1-001,URI1-002,URI1-003,URI1-006,URI1-007,URI1-011,URI1-012 | na | na |
| 19 | 30566435 | 30566744 | 445 | S | intergene | na | na | na |
| 19 | 30747854 | 30748125 | 446 | S | intergene | na | na | na |
| 19 | 30767781 | 30768053 | 447 | S | intergene | na | na | na |
| 19 | 30841530 | 30841761 | 448 | S | intergene | na | na | na |
| 19 | 31003541 | 31003830 | 449 | S | intron | ZNF536 | na | na |
| 1 | 245016686 | 245016888 | 45 | M | exon,intron | HNRNPU-001,HNRNPU-002,HNRNPU-AS1,HNRNPU-AS1-002,HNRNPU-AS1-003 | na | yes |
| 19 | 31587626 | 31587836 | 450 | S | intergene | na | na | na |
| 19 | 31806620 | 31806844 | 451 | S | intron | TSHZ3-001,TSHZ3-002 | na | na |
| 19 | 31827947 | 31828150 | 452 | M | exon,intron | CTC-439O9 2-001,TSHZ3-001,TSHZ3-002 | na | na |
| 19 | 42437317 | 42437641 | 453 | S | intergene | na | na | na |
| 20 | 4866440 | 4866647 | 454 | M | downstream, exon,intron | SLC23A2-001,SLC23A2-002,SLC23A2-003,SLC23A2-004,SLC23A2-005 | na | na |
| 20 | 34328379 | 34328623 | 455 | M | exon,  exon_intron,  intron | RBM39-001,RBM39-002,RBM39-003,RBM39-004,RBM39-005,RBM39-006,RBM39-007,RBM39-008,RBM39-012,RBM39-013,RBM39-014,RBM39-015,RBM39-016,RBM39-017,RBM39-018,RBM39-019,RBM39-020,RBM39-021,RBM39-024,RBM39-039,RBM39-042,RBM39-043,RBM39-044,RBM39-045 | na | yes |
| 20 | 42087756 | 42088075 | 456 | M | exon_intron,  intron | SRSF6-001,SRSF6-002 | na | yes |
| 22 | 19395909 | 19396119 | 457 | M | exon,  exon_intron | C22orf39-003,HIRA-001,HIRA-002,HIRA-003,HIRA-004,HIRA-201,HIRA-202 | na | yes |
| 22 | 36148492 | 36148695 | 458 | M | exon_intron, intron | HMGB1P10-001,RBFOX2-001,RBFOX2-002,RBFOX2-003,RBFOX2-004,RBFOX2-005,RBFOX2-006,RBFOX2-008,RBFOX2-009,RBFOX2-010 | na | na |
| X | 21534560 | 21534814 | 459 | M | exon,  exon_intron,  intron | CNKSR2-003,CNKSR2-004,CNKSR2-005,CNKSR2-201 | na | na |
| 1 | 245017603 | 245017819 | 46 | M | exon,  exon_intron,  intron | HNRNPU-001,HNRNPU-002,HNRNPU-004,HNRNPU-AS1,HNRNPU-AS1-002,HNRNPU-AS1-003 | na | yes |
| X | 24823511 | 24823785 | 460 | S | intron | POLA1-001,POLA1-002,POLA1-005 | na | na |
| X | 24864797 | 24865193 | 461 | S | intron | POLA1-001,POLA1-005 | na | na |
| X | 24894826 | 24895604 | 462 | S | intron | POLA1-001,POLA1-005 | na | na |
| X | 24915882 | 24916156 | 463 | S | intron | POLA1-001,POLA1-005 | na | na |
| X | 24916158 | 24916927 | 464 | S | intron | POLA1-001,POLA1-005 | na | na |
| X | 24917481 | 24917790 | 465 | S | intron | POLA1-001,POLA1-005 | na | na |
| X | 24946458 | 24946806 | 466 | S | intron | POLA1-001,POLA1-005 | na | na |
| X | 25008354 | 25009084 | 467 | S | intron | POLA1-001,POLA1-005 | na | na |
| X | 25017563 | 25018051 | 468 | S | downstream | na | na | na |
| X | 25018053 | 25018274 | 469 | S | downstream | na | na | na |
| 2 | 7774651 | 7774877 | 47 | S | intergene | na | na | na |
| X | 25401216 | 25401556 | 470 | S | intergene | na | na | na |
| X | 41208371 | 41208609 | 471 | M | exon,intron | DDX3X-001,DDX3X-002 | na | yes |
| X | 41379306 | 41379507 | 472 | S | exon | CASK-001,CASK-002,CASK-004,CASK-005,CASK-007,CASK-012,CASK-201,CASK-202,CASK-AS1-001 | na | yes |
| X | 70373224 | 70373445 | 473 | M | exon,  exon_intron,  intron | NLGN3-001,NLGN3-002,NLGN3-003,NLGN3-004 | na | na |
| X | 70468843 | 70469052 | 474 | M | exon,  exon_intron,  intron | ZMYM3-001,ZMYM3-002,ZMYM3-005,ZMYM3-006,ZMYM3-007,ZMYM3-008,ZMYM3-009,ZMYM3-201 | na | yes |
| X | 70766055 | 70766451 | 475 | M | exon,  exon_intron,  intron | OGT-001,OGT-002,OGT-003,OGT-004,OGT-010 | na | na |
| X | 81789599 | 81789836 | 476 | S | intergene | na | na | na |
| X | 103041491 | 103041699 | 477 | M | exon,  exon_intron | PLP1-001,PLP1-002,PLP1-004,PLP1-005,PLP1-006,PLP1-007,PLP1-008,PLP1-009,PLP1-010,PLP1-011,PLP1-013,PLP1-015,PLP1-016,PLP1-017,PLP1-018,PLP1-019,PLP1-020,PLP1-201 | na | na |
| X | 122599457 | 122599708 | 478 | M | exon,  exon_intron,  intron | GRIA3-001,GRIA3-002,GRIA3-201,GRIA3-203 | na | na |
| X | 122613751 | 122614052 | 479 | M | exon,  exon_intron,  intron | GRIA3-001,GRIA3-002,GRIA3-203 | na | yes |
| 2 | 20478333 | 20478630 | 48 | M | exon,  exon_intron,  intron | PUM2-001,PUM2-002,PUM2-003,PUM2-202,PUM2-203 | na | na |
| X | 123235272 | 123235473 | 480 | M | exon,intron | STAG2-001,STAG2-006,STAG2-010,STAG2-018 | na | yes |
| X | 123235475 | 123235678 | 481 | M | exon,intron | STAG2-001,STAG2-010,STAG2-018 | na | yes |
| X | 139170488 | 139170782 | 482 | M | downstream, upstream | na | na | na |
| 3 | 17592729 | 17593130 | 483 | S | intron | TBC1D5-001,TBC1D5-002,TBC1D5-003,TBC1D5-004,TBC1D5-008,TBC1D5-009,TBC1D5-010,TBC1D5-011,TBC1D5-012,TBC1D5-013,TBC1D5-014,TBC1D5-015,TBC1D5-016,TBC1D5-017,TBC1D5-018,TBC1D5-019,TBC1D5-020,TBC1D5-022,TBC1D5-023,TBC1D5-024,TBC1D5-025,TBC1D5-029,TBC1D5-030 | na | na |
| 2 | 33813409 | 33813615 | 49 | M | exon,  exon_intron,  intron | FAM98A-001,FAM98A-002,FAM98A-004,FAM98A-007 | na | na |
| 1 | 10781351 | 10781564 | 5 | S | intron | CASZ1-001,CASZ1-003 | na | na |
| 2 | 38976301 | 38976522 | 50 | M | exon,  exon_intron,  intron,  upstream | SRSF7-001,SRSF7-002,SRSF7-003,SRSF7-004,SRSF7-007,SRSF7-010,SRSF7-011,SRSF7-012 | na | yes |
| 2 | 57972559 | 57972765 | 51 | S | intergene | na | na | na |
| 2 | 59108186 | 59108459 | 52 | S | intron | AC007092 1-001,AC007092 1-002,AC007092 1-003,AC007092 1-004,AC007092 1-005,AC007092 1-008 | na | na |
| 2 | 59133311 | 59133542 | 53 | S | intron | AC007092 1-001,AC007092 1-002,AC007092 1-003,AC007092 1-004,AC007092 1-005,AC007092 1-008 | na | na |
| 2 | 59199403 | 59199611 | 54 | S | intron | AC007092 1-001,AC007092 1-005,AC007092 1-008 | na | na |
| 2 | 59746578 | 59746817 | 55 | S | intron | AC007131 2-007 | na | na |
| 2 | 59947831 | 59948032 | 56 | S | intron | AC007131 2-006,AC007131 2-007 | na | na |
| 2 | 60139240 | 60139485 | 57 | S | intergene | na | na | na |
| 2 | 60297963 | 60298165 | 58 | S | intergene | na | na | na |
| 2 | 60298383 | 60298602 | 59 | S | intergene | na | na | na |
| 1 | 10795121 | 10795421 | 6 | S | intron | CASZ1-001,CASZ1-003 | na | na |
| 2 | 60441560 | 60441776 | 60 | S | intergene | na | na | na |
| 2 | 60687573 | 60687898 | 61 | M | exon,  exon_intron,  intron | BCL11A-001,BCL11A-002,BCL11A-003,BCL11A-006,BCL11A-007,BCL11A-008,BCL11A-010,BCL11A-013,BCL11A-201,BCL11A-202 | na | na |
| 2 | 60780682 | 60780915 | 62 | M | exon,  upstream | BCL11A-003 | na | yes |
| 2 | 61752501 | 61752778 | 63 | M | exon,  exon_intron,  intron | XPO1-001,XPO1-004,XPO1-005,XPO1-006,XPO1-008,XPO1-015,XPO1-017,XPO1-018,XPO1-019,XPO1-022,XPO1-023,XPO1-024 | na | yes |
| 2 | 63194091 | 63194335 | 64 | S | intron | EHBP1-001,EHBP1-002,EHBP1-003,EHBP1-007,EHBP1-009,EHBP1-015,EHBP1-016,EHBP1-017 | na | na |
| 2 | 66298591 | 66298802 | 65 | S | intron | FJ16124 | na | na |
| 2 | 73175003 | 73175249 | 66 | S | intron | SFXN5-001,SFXN5-002,SFXN5-003,SFXN5-004,SFXN5-005,SFXN5-008,SFXN5-009,SFXN5-010 | na | na |
| 2 | 104736726 | 104736942 | 67 | S | downstream | na | na | na |
| 2 | 143914722 | 143914976 | 68 | M | downstream,  intron | ARHGAP15-001,ARHGAP15-002,ARHGAP15-008 | na | na |
| 2 | 144112110 | 144112410 | 69 | S | intron | AC096558 1-001,AC096558 1-002,AC096558 1-003,ARHGAP15-001,ARHGAP15-003,ARHGAP15-004,ARHGAP15-005,ARHGAP15-008,RP11-570L15 2,RP11-570L15 2-001 | na | na |
| 1 | 10836133 | 10836388 | 7 | S | intron | CASZ1-001,CASZ1-003 | na | na |
| 2 | 144437339 | 144437575 | 70 | S | intron | ARHGAP15-001,ARHGAP15-013,RP11-434H14 1,RP11-434H14 1-001,RP11-434H14 1-002 | na | na |
| 2 | 144712856 | 144713103 | 71 | S | intron | AC016910 1-001,GTDC1-001,GTDC1-002,GTDC1-004,GTDC1-005,GTDC1-006,GTDC1-007,GTDC1-008,GTDC1-201,GTDC1-202,GTDC1-203 | na | na |
| 2 | 144714972 | 144715378 | 72 | S | intron | AC016910 1-001,GTDC1-001,GTDC1-002,GTDC1-004,GTDC1-005,GTDC1-006,GTDC1-007,GTDC1-008,GTDC1-201,GTDC1-202,GTDC1-203 | na | na |
| 2 | 144762313 | 144762513 | 73 | S | intron | GTDC1-001,GTDC1-002,GTDC1-004,GTDC1-005,GTDC1-006,GTDC1-007,GTDC1-008,GTDC1-201,GTDC1-202,GTDC1-203 | na | na |
| 2 | 144825963 | 144826500 | 74 | S | intron | GTDC1-001,GTDC1-002,GTDC1-003,GTDC1-004,GTDC1-005,GTDC1-006,GTDC1-007,GTDC1-008,GTDC1-014,GTDC1-201,GTDC1-202,GTDC1-203 | na | na |
| 2 | 145145850 | 145146085 | 75 | S | exon | ZEB2-001,ZEB2-011,ZEB2-013 | na | yes |
| 2 | 145161133 | 145161467 | 76 | S | intron | ZEB2-001,ZEB2-007,ZEB2-010,ZEB2-011,ZEB2-013,ZEB2-025,ZEB2-030 | na | na |
| 2 | 145185765 | 145186060 | 77 | M | exon,intron | ZEB2-001,ZEB2-004,ZEB2-006,ZEB2-007,ZEB2-010,ZEB2-011,ZEB2-013,ZEB2-019,ZEB2-025,ZEB2-026,ZEB2-030,ZEB2-032 | na | na |
| 2 | 145188354 | 145188601 | 78 | S | intron | ZEB2-001,ZEB2-004,ZEB2-006,ZEB2-007,ZEB2-010,ZEB2-011,ZEB2-014,ZEB2-015,ZEB2-017,ZEB2-018,ZEB2-019,ZEB2-021,ZEB2-023,ZEB2-024,ZEB2-025,ZEB2-030,ZEB2-031,ZEB2-032 | na | na |
| 2 | 145197177 | 145197471 | 79 | S | intron | ZEB2-001,ZEB2-004,ZEB2-006,ZEB2-007,ZEB2-010,ZEB2-011,ZEB2-014,ZEB2-015,ZEB2-017,ZEB2-018,ZEB2-019,ZEB2-021,ZEB2-023,ZEB2-024,ZEB2-025,ZEB2-030,ZEB2-031,ZEB2-032 | na | na |
| 1 | 10851818 | 10852033 | 8 | S | intron | CASZ1-001,CASZ1-003 | na | na |
| 2 | 145200852 | 145201145 | 80 | S | intron | ZEB2-001,ZEB2-004,ZEB2-006,ZEB2-007,ZEB2-010,ZEB2-011,ZEB2-014,ZEB2-015,ZEB2-017,ZEB2-018,ZEB2-019,ZEB2-021,ZEB2-023,ZEB2-024,ZEB2-025,ZEB2-030,ZEB2-031,ZEB2-032 | na | na |
| 2 | 147134065 | 147134275 | 81 | S | intergene | na | na | na |
| 2 | 156727099 | 156727308 | 82 | S | intergene | na | na | na |
| 2 | 156991627 | 156991922 | 83 | S | intron | BC032407 | na | na |
| 2 | 157194706 | 157194914 | 84 | S | intron | NR4A2-010 | na | na |
| 2 | 157551414 | 157551661 | 85 | S | intergene | na | na | na |
| 2 | 157660110 | 157660449 | 86 | S | intergene | na | na | na |
| 2 | 157900269 | 157900558 | 87 | S | intergene | na | na | na |
| 2 | 162095042 | 162095353 | 88 | M | downstream,  intron | AC009299 2-001,AC009299 2-002 | na | na |
| 2 | 162238673 | 162238979 | 89 | M | exon,intron | PSMD14-001,PSMD14-005 | na | na |
| 1 | 10925410 | 10925611 | 9 | S | intergene | na | na | na |
| 2 | 162273027 | 162273232 | 90 | M | downstream, exon | TBR1-001,TBR1-005 | na | na |
| 2 | 163045022 | 163045228 | 91 | S | intron | FAP-001,FAP-002,FAP-004,FAP-009,FAP-015 | na | na |
| 2 | 164450679 | 164450987 | 92 | S | intron | FIGN-004 | na | na |
| 2 | 164661907 | 164662169 | 93 | S | intron | AC092684 1-001 | na | na |
| 2 | 164844170 | 164844369 | 94 | S | intron | AC092684 1-001 | na | na |
| 2 | 171571530 | 171571780 | 95 | S | upstream | na | na | na |
| 2 | 172820674 | 172820934 | 96 | S | intron | HAT1-001,HAT1-002,HAT1-003,HAT1-004,SLC25A12-008,SLC25A12-009,SLC25A12-010 | na | na |
| 2 | 172822631 | 172823072 | 97 | M | exon,  exon_intron,  intron | HAT1-001,HAT1-002,HAT1-003,HAT1-004,HAT1-006,HAT1-201,SLC25A12-008,SLC25A12-009,SLC25A12-010 | na | yes |
| 2 | 172956518 | 172956755 | 98 | S | downstream | na | na | na |
| 2 | 172958381 | 172958778 | 99 | S | downstream | na | na | na |
| Abbreviations: chr, chromosome; uc, ultraconserved RNA; S, single uc; M, multiple uc; na, not annotated. | | | | | | | | |

**Supplementary Table S5. Primers used in the study.**

| **Procedures** | **Primer name** | **Sequence** |
| --- | --- | --- |
| RT-PCR | *CASZ1* | Fw - CAAAACAGACTCCATCACCACG |
|  |  | Rv - GTGCTGGCTGCCCGAGAAC |
| RT-PCR | uc.2+ | Fw - GTTGCCATGGAGACCTCATC |
|  |  | Rv - GACGCAAATTAGATGGTTATTCG |
| RT-PCR | uc.3+ | Fw - ATAACCCAACCCCCTCTCAT |
|  |  | Rv - AAAATGCCGATGTCGTCCTA |
| RT-PCR | uc.4+ | Fw - CTGTTAGCCAGCAATCTCCA |
|  |  | Rv - CGGATTTCTCCGTCATGAAT |
| RT-PCR | uc.5+ | Fw - ATGCAAATGCAGGAGAGGAT |
|  |  | Rv - TGTGACAATCAGTCACAGCA |
| RT-PCR | uc.6+ | Fw - TGAGTTGCCCTTAGCAACCT |
|  |  | Rv - CCGTCGCCATGACAACTAAT |
| RT-PCR | uc.7+ | Fw - CGTCTTGTATCCAGGGGAAG |
|  |  | Rv - TGGAGATTAAGGGGATGAGA |
| RT-PCR | uc.8+ | Fw - GGTCGCCATGGATATGACA |
|  |  | Rv - CACTGTGGCTTTAAACTCAGGA |
| 5′ RACE | GSP2 | Rv - TCCTGAGTTTAAAGCCACAGTG |
|  | nGSP2 | Rv - GGGGAAAGATACAAGGAGAA |
|  | ISP2 | Fv - ATCAAGAGGTTACGGGCTCA |
| 3′ RACE | GSP1 | Fw - AGAGAGAGATGACTTTCCTTG |
|  | nGSP1 | Fw - GGTCGCCATGGATATGACA |
|  | ISP1 | Rv - CTTTTCCCCTCTTCCCAGAG |
| RT-PCR | uc.195+ | Fw - GGGGTTACTCCCAGACTGAA |
|  |  | Rv - TGTCCTGAGGGTCAGGCTTA |
| RT-PCR | uc.217+A | Fw - CTGTCCGCTCCGTAGATTTT |
|  |  | Rv - CAGGTGCGAGGATAGCTACA |
| RT-PCR | uc.339+ | Fw - GATGAGGCCCCGAGTTTAAT |
|  |  | Rv - GGGCCCATATAAATCCCTCT |
| RT-PCR | *MMP9* | Fw - CGCAGACATCGTCATCCAGT |
|  |  | Rv - GGATTGGCCTTGGAAGATGA |
|  | siRNA- 1  anti uc.8+ | Guide - GGUUAAACAGAUCAUUCAAGGAAdTdT |
|  |  | Passenger - UUCCUUGAAUGAUCUGUUUAACCdTdT |
| uc.8+ silencing | siRNA- 2  anti uc.8+ | Guide - UUAAUGACUUUCUUUUUUUUCCUdTdT |
|  |  | Passenger -AGGAAAAAAAAGAAAGUCAUUAAdTdT |
|  | siRNA- 3  anti uc.8+ | Fw - UGUUUUCAGAGCAAAUAUGUCdTdT |
|  |  | Rv - CAUAUUUGCUCUGAAAACAACdTdT |
| Fishing | PNA1 uc.8+ | Biotin-HAx-CTGAAAACAACACAATAA-NH2 |
|  | PNA2 uc.8+ | Biotin-HAx-CAATAATTAGCAAAGGGGA-NH2 |
|  | PNA  scramble uc.8+ | Biotin-HAx-ACATAACATAACGAACAA-NH2 |
|  | siRNA-1  anti *CASZ1* | Guide - UUUAUCUGUGGGCAGUCCGUU |
|  |  | Passenger - AACGGACUGCCCACAGAUAAA |
| *CASZ1* silencing | siRNA-2  anti *CASZ1* | Guide - ACAGAGACACUGCAGAGAGUA |
|  |  | Passenger - UACUCUCUGCAGUGUCUCUGU |
|  | siRNA-3  anti *CASZ1* | Guide - AGAUAUAUAGAGAGAGAUAUG |
|  |  | Passenger - CAUAUGUGUGUGUAUAUAUCU |
| miR-596 competition | PNA-596 | AAGCCTGCCCGGCTCCTCGGG-R8 |
| Cellular  Localization | TO-PNA1 uc.8+ | TO- CTGAAAACAACACAATAA-R8 |

Abbreviations: RT-PCR, real-time polymerase chain reaction; RACE, rapid amplification of cDNA ends; miR, microRNA; uc, ultraconserved RNA; siRNA, small interfering RNA; PNA, peptide nucleic acid; Fw, forward; Rv, reverse.
